# Comprehensive insights into the mechanism of keratin degradation and exploitation of keratinase to enhance the bioaccessibility of soybean protein

**DOI:** 10.1186/s13068-023-02426-9

**Published:** 2023-11-17

**Authors:** Beiya Zhou, Yandong Guo, Yaju Xue, Xiuling Ji, Yuhong Huang

**Affiliations:** 1 College of Mathematical Sciences, Bohai University, Jinzhou, 121013 Liaoning China; 2 Beijing Key Laboratory of Ionic Liquids Clean Process, CAS Key Laboratory of Green Process and Engineering, State Key Laboratory of Multiphase Complex Systems, Institute of Process Engineering, Chinese Academy of Sciences, Beijing, 100190 China; 3 Huizhou Institute of Green Energy and Advanced Materials, Huizhou, 516000 Guangdong China

**Keywords:** Keratin degradation mechanism, *Bacillus* sp. 8A6, Soybean protein and hydrolysis, Transcriptome analysis

## Abstract

Keratin is a recalcitrant protein and can be decomposed in nature. However, the mechanism of keratin degradation is still not well understood. In this study, *Bacillus* sp. 8A6 can completely degrade the feather in 20 h, which is an efficient keratin degrader reported so far. Comprehensive transcriptome analysis continuously tracks the metabolism of *Bacillus* sp. 8A6 throughout its growth in feather medium. It reveals for the first time how the strain can acquire nutrients and energy in an oligotrophic feather medium for proliferation in the early stage. Then, the degradation of the outer lipid layer of feather can expose the internal keratin structure for disulfide bonds reduction by sulfite from the newly identified sulfite metabolic pathway, disulfide reductases and iron uptake. The resulting weakened keratin has been further proposedly de-assembled by the S9 protease and hydrolyzed by synergistic effects of the endo, exo and oligo-proteases from S1, S8, M3, M14, M20, M24, M42, M84 and T3 families. Finally, bioaccessible peptides and amino acids are generated and transported for strain growth. The keratinase has been applied for soybean hydrolysis, which generates 2234 peptides and 559.93 mg/L17 amino acids. Therefore, the keratinases, inducing from the poultry waste, have great potential to be further applied for producing bioaccessible peptides and amino acids for feed industry.

**Supplementary Information:**

The online version contains supplementary material available at 10.1186/s13068-023-02426-9.

## Introduction

More than 10 million tons of feather wastes are annually accumulated from the poultry farm [1]. These 90% underexploited and recalcitrant feather proteins have great potential to be converted to bioaccessible peptides and amino acids for supplementing the animal feed [2, 3]. Meanwhile, feathers are also the worth substrate to induce and produce the high-performance microbial keratinases, which can be further used in agriculture and diverse industries relating to leather, textile, pulp and paper, textile, detergent, fine chemical production, feed and food, cosmetic and medicine [4, 5]. Both the keratinolytic bioaccessible products and the multi-functional keratinases are in highly demand in the future sustainable and new bioeconomy era.

Feathers (composed of 32.2% of α-helix, 53.6% of β-sheets and 14.2% turns) [6] are classified as hard keratins with diversified morphological structures and numerous disulfide bonds [7]. Currently, keratinases such as alcalase, esperase, savinase, versazyme and prionzyme from S8 family are commercially available. However, a single type of protease cannot degrade keratin sufficiently. Meanwhile, keratinases from different microbial origins have related specific cleaving preferences. Qiu et al. [8] have concluded the bacterial and fungal keratinases including the endoproteases from S1, S8, S16, M4, M16, M36 families, exoproteases from S10, M14, M28, M38, M55 families and oligopeptidases from M3 and M32 families. AA11 lytic polysaccharide monooxygenases (LPMOs), found in fungal degradation systems, were identified to break down the glycosylation bonds between *N*-acetylglucosamine and serine or threonine in the non-coiled head structure of the keratin filament dimer [9, 10].

Unfortunately, feathers cannot be completely degraded by the sole synergistic effect of different types of keratinases. It has been reported that the blending of the purified recombinant keratinases cannot well degrade the feather [11, 12]. The microbial cells, on the other hand, can completely degrade the feather when using the feather as the carbon and nitrogen source [13–17]. Therefore, the microbial metabolites and the cell membrane system might have significant functions in the keratin degradation. Grumbt et al. [18] were the first to propose the mechanism of keratin degradation by dermatophytes which relied on the sulfite efflux pump to secrete the reducing agent sulfite from environmental cysteine via the action of the enzyme cysteine dioxygenase Cdo1. Then the sulphite released can weaken the keratin structure for further degradation by keratinases. In Jian Chen’s group, the fungal and bacterial keratin degradation process has been concluded into the factors of cell membrane, mechanical pressure, thiolysis and enzyme hydrolysis [19]. The thiolysis proposal has been further confirmed in the keratin degradation process by the engineered *B. licheniformis* BBE11-1 and its mutants, which showed that cysteine catabolism can mediate the catalytic procedure of keratinases via the secreting the sulfite [20]. The supplement of sodium sulfite to the cell-free keratinases can significantly improve the recycled keratin waste to amino acids [21]. Li et al. [22] were the first to analyze the transcriptome when the *Streptomyces* sp. SCUT-3 grew in the feather and indicated that the disulfide bond reduction is the first key step for feather degradation. Then the proteases hydrolyzed the feather to peptides and amino acids for the bacteria nutrients. Iron addition and aeration have been discussed to improve the feather degradation efficiency. These studies have mainly elucidated the thiolysis mechanism of keratin degradation. However, it is still unclear how the keratin degraders acquire nutrients and energy in an oligotrophic feather medium for rapid adaptation and proliferation in the early stage. Meanwhile, the full elucidation of the mechanism of sufficient keratin degradation is still needed for efficiently refining the keratin waste.

The efficient keratin degrader, *Bacillus* sp. 8A6, was recently identified to completely degrade feathers in our previous work [2]. This study was the first to comprehensively analyze the metabolic regulation process when this efficient keratin degrader *Bacillus* sp. 8A6 grew in the feather medium. In addition, soybeans are the major sources for food and feed proteins [23, 24]. However, the bioaccessibility of soybean proteins is limited by the trypsin inhibitors, antinutritional factors, glycinin and β-conglycinin and the cell wall structural integrity [25, 26]. Alkaline protease hydrolysis has been confirmed with the function to change the structure and conformation of soybean protein [27]. Therefore, the resulting stable and efficient keratinases from *Bacillus* sp. 8A6 were further applied for soybean protein hydrolysis. Relatively high amounts of bioaccessible peptides and amino acids after the degradation revealed the potential for applying the keratinase in feed industry.

## Materials and method

### Strain and culture condition

*Bacillus* sp. 8A6 was obtained from the Bacillus Genetic Stock Centre at Ohio State University (Biological Sciences 556 484 W. 12th Ave Columbus, OH 43210-1214). The LB medium (10 g/L tryptone, 5 g/L yeast extract and 10 g/L NaCl) was used for cell growth. The feather medium contained 0.75 g/L NaCl, 1.75 g/L K_2_HPO_4_, 10 mM MOPS, 0.25 g/L MgSO_4_·7H_2_O, 0.055 g/L CaCl_2_, 0.010 g/L FeSO_4_·7H_2_O, 0.005 g/L ZnSO_4_·7H_2_O and different concentration of feathers (0.5, 1, 2, 3, 4, 5, and 10% (w/v)).

### RNA extraction and synthesis of cDNA

After the single-factor and response surface method (RSM) optimization, the optimal conditions were inoculation amount of 1.83 mL, feather concentration of 3.905% (w/v) and pH 9.21 (see Additional file 1: Supplemental Materials and Method; Supplemental Results and Discussion), *Bacillus* sp. 8A6 was inoculated into the sterile feather medium and growth under the optimal conditions for 0, 8, 20 h. Cells were collected for RNA extraction. The cells from three time points were mixed and RNA was extracted from the mixture. After grinding cells into powder with liquid nitrogen, RNA was extracted by the instruction of the RNAprep pure Bacteria Kit (DP430, Tian gen). Then, the RNA was reverse transcribed to cDNA using reverse transcriptase (TAKARA, RR036A) according to the manufacturer's instructions. The reaction system was as follows: 4 μl 5X PrimeScript RT Master Mix (Perfect Real Time), 10 μl total RNA, and 6 μl RNase Free ddH_2_O. The reaction conditions were as follows: 37 °C for 15 min, 85 °C for 5 s and then 4 °C.

### Selection of internal reference genes and design of real-time fluorescence quantitative PCR (RT-qPCR) primers

According to Chen’s research [28], 16sRNA was selected as the internal reference gene. RT-qPCR was used to determine the transcript levels of 7 differentially expressed genes (DEGs) from the S8, S9 and S1A families. Primer Premier 5.0 was used to design RT-qPCR primers, which were synthesized by Sangon Biotech (Shanghai) Co. (Additional file 1: Table S1).

### RT-qPCR reaction

The prepared cDNA was used as a template for the RT-qPCR reaction using TB Green® Premix Ex Taq™ II (Tli RNaseH Plus) (TAKARA, RR820A). The reaction system was as follows: 10 μl TB Green® Premix Ex Taq™ II, 1 μl each of forward and reverse primers, 1 μl cDNA, and 7 μl ddH_2_O. RT-qPCR cycling conditions were 95 °C for 30 s, followed by 40 cycles at 95 °C for 5 s, 55 ℃ for 30 s and 72 °C for 20 s, at last, the melting curve was generated at 95 °C for 15 s, 65 °C for 60 s and 95 °C for 15 s. Each reaction was performed 3 times. Using 16sRNA as an internal reference gene, the relative expression level of each gene was calculated using 2^−ΔΔCT^ method.

### Transcriptome analysis

The RNA was sent to Genewiz company (Suzhou, China) for Illumina sequencing. The raw data were filtered by Cutadapt (v 1.9.1) and mapped to the *Bacillus* sp. 8A6 genome sequences (Genbank: accession number QFZE00000000) via software Bowtie2 (v2.2.6) [29]. Then HTSeq (v0.6.1p1) [30] was used to estimate gene expression levels from the pair-end clean data. Differential expression analysis used the DESeq2 Bioconductor package, a model based on the negative binomial distribution [31, 32], and sequencing depths at the 0 h, 8 h, and 20 h were 1227X, 1391X and 1283X, respectively. After being adjusted by Benjamini and Hochberg’s approach for controlling the false discovery rate, Padj of genes were set < 0.05 to detect differential expressed ones. Sequencing data quality assessment was analyzed using the software FastQC (v0.10.1). In general, the sequencing error rate should be less than 0.5% at each base position. If the sequencing error rate was denoted by e and the base quality value of Illumina sequencing was denoted by Qphred, the Qphred = − 10log10(e), low-quality data were then filtered and contamination and splice sequences were removed by the software Cutadapt (version 1.9.1). GO enrichment analysis method was Goseq [33], which was based on Wallenius non-central hyper-geometric distribution. Compared with the ordinary hyper-geometric distribution, this distribution was characterized by the difference between the probability of extracting an individual from a certain category and the probability of extracting an individual from outside a certain category. The difference in this probability was obtained by estimating the preference of gene length. Thus, the probability of GO term enrichment by differential gene can be calculated more accurately. The GOSeq (v1.34.1) was used to identify Gene Ontology (GO) terms that annotate a list of enriched genes with a significant* p*-value less than 0.05. And topGO was used to plot DAG. The Kyoto Encyclopedia of Genes and Genomes (KEGG) web server was used to extract enzyme codes and the KEGG pathways. The software used in this analysis was EdgeR. The results were measured according to the difference significance criteria (differential gene expression changes of more than 2 times and *q*-value less than 0.05) for screening. Cluster of Orthologous Groups of proteins (COG) database was used to predict the function of proteins or protein assemblies [34]. COG classification of the major distribution of differential genes was obtained through annotation and classification analysis of COG database [35].

### Analysis of protease activity

The protease activity was analyzed by using Azo-casein as substrate as described with few modifications [2]. 20 μl 1.5% (w/v) azo-casein in 50 mM Tris–HCl buffer (pH 9) as substrate and 20 μl crude enzyme solution (diluted fivefold with 50 mM Tris–HCl buffer pH 9.0) were mixed and incubated at 60 °C for 15 min at 500 rpm. One arbitrary unit (U) of protease activity is defined as the amount of enzyme that causes an increase in absorbance of 0.01 between the sample and the control at 405 nm under the assay conditions [2].

### Analysis of keratinase activity

The keratinase activity was detected as described [36]. 0.4 g keratin azure were cut into pieces and suspended in 100 mL 50 mM sodium carbonate buffer (pH 9.0). 500 μl keratin azure suspension and 500 μl enzyme solution were then mixed to react at 50 °C for 24 h at 1000 rpm. One unit of keratinase activity was defined as the amount of enzyme that caused an increase of 0.01 absorbance at 595 nm.

### Optimum pH and temperature of protease

The optimum pH of protease was determined according to the method of protease activity analysis described above with some modifications that crude enzyme solution was diluted by buffer at a pH range from 6 to 10 (50 mM sodium acetate buffer pH 6, 50 mM Tris–HCl buffer pH 7–9, and 50 mM sodium carbonate buffer pH 10). And the substrate Azo-casein (50 mM) was also prepared with buffers at pH 6–10.

Similarly, in the determination of the optimum temperature of enzymes, diluted crude enzyme solution and azo-casein were prepared by above optimum pH buffer. The reaction system was incubated at the range of 30–65 °C.

### Tolerance of pH and temperature on protease activity

The crude enzyme solution was diluted 2.5 times with buffers of pH 6–11 (50 mM sodium acetate buffer pH 6, 50 mM Tris–HCl buffer pH 7–9, 50 mM sodium carbonate buffer pH 10, and 50 mM sodium carbonate buffer pH 11) and incubated in the 4 °C for 1 h or 4 h. And then diluted it 2 times again for the analysis of the residual activity of protease by Azo-casein as substrate. Similarly, the crude enzyme solution diluted 2.5 times with 50 mM pH 9 Tris–HCl buffer was incubated at the temperature range of 30–65 °C for 1 h or 4 h and the residual activity of protease was measured as above.

### Morphological analysis of the degraded feathers by scanning electron microscopy (SEM)

The surface morphology at different times in the degradation process of feathers was investigated by SEM, including 0 h, 8 h and 20 h. The feather culture was harvested at 0 h, 8 h and 20 h, and the feather was washed three times with phosphate buffer saline (50 mM, pH 7.2). The remaining steps of sample preparation follow the literature [37].

### Degradation of soy protein isolate (SPI), peptides and free amino acids analysis

The biodegradability of SPI was investigated. The SPI degradation system contained 1 g SPI and 2 mL *Bacillus* sp. 8A6 keratinase or NY100 with the same enzyme activity, then the system was suspended with 10 mL sodium carbonate buffer (pH 9) and incubated at 60 °C for 1 h with shaking at 250 rpm. Besides, the system without *Bacillus* sp. 8A6 keratinase or NY100 was used as a negative control. After centrifugation at 12,000 rpm for 10 min, the supernatant was analyzed for amino acids and short chain peptides by Nano LC–MS/MS in Standard Testing Group Co. Ltd. (Qingdao, China).

### Statistical analysis

The statistical analyses were executed by GraphPad Prism 8.3.0. In this experiment, the mean ± standard deviation (SD) of more than three sets of data were compared, and checked the significance between each of the two groups by one-way ANOVA. *p < 0.05 was regarded as statistically significant.

## Results and discussion

### Comprehensive transcriptome analysis for *Bacillus* sp. 8A6 grown in feather medium

#### RNA preparation and transcriptome sequencing

**Fig. 1:**
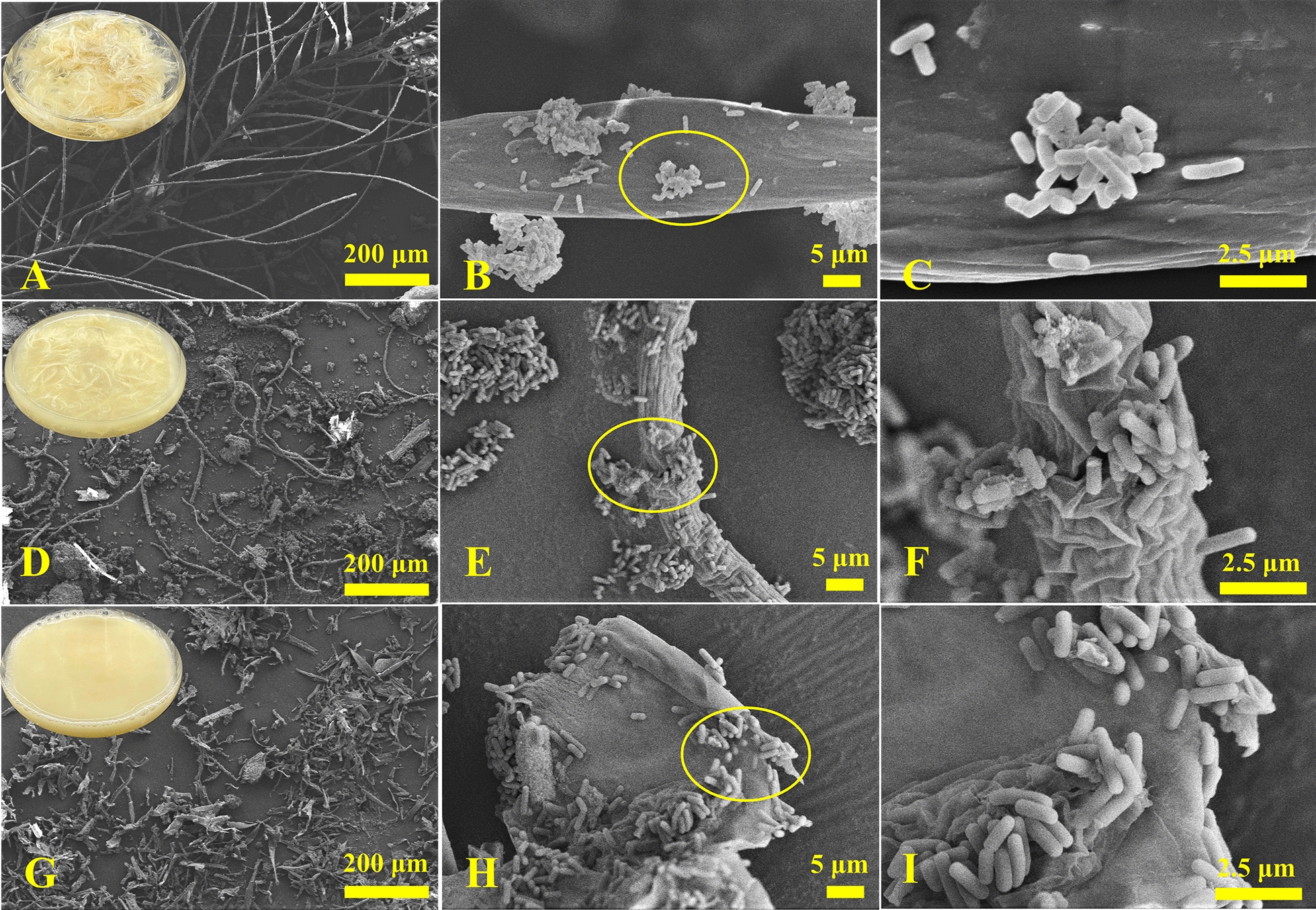
The morphological changes of feather degradation by *Bacillus* sp. 8A6 under the optimized conditions were observed by scanning electron microscope. The first row is initial incubation (0 h) (**A**–**C**), the second row is 8 h incubation (**D**–**F**) and the third row is 20 h incubation (**G**–**I**)

After single-factor and RSM optimization of the growth conditions of *Bacillus* sp. 8A6 in the medium with feather as the sole carbon and nitrogen source (Additional file 1: Supplemental Results and Discussion, Table S2, Fig S1). The feathers were all decomposed within 20 h (cultures in flask in Fig. 1, Additional file 1: Supplemental Results and Discussion), which is the efficient keratin degrader reported so far when compared with the talented keratin degraders *Amycolatopsis keratiniphila* subsp. *keratiniphila* D2T, which can degrade feathers within 3 days [38, 39]; and *Ectobacillus* sp. JY-23 [40], *Bacillus* species such as *Bacillus* sp. CN2 (48 h) [41], *B. licheniformis*, *B. subtilis*, *B. cereus*, *B. thuringiensis*, *B. aerius*, *Bacillus* sp. CL18, etc. (24–84 h degradation with extra nitrogen source in the medium) [42–47], *Pseudochrobactrum* sp. IY-BUK_1_ (3 days), and even microbial consortia KMCG6 (3 days) [48], etc. Meanwhile, the keratinase activity of *Bacillus* sp. 8A6 achieved 136.5 U/mL and it was considerably higher than that of other strains [36, 49–51]. SEM showed that most of the feathers’ barbules and barbs have been degraded after 8 h of inoculation (Fig. 1A–F). It was further shown that the strains can work on the fracture section of the rachis for the feather degradation (Fig. 1E–F). After 20 h inoculation, the strains were enriched on the small feather chips to achieve the comprehensive degradation of the feather (Fig. 1G–I). According to the morphological changes of feathers when *Bacillus* sp. 8A6 grown in the feather medium and the growing properties in LB medium (Additional file 1: Fig S2). Therefore, the samples when *Bacillus* sp. 8A6 grew in feather medium for 0, 8, and 20 h were prepared for RNA extraction and transcriptome sequencing for revealing the keratin degradation mechanism.

After transcriptome sequencing, the raw reads (Additional file 1: Table S3) were cleaned. 4.446, 5.063 and 4.169 Gbp of data in total were obtained and 98.57%, 97.49% and 98.71% reads were mapped to the genome sequences for the *Bacillus* sp. 8A6 before (0 h) and after being inoculated in the feather medium for 8 h and 20 h, respectively (Additional file 1: Table S4). Finally, 3863 genes were identified. The Pearson correlation coefficient and principal component analysis indicated that after inoculation in the feather medium for 8 h and 20 h, the transcripts changed greatly (Additional file 1: Fig S4A and S4B). According to FPKM statistics, the density of the 8 h inoculation of *Bacillus* sp. 8A6 was higher than that of the 20 h inoculation followed by the original culture. 1006 genes up-regulated and 790 genes down-regulated after 8 h incubation (Additional file 1: Fig S5A). Then 412 genes were up-regulated and 503 genes down-regulated after 20 h incubation (Additional file 1: Fig S5B). The hierarchical clustering showed an obvious change trend that most of the genes of the first two clusters were up-regulated obviously when *Bacillus* sp. 8A6 was grown in feather medium for 8 h. These genes should be important for strain inoculation on the feather, keratin decomposition, nutrients transportation and absorption (Additional file 1: Fig S6).

#### Overall analysis of transcriptome

**Fig. 2:**
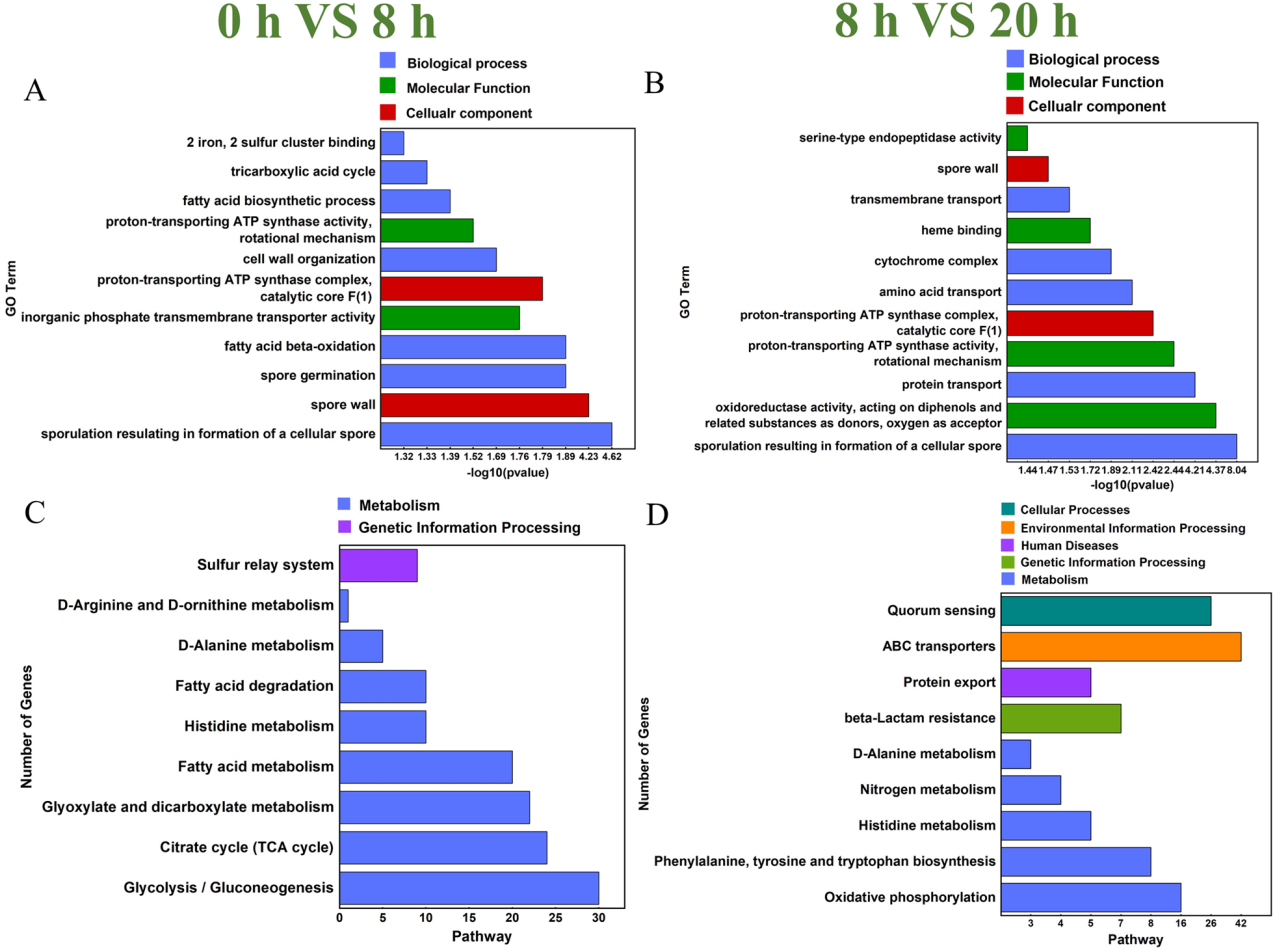
Gene Ontology (GO) analysis of the molecular function, cellular component and biological process for the groups 0 h VS 8 h (**A**) and 8 h VS 20 h (**B**) according to the -log_10_(*p*-value) when *Bacillus* sp. 8A6 grew in feather medium. The higher −log_10_(*p*-value) indicates the more significant enrichment of the GO term. Kyoto Encyclopedia of Genes and Genomes (KEGG) analysis of the pathways for the groups 0 h VS 8 h (**C**) and 8 h VS 20 h (**D**) according to the −log_10_(*p*-value) when *Bacillus* sp. 8A6 grew in feather medium. Meanwhile, the group 0 h VS 8 h meant the comparisons of gene expression between 0 and 8 h. The group 8 h VS 20 h meant the comparisons of gene expression between 8 and 20 h. The most significantly different pathways are shown according to the Q-value. Other significantly different pathways are shown in Additional file 1: Fig. S7

After inoculation, the *Bacillus* sp. 8A6 needs to survive in the oligotrophic and insoluble feather medium without any readily accessible carbon and nitrogen source, which is different from the nutrition-rich LB medium. GO analysis (Additional file 1: Fig S7) showed that functional genes related to sporulation and cell wall regulation were expressed and enriched in the following genes (Fig. 2A). During growth and proliferation, *Bacillus* sp. 8A6 cells had high energy requirements, as shown by the log10(p-value) of fatty acid β-oxidation, proton-transporting ATP synthase, inorganic phosphate transmembrane transporter and iron uptake. According to the FPKM statistics, the sulfur cluster binding functional genes were also enriched during this period, which might relate to the sulfite synthesis and exportation for breaking down the feather disulfide bonds. The resulting loose feather could be easily accessed and further hydrolyzed by the induced proteases/peptidases. When the cells grew in the feather medium for 20 h, the GO terms changed greatly (Fig. 2B). The cells continuously required tremendous energy and oxygen for cell growth, metabolism and proliferation through the oxidoreductase, proton-transporting ATP synthase and complex, cytochrome, iron binding and aerobic respiration. Meanwhile, the serine endopeptidases and aminopeptidases were enriched for complete feather degradation. The genes for mass-transportation were enriched obviously during this stage, such as the protein, amino acid, proton, transmembrane transport and nitrate assimilation, which are mainly responsible for up-taking the feather degraded soluble peptides and amino acids.

The KEGG analysis showed that after *Bacillus* sp. 8A6 inoculation for 8 h, the cells were mainly colonized on the feather for growth. The result showed that the sulfur relay system was active when the cells grew in the feather medium (Fig. 2C). The up-regulated ThiS ubiquitin-like proteins (Ubls) were a key sulfur carrier protein and signaling messenger that control cell proliferation [52]. When the cells grew on the feather medium from 8 to 20 h, the feather has been degraded and the amino acids metabolism has been up-regulated obviously, including the phenylalanine, tyrosine, tryptophan, histidine, D-alanine, together with the nitrogen metabolism (Fig. 2D). The substantial difference was that the gene for protein export and ABC transporters were highly induced during this stage. It indicated that the cells were extremely active to consume the degraded soluble peptides and amino acids for growth. The COG analysis further identified 113 up-regulated genes for amino acids transport and metabolism after 8 h inoculation, which was followed by 16 related up-regulated genes after 20 h inoculation (Additional file 1: Figs. S8 A and B).

### Mechanism for* Bacillus* sp. 8A6 growth and feather degradation

#### Energy and nutrients for *Bacillus* sp. 8A6 surviving in feather medium at the early stage

**Fig. 3:**
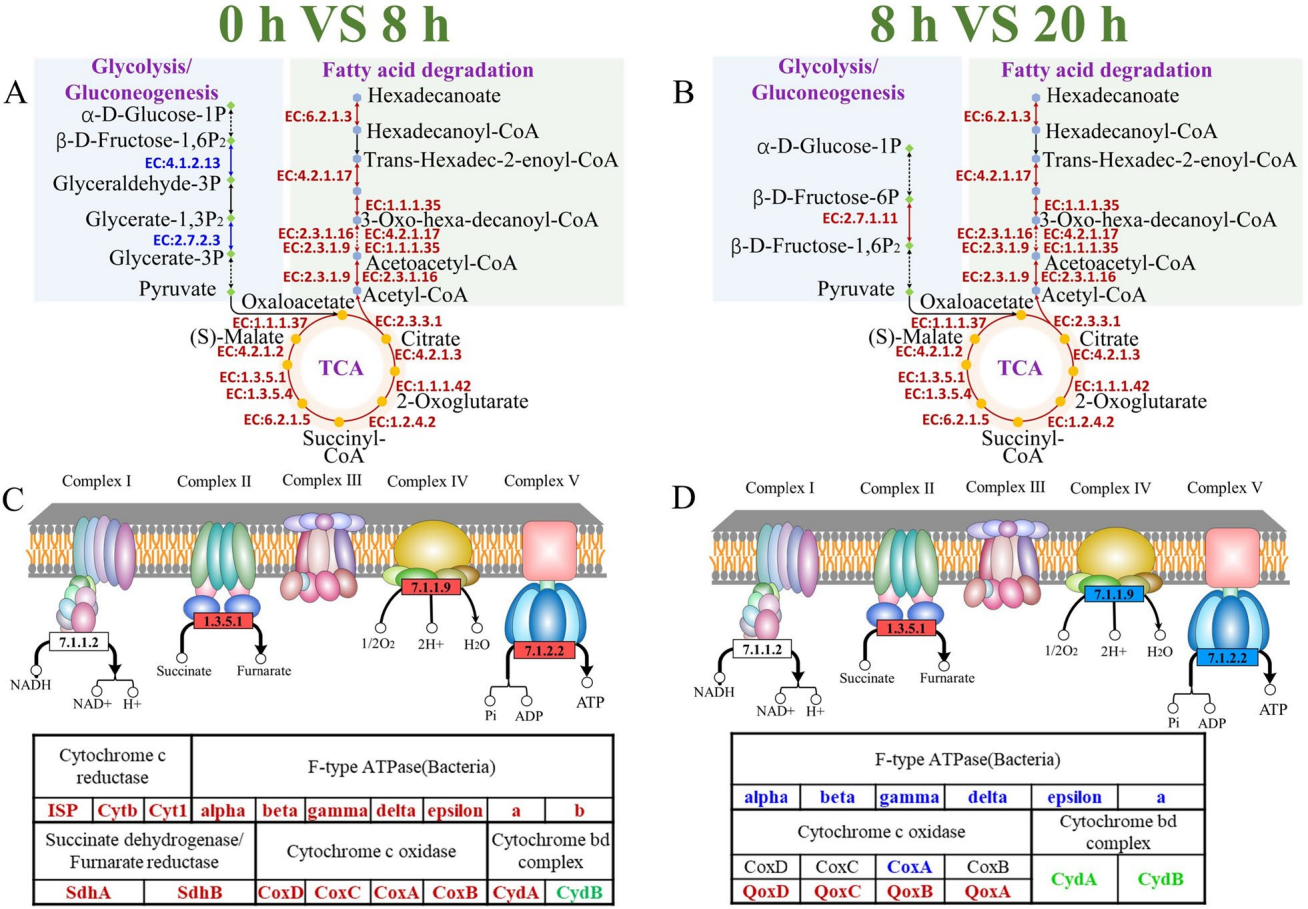
Fatty acid degradation, TCA cycle (**A**, **B**) and oxidative phosphorylation (**C**, **D**) analyses for the groups 0 h VS 8 h and 8 h VS 20 h when *Bacillus* sp. 8A6 grew in feather medium, respectively. Meanwhile, the group 0 h VS 8 h meant the comparisons of gene expression between 0 and 8 h. The group 8 h VS 20 h meant the comparisons of gene expression between 8 and 20 h. The up-regulated, down-regulated and unchanged genes are indicated as red, blue and green colors, respectively

The *Bacillus* sp. 8A6 can grow in the optimized feather medium only including feather and trace minerals. The question of how the strain starts to use such resistant polymers as carbon and nitrogen source for growth and proliferation is still unelucidated. In this study, all the genes for fatty acid (hexadecanoate) degradation were identified and all of them were up-regulated (Fig. 3). It has been reported that the surface of feathers is rich in lipids (5%) [53]. The outer lipid layer might consist of hexadecanoate along with other fatty acids to bound through thioester linkages to the keratin proteins, which was similar to the wool keratin as described by Ghosh et al. [54]. Li et al. [55] reported that fatty acid metabolism had significant up-regulated expression. This study confirmed that when *Bacillus* sp. 8A6 inoculated in the feather medium for 8 h, the strain up-regulated the fatty acid degradation pathways (Fig. 3A). The resulting acetyl-CoA was then involved in the TCA cycle, which was also highly active for all the enzymes (Fig. 3A). The intermediates of the active TCA cycle can further stimulate other metabolic pathways, providing necessary energy and nutrients for the growth and proliferation of the *Bacillus* sp. 8A6. During the same period, the genes of the oxidative phosphorylation in complex II (succinate dehydrogenase, SdhA & SdhB), complex IV (cytochrome c oxidase, CoxA, CoxB, CoxC & CoxD), complex V (F-type ATPase, F1 unit (α, β, γ, δ, ε) and F0 unit (a & b)) were also up-regulated when the strain grew in the feather medium for 8 h. The complex II & IV can push the protons across the cell membrane into the cytoplasm, resulting in the formation of electromotive force which can further provide the free energy for ATP synthesis by the up-regulated complex V. Therefore, when the *Bacillus* sp. 8A6 started to grow in the feather medium, the oxidative phosphorylation was stimulated to provide sufficient ATP for cell growth and metabolism (Fig. 3C). After the breakdown of outer lipid layer and the exposure of internal keratin, the fatty acid regulation, TCA cycle and oxidative phosphorylation were down-regulated when the strains grew in the feather medium for 20 h (Fig. 3B and D). Therefore, the fatty acid degradation, TCA cycle and oxidative phosphorylation were the key first steps for providing energy and nutrients for strain survival and growth in the feather medium. Meanwhile, the degradation of the outer lipid layer of feather can assist in exposing the internal keratin structure to further degradation.

#### New sulfite metabolic pathway for breaking down the disulfide bonds

**Fig. 4:**
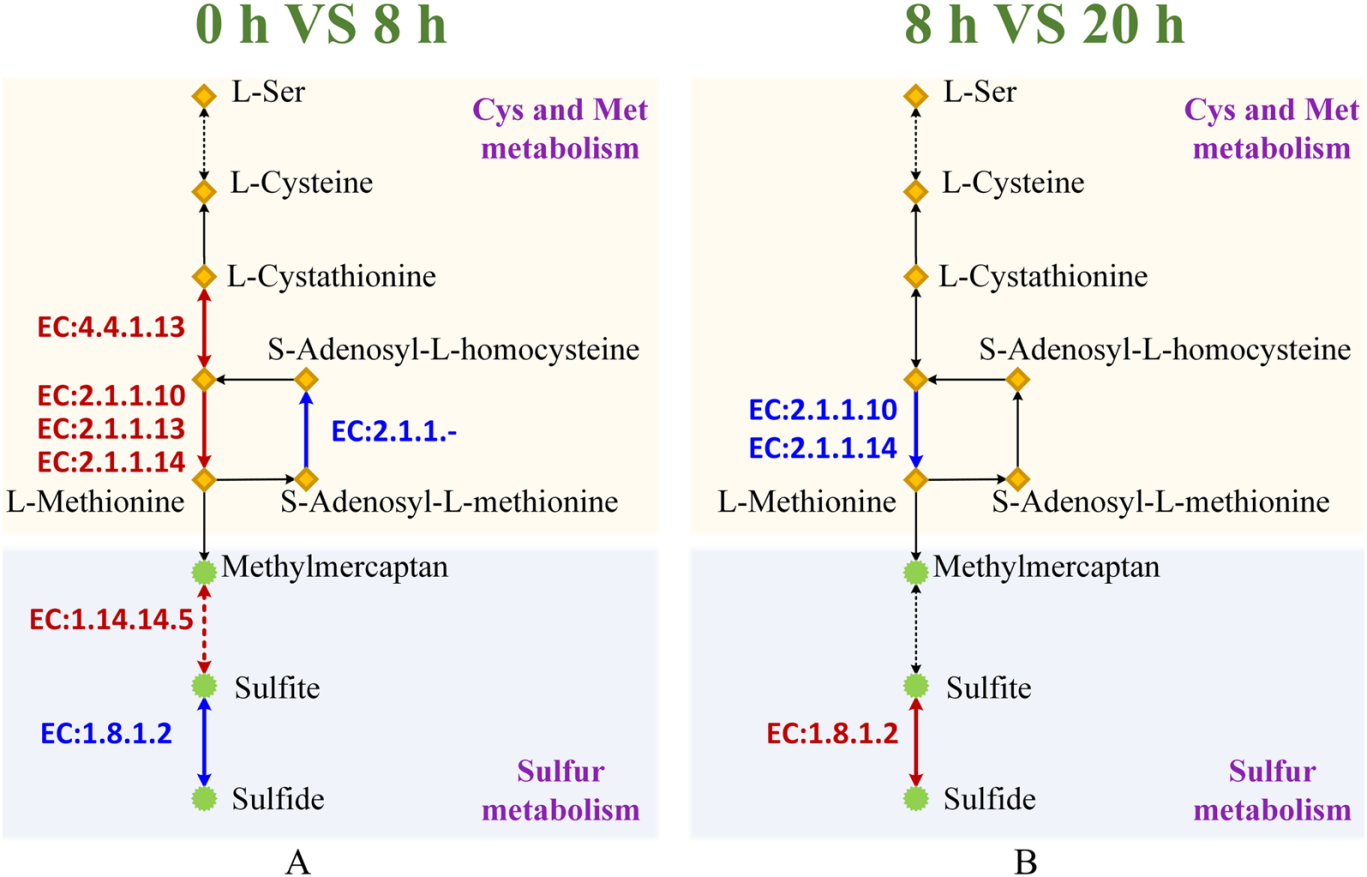
Sulfite metabolism for the groups 0 h VS 8 h and 8 h VS 20 h when *Bacillus* sp. 8A6 grew in feather medium. Meanwhile, the group 0 h VS 8 h meant the comparisons of gene expression between 0 and 8 h. The group 8 h VS 20 h meant the comparisons of gene expression between 8 and 20 h. The up-regulated and down-regulated genes are indicated as red and blue color, respectively

The main challenge for sufficient keratin decomposition is the abundant cross-linked disulfide bonds in/among the keratin filaments [56]. It is necessary to reduce the disulfide bridges to weaken the rigid structure. This study was the first to identify a different sulfite metabolism pathway after the *Bacillus* sp. 8A6 being inoculated in the feather medium for 8 h (Fig. 4A). l-Cysteine was converted to l-cystathionine by cystathionine-γ-synthase. Then the key enzymes including the β-cystathionase (EC4.4.1.13), homocysteine S-methyltransferase (EC2.1.1.10), methionine synthase (EC2.1.1.13) and 5-methyltetrahydropteroyltriglutamate-homocysteine S-methyltransferase (EC2.1.1.14) were highly up-regulated for l-methionine synthesis. The l-methionine was further converted to methylmercaptan and sulfite by the up-regulated alkanesulfonate monooxygenase (EC1.14.14.5). Meanwhile, the sulfite reductase (NADPH) (EC1.8.1.2) which was down-regulated for sulfite accumulation. The sulfite was then exported to exocellular to lose the feather structure. When the cells were inoculated into the feather medium for 20 h, the sulfite metabolism was down-regulated and the sulfite was converted to sulfide (Fig. 4B). Therefore, the sulfite accumulation and exportation were crucial during the beginning stage of keratin degradation. The synergistic factor sulfite for keratin degradation has been confirmed by mixing keratinase and sodium sulfite in vitro for complete feather degradation [20, 22]. Grumbt et al. [18] have proposed the model for keratin degradation by dermatophytes which were mainly dependent on the cysteine dioxygenase (Cdo1) and sulfite efflux pump (Ssu1). The route further cleared the cysteine catabolism that aspartate aminotransferase (Ast1) was important for deamination after the Cdo1 oxidation [20]. Two cysteine dioxygenase genes (*cdo1* and *cdo2*) were identified and up-regulated. Meanwhile, the sulfite exporter gene *tauE* instead of sulfite efflux pump gene (*ssu*) was found when bacteria *Streptomyces* sp. SCUT-3 grew in feather medium [22]. However, only 4 aspartate aminotransferase genes were identified in the 4 pathways when *Bacillus* sp. 8A6 grew in feather medium, the 4 pathways are purine metabolism, 2-oxocarboxylic acid metabolism, microbial metabolism in diverse environments and glycine, serine and threonine metabolism. The new up-regulated sulfite metabolic pathways for *Bacillus* sp. 8A6 in our study could further provide potential strategies for construction efficient engineering *Bacillus* strains for keratin decomposition.

It has been reported that disulfide reductases can degrade disulfide bonds in keratin [8, 57]. In this study, 4 disulfide reductases (3 from TlpA family and 1 from CoA family) were up-regulated when the *Bacillus* sp. 8A6 grew in the feather medium for 8 h (Table 1 and Additional file 1: Table S5). After 20 h growth, 1 TusA family sulfurtransferase was up-regulated (Additional file 1: Table S6). The synergistic effect of disulfide reductases and keratinases has been confirmed by blending these two types of enzymes [58, 59]. However, the keratinolytic disulfide reductases from the efficient degraders have not yet been well exploited and characterized currently.

**Table 1:** Potential proteases and disulfide reductases for keratin decomposition

Type	geneID	Family	Enzymes
Potential keratinolytic proteases	RS14955	S1	Trypsin-like serine protease
	RS05690	S8	Peptidase
	RS15255	S8	Peptidase
	RS02400	S9	Acetylxylan esterase
	RS16285	S8	Serine peptidase
	RS11220	S9	Peptidase
	RS18510	S8	Serine peptidase
	RS08615	S8	Serine peptidase
	RS13920	S8	Serine peptidase
	RS01465	S8	Serine peptidase
	RS08645	S9	Chitobiase/beta-hexosaminidase
	RS17315	S8	Peptidase
	RS17960	M3	Oligoendopeptidase F
	RS00915	M14	Peptidase
	RS05045	M14	Hypothetical protein
	RS08135	M20	Peptidase T
	RS02515	M20	Dipeptidase PepV
	RS13215	M20	Aminohydrolase
	RS08570	M24	Aminopeptidase
	RS03115	M42	Metallopeptidase
	RS08920	M84	Zinc-dependent metalloprotease
	RS17575	T3	Gamma-glutamyltransferase
Disulfide reductases	RS17050		TlpA family disulfide reductase
	RS14160		TlpA family disulfide reductase
	RS05595		CoA-disulfide reductase
	RS14855		TlpA family disulfide reductase

#### Diverse proteases for keratin decomposition

**Fig. 5:**
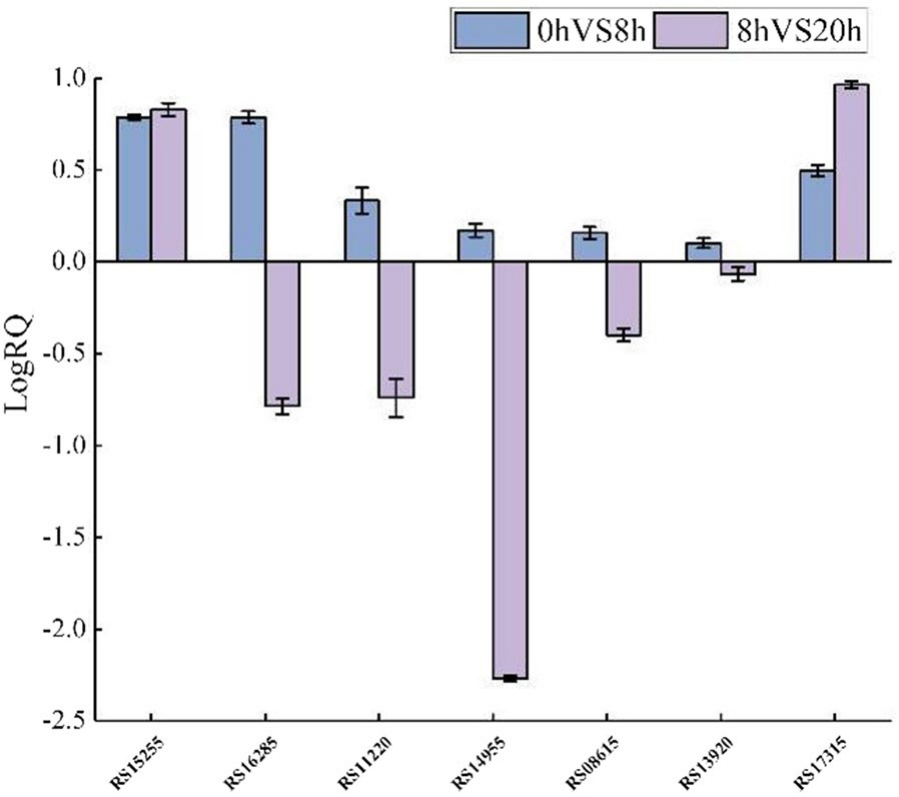
Comparison of seven DEGs and RT-qPCR. The data from this study are expressed as mean ± SD of three parallel determinations

After *Bacillus* sp. 8A6 growing in feather medium for 8 h, 52 up-regulated proteases were identified from metalloproteases (M3, 2M14, M15, 4M20, 3M23, M24, M42, M56, 2M78, M84), cysteine proteases (C39, 2C40, C60, C82), serine proteases (S1, 8S8, 3S9, 2S11, S13, S14, S16, S41, 2S66), unknown proteases (U57), glutamic proteases (2G5), aspartic proteases (2A25, A36), threonine proteases (T3), and 3 unclassified proteases (Additional file 1: Table S5). When the strain grew in the feather medium for 20 h, 15 proteases from metalloproteases (M24, M50, M78), cysteine proteases (C40, 2C56, C60), serine proteases (3S8, S24, S26), and 3 unclassified proteases (Additional file 1: Table S6). Seven genes from different families validated RNA-seq results using RT-qPCR analysis. The results showed that all seven genes were up-regulated at 8 h compared to that of control, and at 20 h, RS16285, RS08615 from S8 family, RS11220 from S9 family, and RS14955 from S1A family were down-regulated at 20 h (Fig. 5). The RT-qPCR statistics of these 7 differential genes were consistent with the RNA-seq sequencing results. Functional analysis further showed that some proteases were mainly related to lysing cell wall (such as proteases from M15, M23, M56, S11, S13, S66, C40, C60, C82 families), sporulation (such as proteases from A25 and A36 families), and cleaving the ImmR protein from M78 family. Therefore, after comprehensive analysis of the function of the up-regulated proteases after the cells growing in the medium for 8 and 20 h, 4 disulfide reductases and 22 proteases from S1, S8, S9, M3, M14, M20, M24, M42, M84, and T3 families have the great potential for synergistic keratinolysis (Table 1). The identified 3 significantly up-regulated S9 peptidase/acetylxylan esterase/chitobiase/beta-hexosaminidase might have function on the glycoprotein groups, especially on the head domain of the polypeptide monomer of the keratin structure. Then the charge of the filament head structure will be changed [9], resulting in the de-assembly of the keratin filaments. The disassembled keratin can be further hydrolyzed by the synergistic proteases as follows. Recently, the commercial and the most studied keratinases were from S8 family [8, 9]. In our study, eight S8 proteases have great potential for keratin decomposition. S1 family proteases are endoproteases with a classic catalytic triad, which also contributed to the keratin degradation. Two keratinolytic S1 serine proteases have been identified by mass spectrometric peptide mapping and purified from* A. keratinophila* subsp. keratinophila D2T [60]. Our previous secretome analysis of *Bacillus* sp. 8A6 also indicated that this S1 trypsin-like serine protease was up-regulated when it grew in the pig bristle and feather medium [2]. Other keratinolytic proteases in S1 family originated from *Paenarthrobacter nicotinovorans* [61], *Nocardiopsis* sp. TOA-1 [62], *Streptomyces fradiae* var. k11 [63] and *S. albidoflavus* [64] were also characterized. This study further identified that the M3 oligoendopeptidase was significantly up-regulated in this bacteria species as the keratinolytic M3 oligopeptidase from the fungus *Onygena corvina* [3]. The metalloaminopeptidases (M42), exo-metallopeptidase (M14, M20, M24) and endo-metallopeptidase (M84) were the first discovered and have great potential to participate in the keratin decomposition. Meanwhile, the γ-glutamyltransferase from T3 family was both up-regulated significantly in *Bacillus* sp. 8A6 secretome [2] and transcriptome when the strain grew in the keratin (bristles and feather) medium, indicating that the γ-glutamyltransferase was important to balance the levels of intracellular cysteine [65, 66] for further sulfitolysis of feather [67]. Therefore, this study can provide a shortcut to develop efficient keratinolytic enzymes including serine proteases, metallopeptidase, γ-glutamyltransferase and disulfide reductases. The advanced protein engineering and heterogenous expression might further improve the keratinolytic properties for industrial and commercial application [4].

#### Further absorption of peptides and amino acids after keratin degradation

**Fig. 6:**
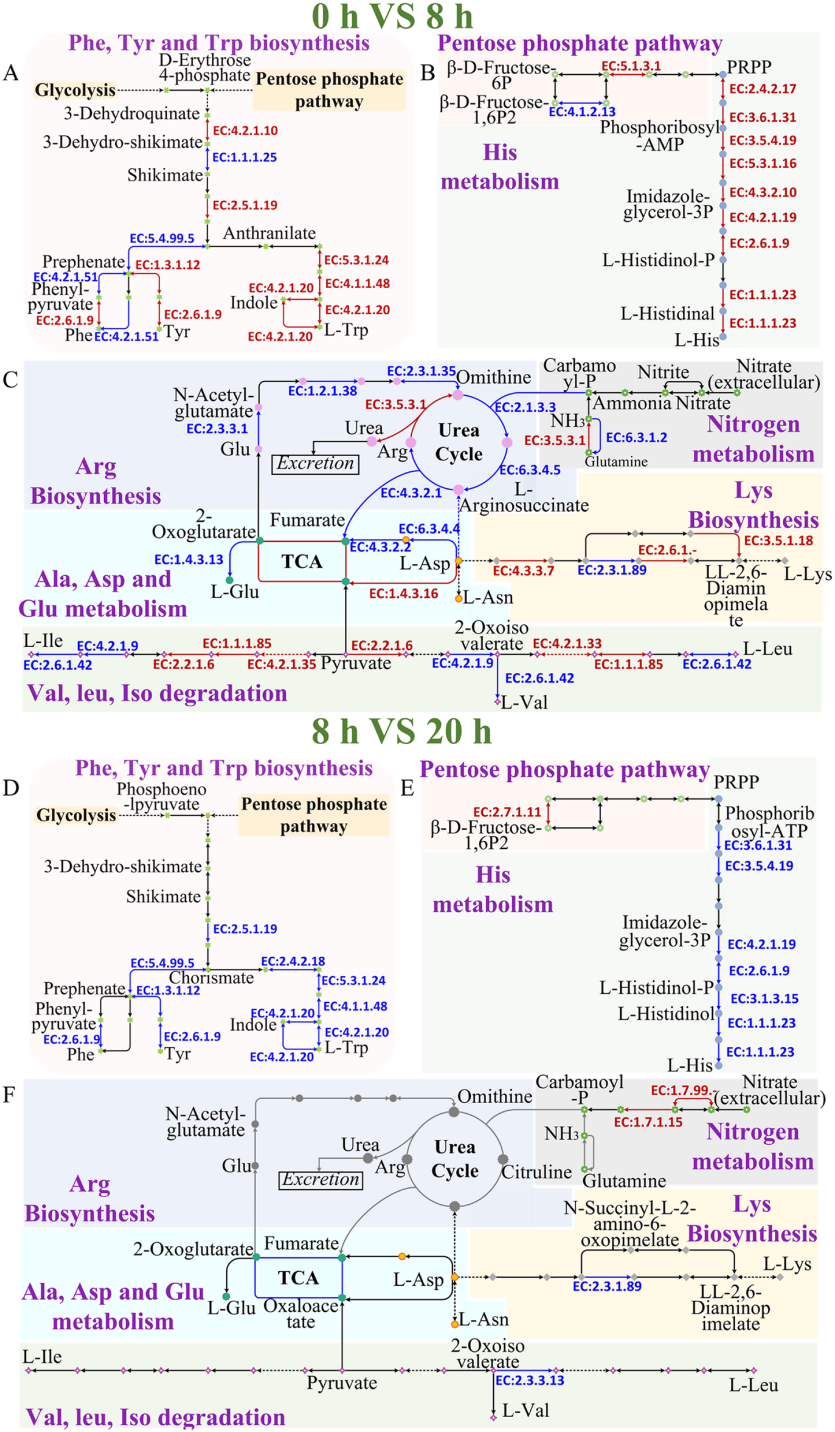
Amino acid, nitrogen and urea metabolism for the groups 0 h VS 8 h and 8 h VS 20 h when *Bacillus* sp. 8A6 grew in feather medium. Meanwhile, the group 0 h VS 8 h meant the comparisons of gene expression between 0 and 8 h. The group 8 h VS 20 h meant the comparisons of gene expression between 8 and 20 h. The up-regulated and down-regulated genes are indicated as red and blue color, respectively

The soluble peptides and amino acids were generated after the synergistic effects of keratinolytic proteases. These bioaccessible peptides and amino acids can be further absorbed into the cell for growth and metabolism by specific transporters. During the early stage of *Bacillus* sp. 8A6 grew in the feather medium, all the genes (Peb1A, Peb1B and Peb1C) for the aspartate/glutamate/glutamine transporter, gene (TcyM) for cystine transporter and genes (DppE, DppC and DppD) for dipeptide transporter were up-regulated. Meanwhile, the biotin transporter (BioY, EcfT and EcfA1) and the iron transporter (MtsA, MtsC and MtsB) were also up-regulated. Li et al. [22] have confirmed that the iron uptake can improve keratin degradation. Amino acid, nitrogen and urea metabolism have been further comprehensively analyzed in this study. The pathways for aromatic amino acids and histidine metabolism were analyzed when *Bacillus* sp. 8A6 inoculated in the feather medium for 8 h. The enzymes such as 3-dehydroquinate dehydratase (EC4.2.1.10) and 3-phosphoshikimate 1-carboxyvinyltransferase (EC2.5.1.19) were up-regulated for shikimate and chorismate synthesis after the active pentose phosphate pathway. Then the key enzymes for all the aromatic amino acids (phenylalanine, tyrosine and tryptophan) were equally up-regulated (Fig. 6A and D). Meanwhile, the alkaline histidine biosynthesis pathway was also up-regulated obviously (Fig. 6B and E). The early stage of the inoculation on feather medium can also activate the intermediates synthesis of pyruvate group amino acids including valine, leucine and isoleucine. The cysteine and methionine metabolisms have been active at the early stage to provide enough sulfite for weakening the keratin structure as discussed above.

Kang et al. [48] have indicated that the urea cycle might boost the keratinolytic efficiency and be involved in the keratin decomposition process through the metagenome analysis. In our study, all the genes for the urea cycle have been identified, however, most of them were down-regulated at the early inoculation stage (Fig. 6C). After the strain grew in the feather medium for 20 h, all the genes for urea cycle were silenced (Fig. 6F). Urea cycle can be active when the excessive amino acids are degraded to produce extra nitrogen. Therefore, the urea cycle was down-regulated as there were no extra amino acids for degradation, especially when the strain was inoculated in the oligotrophic feather medium. ​In particular, all living cells need to synthesize the aromatic amino acids, histidine and intermediates of pyruvate group amino acids as above for growth and metabolism. Interestingly, nitrate reductase (EC1.7.99-) and nitrite reductase (NADH) (EC1.7.1.15) which can convert nitrate to ammonia were up-regulated during the late period (20 h). The ammonia could be attribute to the pH increase in the feather medium. This is consistent with Sivakumar’s research [68].

### Characterization of the keratinolytic proteases

**Fig. 7:**
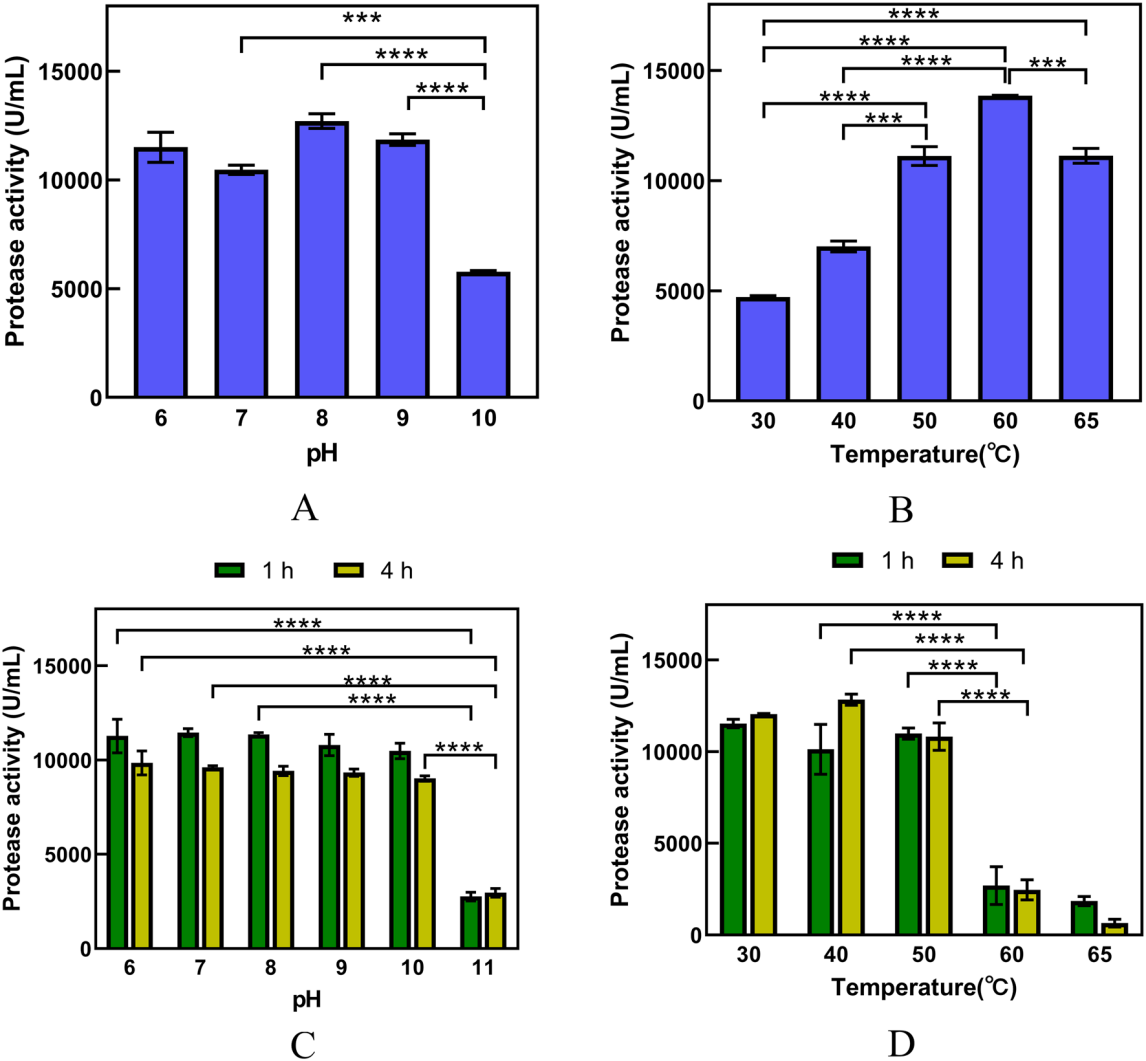
**A** Optimal pH of *Bacillus* sp. 8A6 keratinolytic protease. **B** Optimal temperature of *Bacillus* sp. 8A6 keratinolytic protease. **C** Alkaline stability of *Bacillus* sp. 8A6 keratinolytic protease. **D** Thermostability of *Bacillus* sp. 8A6 keratinolytic protease. The green and yellow mean the *Bacillus* sp. 8A6 keratinolytic protease incubating for 1 h and 4 h, respectively. (*p < 0.05, **p < 0.01, ***p < 0.001, ****p < 0.0001)

So far, the maximum keratinase production by *Bacillus* sp. 8A6 has been easily achieved under the optimized fermentation conditions obtained by single-factor optimization and RSM. Further, the catalytic efficiency of keratinolytic protease was evaluated by measuring its activity at different pH (6.0–11.0) and temperatures (30–65 °C) (Fig. 7A and B). As can be seen from Fig. 7A and B, the optimal pH and temperature for the highest protease activity were separately detected at alkaline pH 8.0 (12,712.5 ± 335.9 U/mL) and at 60 °C (13,862.5 ± 17.7 U/mL), which were similar to other reported keratinolytic proteases [5]. Meanwhile, the acidic/alkaline stability and thermostability were also investigated. Results showed that protease was stable over the pH range of 6.0–10.0 after 4 h of incubation (10,800.0–11,450.0 U/mL) with an exception at pH 11.0. Interestingly, protease lost about 75% of its activity after 1 h of incubation at pH 11.0 but retained the activity after 4 h of incubation (Fig. 7C). In the case of thermostability (Fig. 7D), better stability was observed in the temperature range of 30–50 °C after 1 h of incubation (10,137.5–11,537.5 U/mL), which was retained for 4 h of incubation (10,825.0–12,837.5 U/mL). Thus, keratinolytic protease is active over a wider pH and temperature range, which is highly desirable for industrial applications.

### Keratinases for producing functional peptides and amino acids from the soybean

Keratinases can be used as additives in animal feed to improve digestibility [69]. The commercial keratinase Cibenza DP100™ and Versazyme from *B. licheniformis* PWD-1 have been used in animal feed and significantly improved the crude protein digestibility [70, 71]. In this study, the *Bacillus* sp. 8A6 keratinases were the first attempt to be used for improving soybean protein digestibility. The results showed that efficient keratinase could be applied for soybean protein hydrolysis, resulting in the formation of functional peptides and amino acids. The released functional peptides and amino acids after the degradation by *Bacillus* sp. 8A6 keratinases and well-known soybean degradation commercial protease NY100 were analyzed. During the hydrolysis of soybean protein, *Bacillus* sp. 8A6 keratinases produced 2234 short peptides (Supplemental peptide list 1) with a relative concentration of 1.14 × 10^11^, higher than commercial NY100, which produced 1839 short peptides (Supplemental peptide list 2) at a relative concentration of 8.34 × 10^10^. On the contrary, negative control (CK) produced 108 short peptides (Supplemental peptide list 3) with a relative concentration of 3.49 × 10^9^. Comparatively, the species and concentration of short peptides produced by *Bacillus* sp. 8A6 keratinases and commercial NY100 separately increased nearly 20-fold and 1–2 orders of magnitude compared to CK, proofing the superiority of *Bacillus* sp. 8A6 keratinases. Further analysis of amino acids released during hydrolysis, it was found that 17 functional amino acids, including 6 essential amino acids (lysine, phenylalanine, threonine, isoleucine, leucine, and valine) were produced after 1 h of degradation at 60 °C (Table 2). Among the amino acids, methionine (100.23 mg/L) and phenylalanine (82.81 mg/L) for *Bacillus* sp. 8A6 keratinases, leucine (82.44 mg/L) and methionine (66.05 mg/L) for NY100 were the two most abundant amino acids, with almost twice the amount of abundant amino acids released by CK. More importantly, the most abundant amino acids produced by *Bacillus* sp. 8A6 keratinases and NY100 each contained one essential amino acid, namely phenylalanine and leucine. Meanwhile, the amino acid production of *Bacillus* sp. 8A6 keratinases and NY100 both reached superior levels (559.93 mg/L, 460.83 mg/L) at 1 h of hydrolysis, whereas the amino acid production of CK only reached 105.46 mg/L. In addition, *Bacillus* sp. 8A6 keratinases degrade soybeans to produce more amino acids than papain [72]. This efficient soybean protein degradation by *Bacillus* sp. 8A6 keratinase can not only improve the apparent digestibility of the soybean protein, but also have the potential to reduce or eliminate the antigenicity of soybean protein [27]. Therefore, the complete degradation of soybean protein and the released abundant functional peptides and amino acids from the soybean protein indicated that the keratinases might have the potential for feed application.

**Table 2:** Amino acid content after the degradation of soybean by the NY100, *Bacillus* sp. 8A6 keratinase

Amino acid	Unit	CK	*Bacillus* sp. 8A6 keratinases	Commercial protease NY100
Lysine	mg/L	0.41	34.34	9.28
Phenylalanine	mg/L	0.97	82.81	39.13
Threonine	mg/L	0.41	14.28	26.75
Isoleucine	mg/L	0.28	28.24	42.92
Leucine	mg/L	0.91	56.76	82.44
Valine	mg/L	0.93	33.93	34.64
Methionine	mg/L	55.25	100.23	66.05
Histidine	mg/L	0.94	4.73	12.23
Glutamic acid	mg/L	34.91	43.7	48.67
Alanine	mg/L	1.43	14.51	7.18
Glycine	mg/L	1.08	50.6	4.31
Aspartic acid	mg/L	1.96	16.53	3.42
Cystine	mg/L	− (< 2.18)	− (< 2.18)	− (< 2.18)
Proline	mg/L	1.1	11.21	12.97
Serine	mg/L	0.3	23.67	10.64
Tyrosine	mg/L	0.28	37.5	46.54
Arginine	mg/L	4.3	6.89	14.66
Total amino acid content	mg/L	105.46	559.93	461.83

## Conclusions

**Fig. 8:**
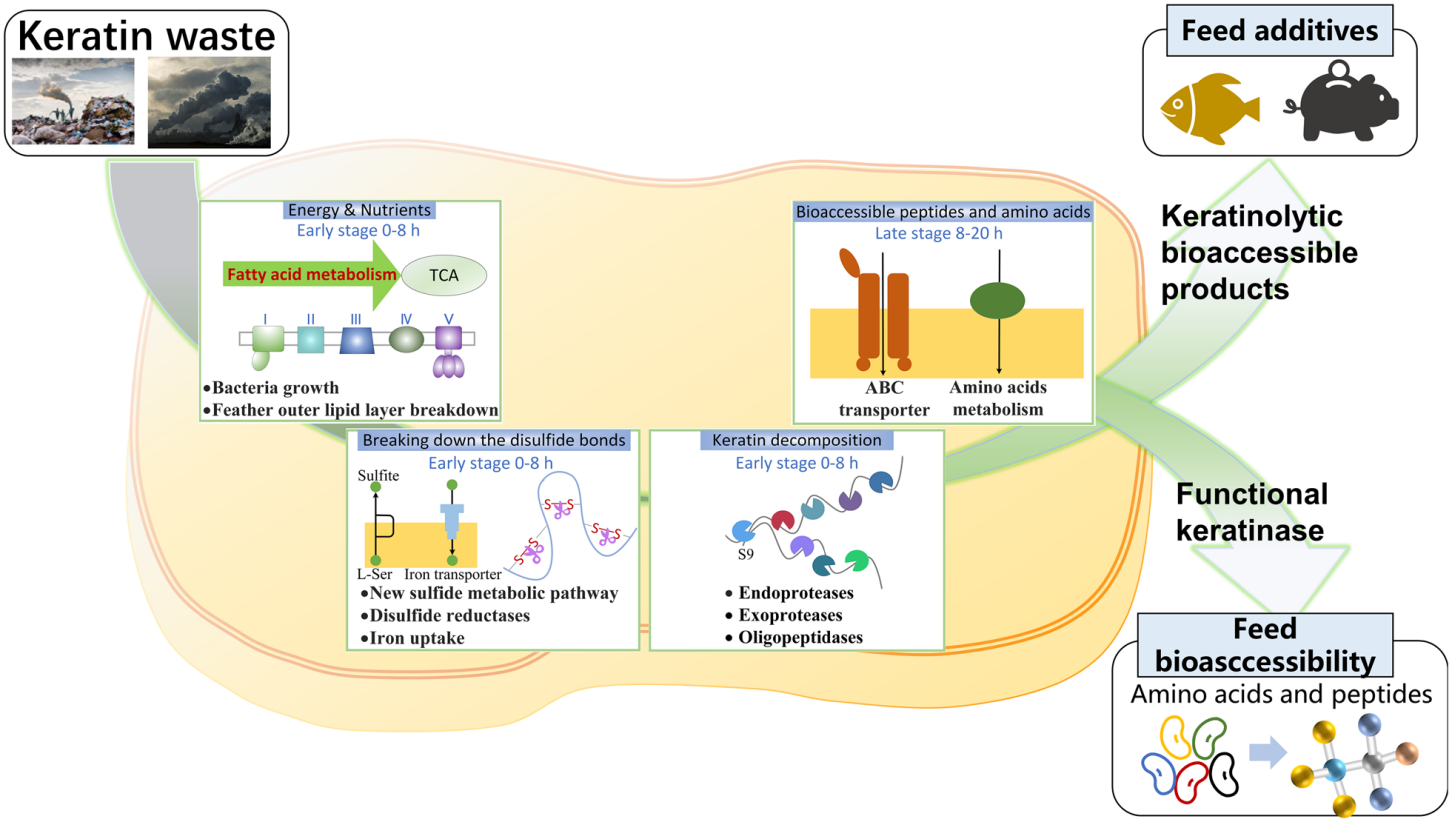
Summary of keratin degradation mechanism, the resulted keratinolytic bioaccessible products and the multi-functional keratinases can be applied in feed industry

In this study, we proposed the feather degradation mechanism as follows according to the comprehensive transcriptome analysis (Fig. 8): (1) fatty acid degradation, TCA cycle and oxidative phosphorylation firstly provide energy and nutrients for strain growth in the oligotrophic feather medium during the early stage; (2) the degraded outer lipid layer of feather can be further destroyed the disulfide bonds by sulfite, disulfide reductases and iron uptake; (3) the resulting loosen keratin was further de-assembled by the S9 proteases and hydrolyzed by synergistic effects of the endo, exo and oligo-proteases from the S1, S8, M3, M14, M20, M24, M42, M84, and T3 families; (4) the generated bioaccessible peptides and amino acids were transported by transporters to the cell for strain growth and metabolism. Finally, the efficient keratinases were used for soybean hydrolysis and generated 2234 short peptides and 559.93 mg/L 17 free amino acids, which were much higher than the commercial protease and negative control. Therefore, the keratinase can be produced during the feather degradation, which has great potential to be used for feed application.

### Supplementary Information

**Additional file 1. Table S1**. Primers used in this study.** Table S2**. Response-surface analysis of the conditions when *Bacillus* sp. 8A6 grew in feather medium.** Table S3**. Quality analysis of the raw RNA sequencing data when *Bacillus* sp. 8A6 grew in feather medium for 0, 8 and 20 h. **Table S4**. Quality analysis of the clean RNA sequencing data when *Bacillus* sp. 8A6 grew in feather medium for 0, 8 and 20 h. **Table S5**. Up-regulated keratinases, disulfide reductases for the group 0 h VS 8 h when *Bacillus* sp. 8A6 grew in feather medium.** Table S6**. Up-regulated keratinases, disulfide reductases for the group 8 h VS 20 h when *Bacillus* sp. 8A6 grew in feather medium.** Figure S1**. Four factors that affect protease activity when the *Bacillus* sp. 8A6 grew in feather medium. (**A**) Effects of different temperatures; (**B**) Effects of different pH; (**C**) Effects of different feather concentration; (**D**) Effects of inoculation amount (*p < 0.05，**p < 0.01, ***p < 0.001, ****p < 0.0001). Response surface 3D plots reveals the interaction of factors affecting protease activity, (**E**) pH and inoculation amount; (**F**) inoculation amount and feather concentration; (**G**) feather concentration and pH.** Figure S2**. The OD_600_ of *Bacillus* sp. 8A6 when grown in feathermedium at different time points.** Figure S3**. Four factors that affect protein concentration. (**A**) Effects of different temperatures; (**B**) Effects of different pH; (**C**) Effects of different feather concentration; (**D**) Effects of inoculation amount (*p < 0.05，**p < 0.01, ***p < 0.001, ****p < 0.0001).** Figure S4**. Pearson correlation coefficient correlation analysis (**A**) and principal component analysis (**B**) among the three RNA-seq samples when *Bacillus* sp. 8A6 grew in feather medium for 0, 8 and 20 h.** Figure S5**. Volcano plot of the differentially expressed genes for the groups 0 h VS 8 h (**A**) and 8 h VS 20 h (**B**) when *Bacillus* sp. 8A6 grew in feather medium. Up-regulated genes are shown as red dots and down-regulated genes are shown as blue dots. The significant difference means that the expression level of the genes are 2 times different and the qvalue (fdr, pad) ≤0.05.** Figure S6**. Hierarchical clusters of differentially expressed genes according to the log10(FPKM+1) value when *Bacillus* sp. 8A6 grew in feather medium for 0, 8 and 20 h. The up-regulated genes were indicated as red color and the down-regulated genes were indicated as blue color based on the color key. Six main clusters were enriched and highlighted by different colors next to the phylogenetic tree.** Figure S7**. Gene Ontology (GO) analysis of the molecular function, cellular component and biological process for the groups 0 h VS 8 h (**A**) and 8 h VS 20 h (**B**) according to the -log10(pvalue) when *Bacillus* sp. 8A6 grew in feather medium. The higher -log10(p-value) indicates the more significant for enrichment of the GO term. Kyoto Encyclopedia of Genes and Genomes (KEGG) analysis of the pathways for the groups 0 h VS 8 h (**C**) and 8 h VS 20 h (**D**) according to the -log10(p-value) when *Bacillus* sp. 8A6 grew in feather medium. 30 significantly different pathways are shown according to the Q-value.** Figure S8**. Cluster of Orthologous Groups of proteins (COG) analysis of the gene function for the groups 0 h VS 8 h (**A**) and 8 h VS 20 h (**B**) according to the gene numbers when *Bacillus* sp. 8A6 grew in feather medium. The up-regulated genes were indicated as red color and the down-regulated genes were indicated as blue color.** Supplemental peptide list 1**. Short peptides of *Bacillus* sp. 8A6 keratinases.** Supplemental peptide list 2**. Short peptides of NY100. **Supplemental peptide list 3**. Short peptides of CK.

## Data Availability

The datasets supporting the conclusions of this article are included within the article and its additional files.

## Abbreviations

RT-qPCR: Real-time fluorescence quantitative PCR
DEGs: Differentially expressed genes
RSM: Response surface method
GO: Gene Ontology
KEGG: Kyoto Encyclopedia of Genes and Genomes
COG: Cluster of Orthologous Groups of proteins
SEM: Scanning electron microscopy
SPI: Soy protein isolate
SD: Standard deviation
Ubls: Ubiquitin-like proteins
CK: Negative control
