# Supplementary material for: Comprehensive insights into the mechanism of keratin degradation and exploitation of keratinase to enhance the bioaccessibility of soybean protein
Source: Biotechnol Biofuels Bioprod. 2023 Nov 17;16:177. doi: 10.1186/s13068-023-02426-9 (PMC10655438; doi:10.1186/s13068-023-02426-9)
Supplement: Supplementary file 1 — Additional file 1. Table S1. Primers used in this study. Table S2. Response-surface analysis of the conditions when Bacillus sp. 8A6 grew in feather medium. Table S3. Quality analysis of the raw RNA sequencing data when Bacillus sp. 8A6 grew in feather medium for 0, 8 and 20 h. Table S4. Quality analysis of the clean RNA sequencing data when Bacillus sp. 8A6 grew in feather medium for 0, 8 and 20 h. Table S5. Up-regulated keratinases, disulfide reductases for the group 0 h VS 8 h when Bacillus sp. 8A6 grew in feather medium. Table S6. Up-regulated keratinases, disulfide reductases for the group 8 h VS 20 h when Bacillus sp. 8A6 grew in feather medium. Figure S1. Four factors that affect protease activity when the Bacillus sp. 8A6 grew in feather medium. (A) Effects of different temperatures; (B) Effects of different pH; (C) Effects of different feather concentration; (D) Effects of inoculation amount (*p < 0.05，**p < 0.01, ***p < 0.001, ****p < 0.0001). Response surface 3D plots reveals the interaction of factors affecting protease activity, (E) pH and inoculation amount; (F) inoculation amount and feather concentration; (G) feather concentration and pH. Figure S2. The OD600 of Bacillus sp. 8A6 when grown in feathermedium at different time points. Figure S3. Four factors that affect protein concentration. (A) Effects of different temperatures; (B) Effects of different pH; (C) Effects of different feather concentration; (D) Effects of inoculation amount (*p < 0.05，**p < 0.01, ***p < 0.001, ****p < 0.0001). Figure S4. Pearson correlation coefficient correlation analysis (A) and principal component analysis (B) among the three RNA-seq samples when Bacillus sp. 8A6 grew in feather medium for 0, 8 and 20 h. Figure S5. Volcano plot of the differentially expressed genes for the groups 0 h VS 8 h (A) and 8 h VS 20 h (B) when Bacillus sp. 8A6 grew in feather medium. Up-regulated genes are shown as red dots and down-regulated genes are shown as blue dots. The significant diff [file 13068_2023_2426_MOESM1_ESM.docx]

**Supporting Information**

**Comprehensive insights into the mechanism of keratin degradation and exploitation of** **keratinase to enhance the bioaccessibility of soybean protein**

Beiya Zhou^1, 2, 3^, Yandong Guo^1^*, Yaju Xue^2^, Xiuling Ji^2^, Yuhong Huang^2^*

^1^ College of Mathematical Sciences, Bohai University, Jinzhou, Liaoning 121013, China

^2^ Beijing Key Laboratory of Ionic Liquids Clean Process, CAS Key Laboratory of Green Process and Engineering, State Key Laboratory of Multiphase Complex Systems, Institute of Process Engineering, Chinese Academy of Sciences, Beijing 100190, China

^3^ Huizhou Institute of Green Energy and Advanced Materials, Huizhou, Guangdong, 516000, China

*Corresponding author:

Yandong Guo, gyd1030@163.com

Yuhong Huang, [yhhuang@ipe.ac.cn](mailto:yhhuang@ipe.ac.cn)

1. **Supplemental Materials and Method**
   1. **Single-factor experiment**

In this study, all factors except one factor remained at a constant level. The single-factor experiment contained the effect of temperature of cultivated (25, 37, 40, 45 ℃), pH of feather medium (5, 6, 7, 8, 9, 10), the concentration of feathers (0.5%, 1%, 2%, 3%, 4%, 5%, 10% (w/v)) and inoculation volume of seed culture (1 mL, 2 mL, 3 mL) in flask fermentation of degraded feathers.

*Bacillus* sp. 8A6 was revitalized twice by 5 mL LB medium at 37 ℃ at 200 rpm for 20 h, inoculated 200 μL above culture to 50 mL LB medium at 37 ℃, 200 rpm for 12 h with the final concentration of bacterium attaining to OD_600_ 4-6 to obtain seed culture. Then seed culture was pretreated by centrifugation at 6000 rpm for 10 min at 4℃ and resuspension with 25 mL sterile PBS buffer (50 mM, pH 7.5). After adding different inoculation volume (1, 2, and 3 mL) resuspension liquid into the sterile feather medium of various pH (5, 6, 7, 8, 9 and 10) and concentration of feathers (0.5, 1, 2, 3, 4, 5, and 10% (w/v)), flask fermentation was cultivated at different temperature (25, 37, 40, and 45 ℃) for 20 h with shaking at 200 rpm. Finally, the supernatant and keratin were collected after being centrifuged at 4 ℃ for 20 min at 12000 rpm.

**1.2 Response-surface analysis**

According to the result of single-factor experiment, the three significant independent variables were used to confirm their optimum levels by using RSM, including inoculation volume (A), pH (B) and feather concentration (C). Based on the Box-Behnken method to measure the optimal value of three factors, 17 test sites with 3 factors and 3 levels were designed (including 5 replicates at the intermediate points) to fit the quadratic response surface. The design model and corresponding experimental data were described in Table S2. The final response value of each group experiment was taken as the average value of two parallel tests. The following quadratic polynomial equation was used to describe the mathematical relationships between the response (protease activity) and the variables:

$$Y=b_{0}-b_{1}A+b_{2}B-b_{3}C-b_{4}AB-b_{5}AC+b_{6}BC-b_{7}A^{2}-b_{8}B^{2}-b_{9}C^{2}$$

where, Y is response value (protease activity, U/mL), A is inoculation volume (mL), B is pH and C is feather concentration (%, w/v), they are input variables which affect predicted response Y, *b*_0_ is constant term, *b*_1_*, b*_2_*, b*_3_ are linear coefficients, *b*_4_, *b*_5_, *b*_6_ are cross-item coefficients, *b*_7_, *b*_8_, *b*_9_ are quadratic coefficients.

Design-Expert 12.0 was used to analyse the variance of obtained data to conclude whether the differences between the value of single-factor experiment and Box-Behnken experiment are significant or not.

1. **Supplemental Results and Discussion**

**2.1 Single factor optimization of keratinase production conditions**

In this study, single factor optimization and response surface methodology (RSM) were applied to find out the optimal fermentation conditions which were required for maximum keratinase production by the efficient keratin degrader *Bacillus* sp. 8A6 grown in feather medium. During single factor optimization, four independent factors, namely incubation temperature (25−45 °C), pH (5.0−10.0), feather concentration (0.5−10.0% w/v), and inoculation volume of seed culture were evaluated for their effects on protease activity and protein concentration (Fig S1 and S2). In the case of temperature effect, the highest protease activity (11362.5 U/mL) and protein concentration (1393.217 μg/mL) when the strain grew under 37 °C and 45 °C, respectively (Figs S1A and S2A). This is consistent with the results that most of the reported *Bacillus* sp. produce maximum keratinase in the temperature range of 30−50 °C [1-4]. To further optimize pH for keratinase production, *Bacillus* sp. 8A6 was cultivated with feather medium at different pH values, which exhibited 9.0 as optimum pH for the maximum protease activity (12700.0 U/mL) and protein concentration (2888.05 μg/mL) (Figs S1B and S2B). *Bacillus* sp. 8A6 is a basophilic granulocyte, thus the fermentation at alkaline pH usually achieves the enhanced enzyme activity. Since feather as the carbon and nitrogen source for fermentation, feather concentration is another prime determinant for the growth of *Bacillus* sp. 8A6 and associated keratinase production. Feather concentration at 5% (w/v) was found to be optimal for *Bacillus* sp. 8A6 producing a protease activity of 15437.5 U/mL and a protein concentration of 10434 μg/mL , the cause of which has been reported to be an increased viscosity of the medium followed by inadequate aeration (Figs S1C and S2C) [5]. The inoculation volume of seed medium added also affects protease activity and protein concentration, protease activity (12700 U/mL) and protein concentration (9389.176 μg/mL) were highest when inoculated at 2 mL (Figs S1D and S2D). After single factor optimization, add trace elements (0.2500 g/L MgSO_4_·7H_2_O, 0.0550 g/L CaCl_2_, 0.0100 g/L FeSO_4_·7H_2_O, 0.0050 g/L ZnSO_4_·7H_2_O) to the optimized fermentation conditions after 20 h of fermentation at 37 °C can obtain higher protease activity with 5% (w/v) feather concentration at pH 9.0.

**2.2 RSM optimization of keratinase production conditions**

Based on the results of single factor optimization, three independent factors, namely feather concentration, pH, and inoculation amount were further investigated to assess their synergistic effects on *Bacillus* sp. 8A6 to enhance keratinase production by RSM optimization. During RSM optimization, other factors including incubation temperature (37 °C), feather concentration (5 %), and inoculation amount (2 mL) were fixed at optimized levels obtained by single factor optimization along with protease activity. The regression analysis of the experimental data resulted in the following second-order polynomial equation for protease activity (Y):

$$Y_{protease activity (U/mL)}=11665-420.31\times A+385.94\times B-1146.88\times C -259.38\times AB-450\times AC+18.75\times BC-893.44\times A^{2}-1005.94\times B^{2}-1459.06\times C^{2}$$

Where A, B, and C represented inoculation amount, pH, and feather concentration, respectively. Analysis of variance (ANOVA) for response surface quadratic model revealed that F value of 8.79 and *p* value of 0.0045 demonstrated the significance of model, indicative of the dependence of protease activity on the interplay of inoculation amount, pH, and feather concentration (Table S2). In addition, three-dimensional response surfaces and contour plots for protease activity were employed to evaluate the quadratic model, providing an intuitive understanding of the interaction between two factors at a time with another factor locate being kept constant to achieve the optimum protease activity. Fig S1 exhibited three significant mutual interactions. As can be seen, the predictive fermentation conditions for the optimal protease activity of 11951.46 U/mL were inoculation amount of 1.83 mL and feather concentration of 3.905% (w/v) at pH 9.21. Validation of the same protease activity obtained experimentally showed close fermentation conditions with an inoculation amount of 1.83 mL and a feather concentration of 3.905% (w/v) at pH 9.21, which in turn indicated the reliability of the model. This growth conditions will support future scale up keratinase production as described in fermenters [6, 7].

**Table S1 Primers used in this study**

| **Gene** | **Family** | **Primer name** | **Primer sequences（5’-3’）** |
| --- | --- | --- | --- |
| 16sRNA | - | Forward | CACACTGGGACTGAGACACG |
|  |  | Reverse | CCGTGGCTTTCTCGTTAGGT |
| RS15255 | S8 | Forward | AAAGGACCTGCCCCAAAAG |
|  |  | Reverse | AGCGACGCATACAACAATG |
| RS16285 | S8 | Forward | GGAACCCCTCATCCAGAAA |
|  |  | Reverse | GCAAAGAGAAAGAACCCAGT |
| RS11220 | S9 | Forward | ACAAAGGACAGCCAAGG |
|  |  | Reverse | GTTCAGGTTCCGATACGATA |
| RS14955 | S1A | Forward | TTGCCGTGATTAAAACCGATACG |
|  |  | Reverse | TCTCCCATTGTGACACCTTGC |
| RS08615 | S8 | Forward | AAGATTCCCTACTGCTGCCTCC |
|  |  | Reverse | CGTGGGTAACCTGCCTGTAAG |
| RS13920 | S8 | Forward | TAGTCGTCGTGGCAAGTGGAA |
|  |  | Reverse | AATATCTGACCCTGGTGCTGAC |
| RS17315 | S8 | Forward | AACTATGCCGCAGAGCAGAAGG |
|  |  | Reverse | AGCAAGGGAAACAGAACCGAC |

**Table S2 Response-surface analysis of the conditions when *Bacillus* sp. 8A6 grew in feather medium**

| **Number** | **Inoculation amount**  **(mL)** | **pH** | **Feather concentration (%, w/v)** | **Protease activity**  **(U/mL)** |
| --- | --- | --- | --- | --- |
| 1 | 1 | 8 | 5 | 9012.5 |
| 2 | 3 | 8 | 5 | 8875 |
| 3 | 1 | 10 | 5 | 11175 |
| 4 | 3 | 10 | 5 | 10000 |
| 5 | 1 | 9 | 2 | 10450 |
| 6 | 3 | 9 | 2 | 10325 |
| 7 | 1 | 9 | 8 | 9200 |
| 8 | 3 | 9 | 8 | 7275 |
| 9 | 2 | 8 | 2 | 10487.5 |
| 10 | 2 | 10 | 2 | 10350 |
| 11 | 2 | 8 | 8 | 8012.5 |
| 12 | 2 | 10 | 8 | 7950 |
| 13 | 2 | 9 | 5 | 12000 |
| 14 | 2 | 9 | 5 | 11875 |
| 15 | 2 | 9 | 5 | 12125 |
| 16 | 2 | 9 | 5 | 10725 |
| 17 | 2 | 9 | 5 | 11600 |

**Table S3** Quality analysis of the raw RNA sequencing data when *Bacillus* sp. 8A6 grew in feather medium for 0, 8 and 20 h.

| **Sample** | **Length** | **Reads** | **Bases** | **Q20(%)** | **Q30(%)** | **GC(%)** | **N(ppm)** |
| --- | --- | --- | --- | --- | --- | --- | --- |
| 0h | 150 | 30511676 | 4576751400 | 98.07 | 94.96 | 44.73 | 33.09 |
| 8h | 150 | 34580052 | 5187007800 | 97.85 | 94.13 | 45.15 | 13.96 |
| 20h | 150 | 31894300 | 4784145000 | 96.95 | 93.45 | 47.12 | 27.41 |

**Table S4** Quality analysis of the clean RNA sequencing data when *Bacillus* sp. 8A6 grew in feather medium for 0, 8 and 20 h.

| **Sample** | **Length** | **Reads** | **Bases** | **Q20(%)** | **Q30(%)** | **GC(%)** | **N(ppm)** | **Reads(%)** | **Bases(%)** |
| --- | --- | --- | --- | --- | --- | --- | --- | --- | --- |
| 0h | 147.56 | 30126818 | 4445659838 | 98.47 | 95.44 | 44.44 | 25.21 | 98.74 | 97.14 |
| 8h | 147.28 | 34376632 | 5062950398 | 98.10 | 94.44 | 45.02 | 9.15 | 99.41 | 97.61 |
| 20h | 137.71 | 30277688 | 4169413173 | 98.19 | 95.06 | 45.07 | 21.21 | 94.93 | 87.15 |

**Table S5** up-regulated keratinases, disulfide reductases for the group 0 h VS 8 h when *Bacillus* sp. 8A6 grew in feather medium

| **geneID** | **family** | **0h.FPKM** | **8h.FPKM** | **logFC** | **logCPM** | **pval** | **FDR** | **Regulation** | **Product** |
| --- | --- | --- | --- | --- | --- | --- | --- | --- | --- |
| gene-DJ474_RS11160 | M23B | 0 | 6.61 | 9.328259 | 1.630352 | 1.32E-07 | 1.14E-06 | Ups | peptidoglycan DD-metalloendopeptidase family protein |
| gene-DJ474_RS00685 | M78 | 0 | 12.44 | 9.240045 | 1.550022 | 2.03E-07 | 1.65E-06 | Ups | ImmA/IrrE family metallo-endopeptidase |
| gene-DJ474_RS05440 | C82A | 0 | 9.44 | 8.860733 | 1.207634 | 1.63E-06 | 1.06E-05 | Ups | L%2CD-transpeptidase |
| gene-DJ474_RS08645 | s9 | 0.28 | 106.41 | 7.960807 | 5.571195 | 2.56E-16 | 1.46E-14 | Ups | chitobiase/beta-hexosaminidase C-terminal domain-containing protein |
| gene-DJ474_RS00375 | M15 | 0 | 1.28 | 6.681922 | -0.62847 | 0.002864 | 0.008289 | Ups | M15 family metallopeptidase |
| gene-DJ474_RS05335 | - | 0.77 | 103.68 | 6.496456 | 5.792405 | 8.55E-14 | 2.78E-12 | Ups | SpoIVB peptidase |
| gene-DJ474_RS00915 | M14A | 15.43 | 1635.26 | 6.155057 | 10.11661 | 4.85E-14 | 1.72E-12 | Ups | peptidase M14 |
| gene-DJ474_RS11940 | M78 | 0 | 1.47 | 6.147726 | -1.0246 | 0.014079 | 0.03283 | Ups | ImmA/IrrE family metallo-endopeptidase |
| gene-DJ474_RS08920 | M84 | 172.86 | 11731.06 | 5.512325 | 11.96472 | 3.21E-12 | 7.24E-11 | Ups | zinc-dependent metallokeratinase |
| gene-DJ474_RS01795 | U57 | 0.34 | 17.37 | 5.067613 | 2.786876 | 3.95E-08 | 3.86E-07 | Ups | sporulation peptidase YabG |
| gene-DJ474_RS18405 | M56 | 7.36 | 284.91 | 4.700925 | 6.677889 | 1.11E-09 | 1.47E-08 | Ups | M56 family metallopeptidase |
| gene-DJ474_RS05690 | S8 | 3.57 | 133.11 | 4.645668 | 5.97264 | 2.13E-09 | 2.67E-08 | Ups | S8 family peptidase |
| gene-DJ474_RS09920 | s41 | 1.25 | 40.89 | 4.455787 | 4.692552 | 1.46E-08 | 1.56E-07 | Ups | PDZ domain-containing protein |
| gene-DJ474_RS15255 | S8 | 0.45 | 14.08 | 4.364333 | 3.077196 | 2.18E-07 | 1.76E-06 | Ups | S8 family peptidase |
| gene-DJ474_RS02400 | S9 | 2.67 | 75.34 | 4.241361 | 4.898353 | 4.35E-08 | 4.22E-07 | Ups | acetylxylan esterase |
| gene-DJ474_RS16285 | S8 | 7.28 | 201.55 | 4.217995 | 8.556213 | 1.57E-08 | 1.68E-07 | Ups | S8 family serine peptidase |
| gene-DJ474_RS11220 | S9 | 10.94 | 295.68 | 4.183694 | 7.980661 | 2.07E-08 | 2.15E-07 | Ups | S9 family peptidase |
| gene-DJ474_RS17430 | c40 | 19.63 | 498.69 | 4.094393 | 7.637935 | 3.65E-08 | 3.58E-07 | Ups | C40 family peptidase |
| gene-DJ474_RS15425 | S14 | 3 | 74.67 | 4.061426 | 4.591098 | 1.44E-07 | 1.23E-06 | Ups | ClpP family keratinase |
| gene-DJ474_RS05725 | S11 | 3.43 | 79.98 | 3.970133 | 5.388862 | 1.32E-07 | 1.14E-06 | Ups | D-alanyl-D-alanine carboxypeptidase |
| gene-DJ474_RS14180 | G5 | 4.96 | 108.16 | 3.874531 | 6.151593 | 1.84E-07 | 1.52E-06 | Ups | CPBP family intramembrane metallokeratinase |
| gene-DJ474_RS04675 | A25 | 1.89 | 36.24 | 3.683476 | 4.193957 | 1.25E-06 | 8.34E-06 | Ups | GPR endopeptidase |
| gene-DJ474_RS14955 | S1A | 115.54 | 2194.19 | 3.674965 | 9.785222 | 4.15E-07 | 3.14E-06 | Ups | trypsin-like serine keratinase |
| gene-DJ474_RS17440 | S66 | 46.98 | 880.92 | 3.656442 | 8.522446 | 4.87E-07 | 3.65E-06 | Ups | LD-carboxypeptidase |
| gene-DJ474_RS18510 | S8 | 1307.62 | 24235.54 | 3.639911 | 13.59426 | 5.04E-07 | 3.75E-06 | Ups | S8 family serine peptidase |
| gene-DJ474_RS08615 | S8 | 111.18 | 2030.27 | 3.618464 | 11.09376 | 5.76E-07 | 4.23E-06 | Ups | S8 family serine peptidase |
| gene-DJ474_RS08940 | M20F | 1.46 | 23.46 | 3.434088 | 4.471086 | 4.00E-06 | 2.36E-05 | Ups | PBP1A family penicillin-binding protein |
| gene-DJ474_RS13920 | S8 | 29.03 | 390.09 | 3.175767 | 8.88954 | 7.78E-06 | 4.28E-05 | Ups | S8 family serine peptidase |
| gene-DJ474_RS01465 | S8 | 10.7 | 143.04 | 3.167469 | 5.931805 | 1.04E-05 | 5.54E-05 | Ups | S8 family serine peptidase |
| gene-DJ474_RS09290 | M23B | 71.28 | 918.49 | 3.115446 | 8.559395 | 1.10E-05 | 5.83E-05 | Ups | peptidoglycan DD-metalloendopeptidase family protein |
| gene-DJ474_RS08570 | M24B | 38.01 | 475.42 | 3.072323 | 7.886668 | 1.46E-05 | 7.55E-05 | Ups | aminopeptidase P family N-terminal domain-containing protein |
| gene-DJ474_RS16280 | A36 | 7.26 | 78.63 | 2.862677 | 5.066982 | 6.16E-05 | 0.000277 | Ups | sigma-E processing peptidase SpoIIGA |
| gene-DJ474_RS17960 | M3B | 24.47 | 249.92 | 2.779817 | 7.92112 | 7.11E-05 | 0.000314 | Ups | oligoendopeptidase F |
| gene-DJ474_RS17575 | T3 | 225.23 | 2255.44 | 2.751693 | 10.85967 | 7.90E-05 | 0.000343 | Ups | gamma-glutamyltransferase |
| gene-DJ474_RS07080 | S13 | 47.95 | 459.32 | 2.687732 | 8.317183 | 0.000113 | 0.000479 | Ups | D-alanyl-D-alanine carboxypeptidase/D-alanyl-D-alanine-endopeptidase |
| gene-DJ474_RS05860 | S11 | 1.18 | 10.54 | 2.582947 | 2.620245 | 0.000709 | 0.002467 | Ups | D-alanyl-D-alanine carboxypeptidase |
| gene-DJ474_RS14170 | G5 | 12.16 | 101.46 | 2.487975 | 6.103789 | 0.00035 | 0.001313 | Ups | sporulation killing factor system integral membrane protein |
| gene-DJ474_RS03115 | M42 | 48.67 | 355.92 | 2.298078 | 7.582213 | 0.000815 | 0.002793 | Ups | M42 family metallopeptidase |
| gene-DJ474_RS17315 | S8 | 210 | 1480.19 | 2.245055 | 9.463482 | 0.001027 | 0.003393 | Ups | S8 family peptidase |
| gene-DJ474_RS17115 | - | 3.41 | 23.5 | 2.213346 | 4.643648 | 0.001586 | 0.004941 | Ups | ATP-dependent Clp keratinase ATP-binding subunit |
| gene-DJ474_RS05915 | M23B | 0.93 | 6.11 | 2.129543 | 1.173278 | 0.012795 | 0.030407 | Ups | peptidoglycan DD-metalloendopeptidase family protein |
| gene-DJ474_RS19020 | C60A | 224.32 | 1410.7 | 2.080518 | 8.664998 | 0.002224 | 0.006671 | Ups | class D sortase |
| gene-DJ474_RS08135 | M20B | 33.45 | 204.91 | 2.042652 | 7.012459 | 0.002737 | 0.008017 | Ups | peptidase T |
| gene-DJ474_RS02515 | M20F | 13.99 | 82.99 | 1.995728 | 5.906474 | 0.003541 | 0.010009 | Ups | dipeptidase PepV |
| gene-DJ474_RS10080 | C39 | 1.6 | 8.47 | 1.829405 | 1.862532 | 0.017116 | 0.038811 | Ups | C39 family peptidase |
| gene-DJ474_RS03440 | S16 | 14.97 | 77.62 | 1.801824 | 6.096066 | 0.007939 | 0.020125 | Ups | ATP-dependent keratinase LonB |
| gene-DJ474_RS18185 | I22 | 40.42 | 207.85 | 1.789738 | 5.31797 | 0.008646 | 0.021723 | Ups | proteinase inhibitor |
| gene-DJ474_RS13215 | M20D | 2.62 | 12.53 | 1.682433 | 2.973316 | 0.018245 | 0.041018 | Ups | amidohydrolase |
| gene-DJ474_RS05045 | M14A | 15.28 | 72.55 | 1.67482 | 5.468477 | 0.013637 | 0.03209 | Ups | hypothetical protein |
| gene-DJ474_RS12470 | S66 | 8.57 | 40.37 | 1.662116 | 4.494048 | 0.015633 | 0.035912 | Ups | LD-carboxypeptidase |
| gene-DJ474_RS07315 | A25 | 10.1 | 47.04 | 1.646214 | 4.023942 | 0.017208 | 0.038975 | Ups | spore keratinase YyaC |
| gene-DJ474_RS09515 | C40 | 45.27 | 196.88 | 1.548233 | 7.054396 | 0.020971 | 0.04599 | Ups | C40 family peptidase |
| gene-DJ474_RS14780 | - | 0 | 2.86 | 7.629303 | 0.136154 | 9.64E-05 | 0.000413 | Ups | DsbA family protein |
| gene-DJ474_RS17050 | - | 0.15 | 5.67 | 4.505385 | 0.545816 | 0.000336 | 0.001267 | Ups | TlpA family protein disulfide reductase |
| gene-DJ474_RS12725 | - | 1.31 | 36.1 | 4.203999 | 3.972265 | 1.19E-07 | 1.04E-06 | Ups | thiamine pyrophosphate-dependent dehydrogenase E1 component subunit alpha |
| gene-DJ474_RS14160 | - | 4.42 | 119.27 | 4.1753 | 4.487443 | 7.25E-08 | 6.64E-07 | Ups | TlpA family protein disulfide reductase |
| gene-DJ474_RS05595 | - | 31.09 | 295.07 | 2.67422 | 7.542271 | 0.000125 | 0.000525 | Ups | CoA-disulfide reductase |
| gene-DJ474_RS14855 | - | 8.83 | 42.68 | 1.7 | 3.56592 | 0.015876 | 0.0363 | Ups | TlpA family protein disulfide reductase |
| gene-DJ474_RS19160 | - | 0.13 | 1.39 | 2.764554 | -0.05215 | 0.018258 | 0.041018 | Ups | FMNH2-dependent alkanesulfonate monooxygenase |
| gene-DJ474_RS18715 | - | 56.94 | 437.36 | 2.368885 | 8.09444 | 0.000573 | 0.002024 | Ups | SidA/IucD/PvdA family monooxygenase |

**Table S6** up-regulated keratinases, disulfide reductases for the group 8 h VS 20 h when *Bacillus* sp. 8A6 grew in feather medium

| **geneID** | **family** | **8h.FPKM** | **20h.FPKM** | **logFC** | **logCPM** | **pval** | **FDR** | **Regulation** | **Product** |
| --- | --- | --- | --- | --- | --- | --- | --- | --- | --- |
| gene-DJ474_RS15255 | S8 | 14.08 | 1626.61 | 7.017031 | 10.16871 | 1.48E-16 | 8.17E-14 | Ups | S8 family peptidase |
| gene-DJ474_RS00795 | C56 | 23.5 | 354.16 | 4.079302 | 8.706404 | 3.68E-08 | 1.37E-06 | Ups | ATP-dependent Clp keratinase ATP-binding subunit |
| gene-DJ474_RS00865 | M24 | 45.16 | 542.78 | 3.752808 | 7.582742 | 2.85E-07 | 8.41E-06 | Ups | repressor LexA |
| gene-DJ474_RS08040 | C56 | 14.29 | 100.45 | 2.978032 | 5.637809 | 2.85E-05 | 0.000396 | Ups | C40 family peptidase |
| gene-DJ474_RS08360 | C60A | 0.97 | 5.69 | 2.706217 | 1.126316 | 0.001267 | 0.008774 | Ups | class F sortase |
| gene-DJ474_RS08930 | M50B | 207.85 | 1180.54 | 2.671627 | 8.024497 | 0.000123 | 0.001326 | Ups | proteinase inhibitor |
| gene-DJ474_RS12065 | - | 12.64 | 66.25 | 2.554577 | 4.766236 | 0.000297 | 0.00277 | Ups | site-2 keratinase family protein |
| gene-DJ474_RS12600 | S8 | 23.4 | 111.93 | 2.423202 | 5.720862 | 0.000482 | 0.004048 | Ups | type I methionyl aminopeptidase |
| gene-DJ474_RS14930 | S24 | 0.76 | 3.44 | 2.33527 | 0.830515 | 0.006131 | 0.030039 | Ups | S8 family serine peptidase |
| gene-DJ474_RS16730 | S26A | 1480.19 | 5885.11 | 2.15724 | 11.83876 | 0.001543 | 0.010282 | Ups | S8 family peptidase |
| gene-DJ474_RS17115 | - | 452.25 | 1784.27 | 2.146088 | 11.46248 | 0.001626 | 0.010741 | Ups | ATP-dependent keratinase ATP-binding subunit ClpC |
| gene-DJ474_RS17315 | S8 | 6.37 | 22.61 | 1.990648 | 3.045598 | 0.005343 | 0.027092 | Ups | ImmA/IrrE family metallo-endopeptidase |
| gene-DJ474_RS17615 | M78 | 2166.69 | 6820.06 | 1.820243 | 11.23208 | 0.006895 | 0.032851 | Ups | type 1 glutamine amidotransferase |
| gene-DJ474_RS18185 | I22 | 170.13 | 518.13 | 1.772508 | 7.864622 | 0.008521 | 0.038553 | Ups | DJ-1/PfpI family protein |
| gene-DJ474_RS18875 | C40 | 33.54 | 100.98 | 1.755671 | 5.393295 | 0.009714 | 0.042246 | Ups | signal peptidase I |
| gene-DJ474_RS14050 | - | 5.14 | 547.36 | 6.888691 | 6.075854 | 3.79E-15 | 1.07E-12 | Ups | sulfurtransferase TusA family protein |
| gene-DJ474_RS11655 | - | 75.9 | 673.05 | 3.314431 | 8.661032 | 3.50E-06 | 7.24E-05 | Ups | nitronate monooxygenase |


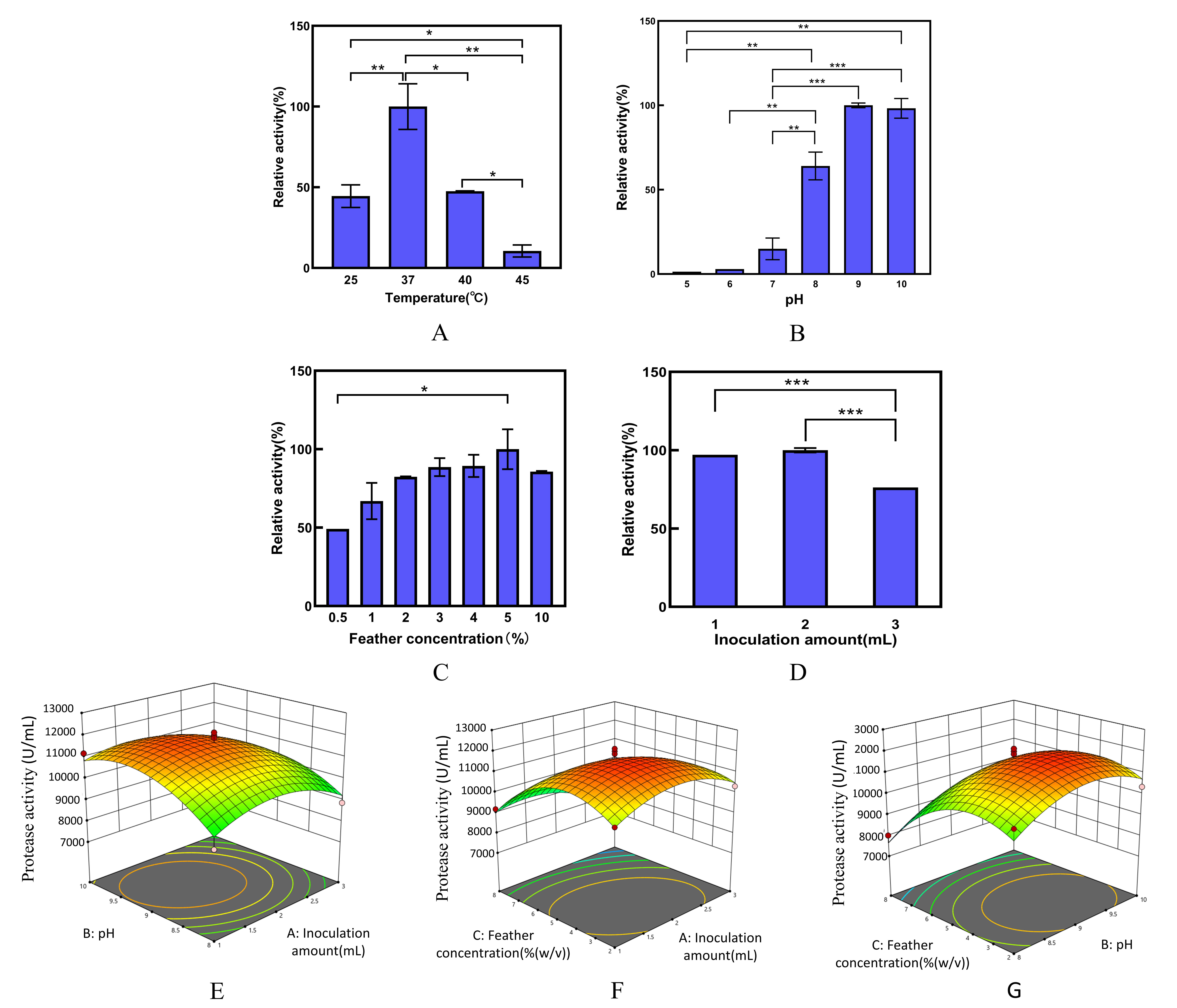


**Fig S1.** Four factors that affect protease activity when the *Bacillus* sp. 8A6 grew in feather medium. (A) Effects of different temperatures; (B) Effects of different pH; (C) Effects of different feather concentration; (D) Effects of inoculation amount (*p < 0.05，**p < 0.01, ***p < 0.001, ****p < 0.0001). Response surface 3D plots reveals the interaction of factors affecting protease activity, (E) pH and inoculation amount; (F) inoculation amount and feather concentration; (G) feather concentration and pH.


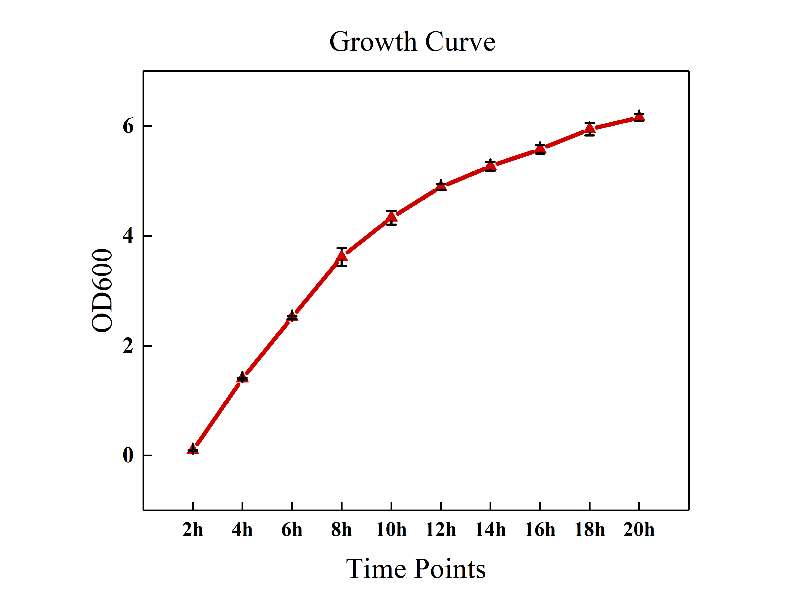


**Fig S2.** The OD_600_ of *Bacillus* sp. 8A6 when grown in feather medium at different time points


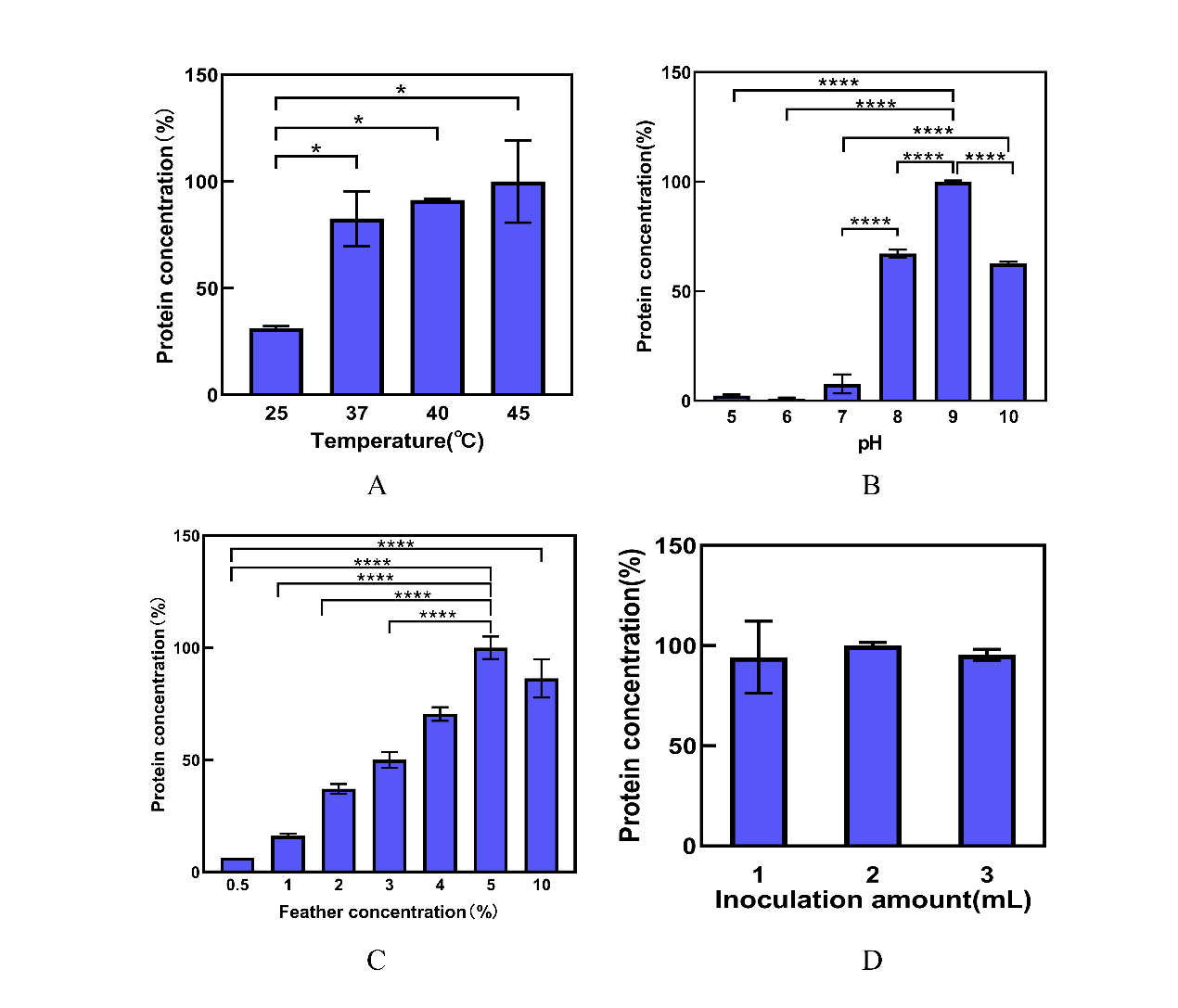


**Fig S3.** Four factors that affect protein concentration. (A) Effects of different temperatures; (B) Effects of different pH; (C) Effects of different feather concentration; (D) Effects of inoculation amount (*p < 0.05，**p < 0.01, ***p < 0.001, ****p < 0.0001)


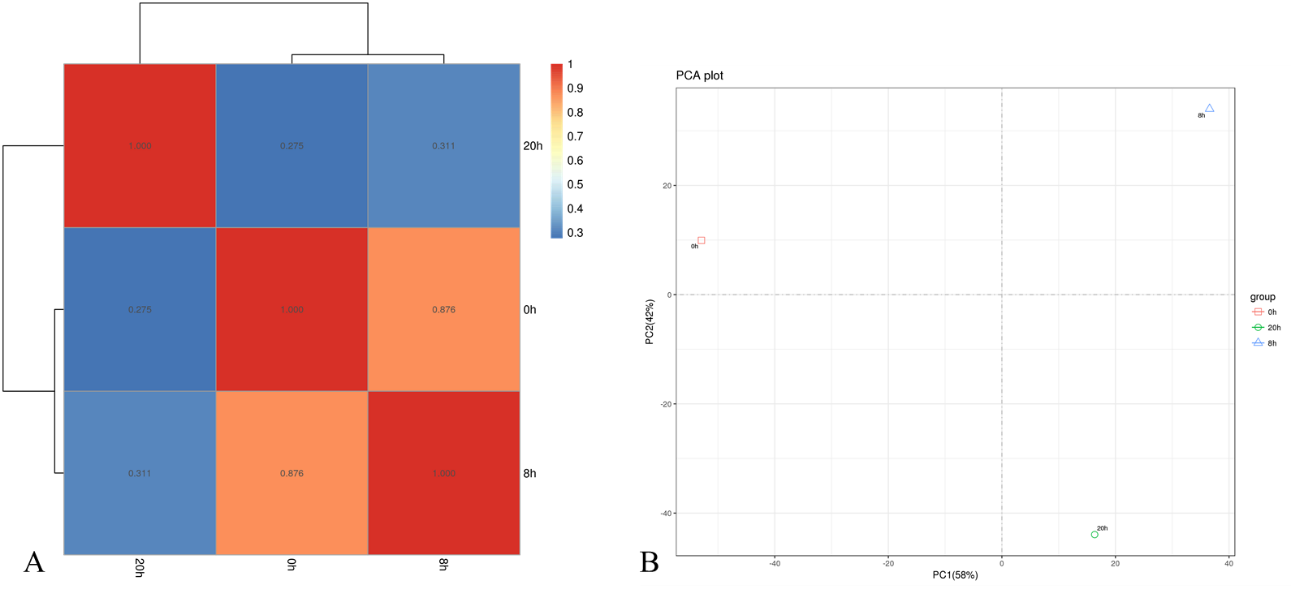


**Fig S4.** Pearson correlation coefficient correlation analysis (A) and principal component analysis (B) among the three RNA-seq samples when *Bacillus* sp. 8A6 grew in feather medium for 0, 8 and 20 h.


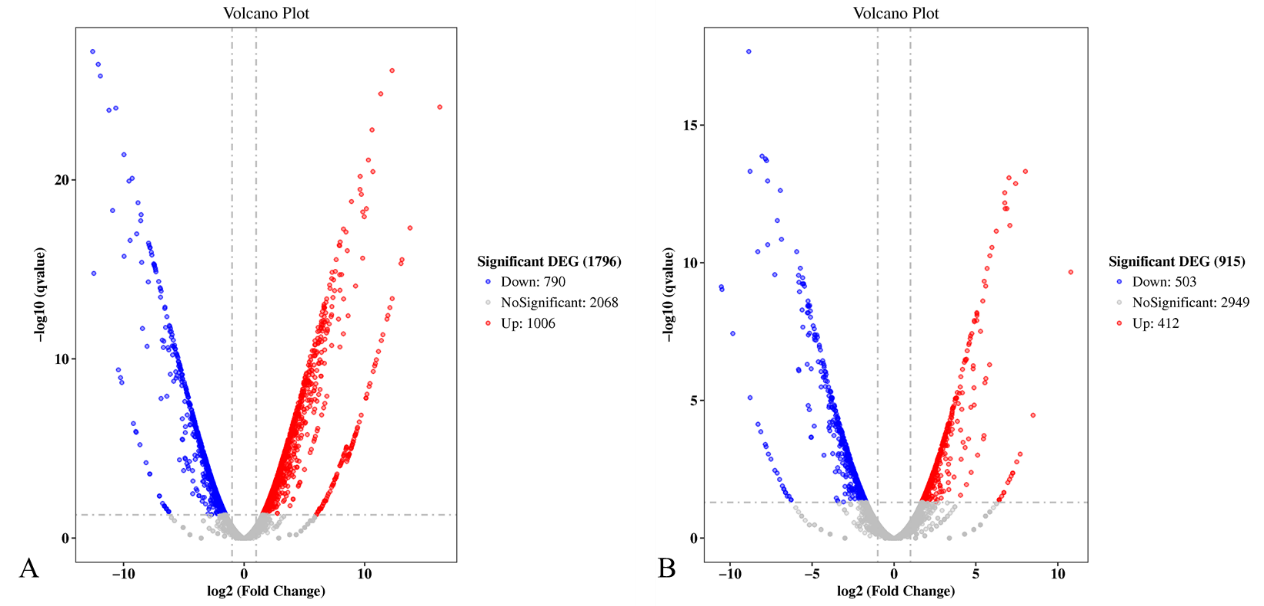


**Fig S5.** Volcano plot of the differentially expressed genes for the groups 0 h VS 8 h (A) and 8 h VS 20 h (B) when *Bacillus* sp. 8A6 grew in feather medium. Up-regulated genes are shown as red dots and down-regulated genes are shown as blue dots. The significant difference means that the expression level of the genes are 2 times different and the qvalue (fdr, pad) ≤0.05.


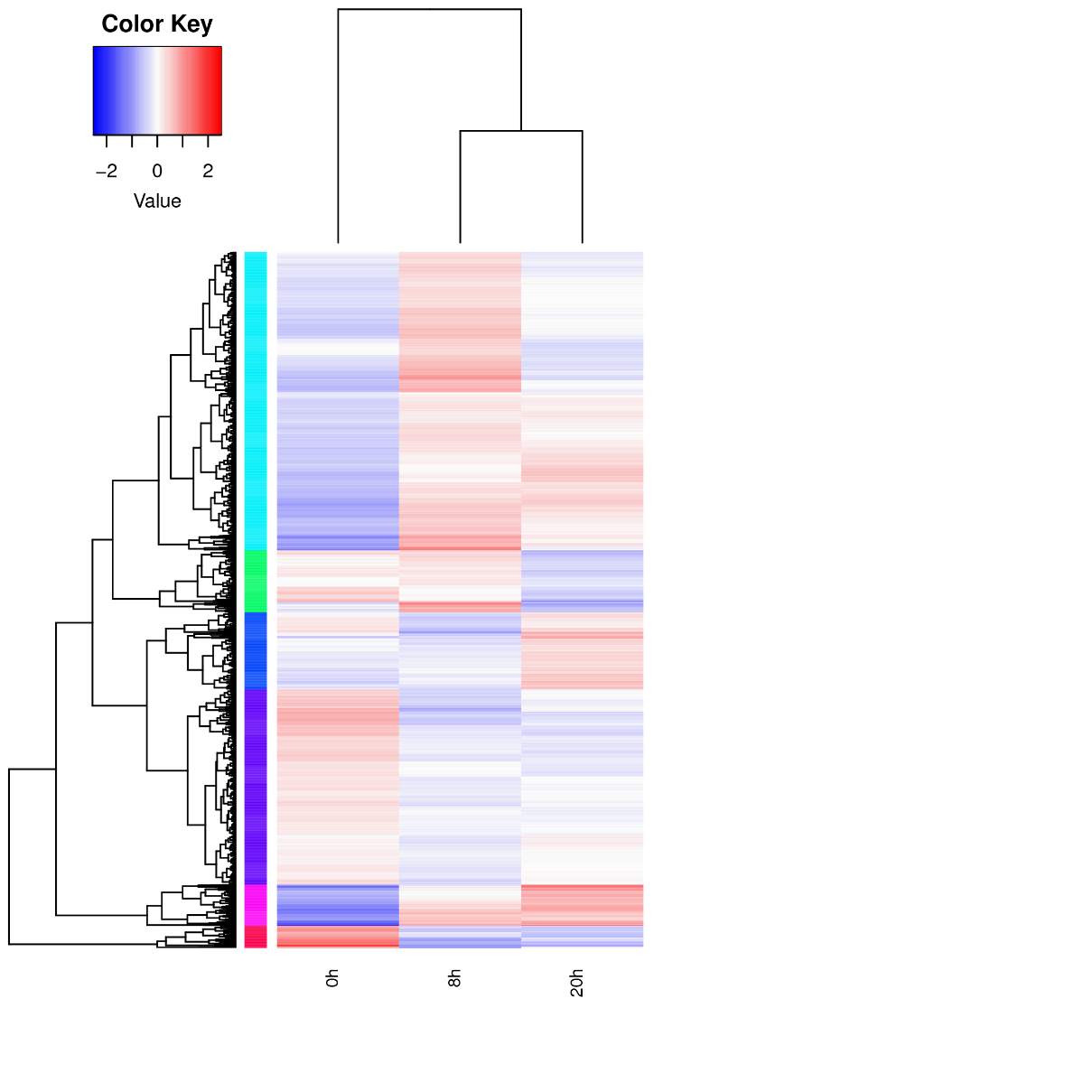


**Fig S6.** Hierarchical clusters of differentially expressed genes according to the log_10_(FPKM+1) value when *Bacillus* sp. 8A6 grew in feather medium for 0, 8 and 20 h. The up-regulated genes were indicated as red color and the down-regulated genes were indicated as blue color based on the color key. Six main clusters were enriched and highlighted by different colors next to the phylogenetic tree.


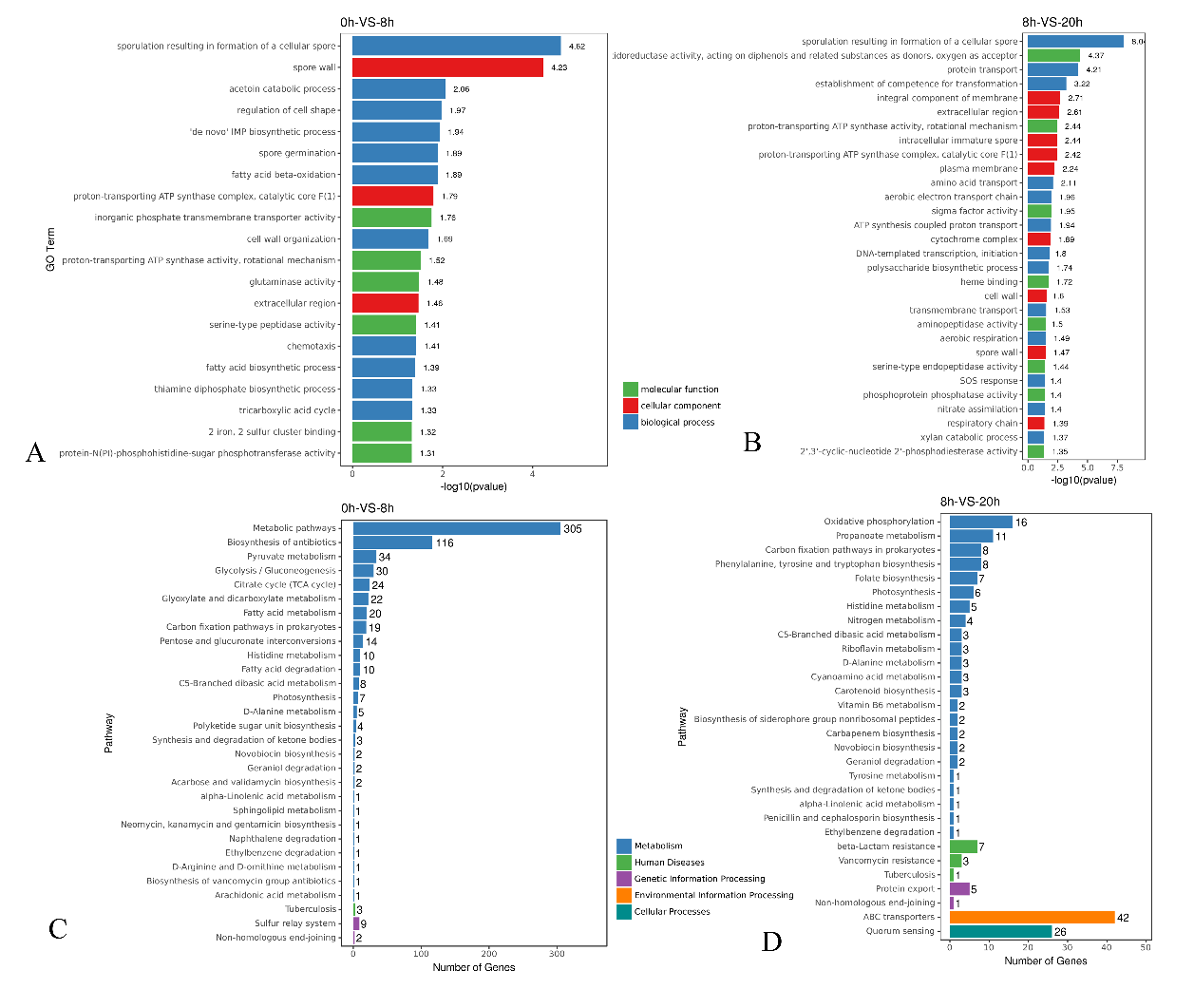


**Fig S7.** Gene Ontology (GO) analysis of the molecular function, cellular component and biological process for the groups 0 h VS 8 h (A) and 8 h VS 20 h (B) according to the -log_10_(*p*-value) when *Bacillus* sp. 8A6 grew in feather medium. The higher -log_10_(*p*-value) indicates the more significant for enrichment of the GO term. Kyoto Encyclopedia of Genes and Genomes (KEGG) analysis of the pathways for the groups 0 h VS 8 h (C) and 8 h VS 20 h (D) according to the -log_10_(*p*-value) when *Bacillus* sp. 8A6 grew in feather medium. 30 significantly different pathways are shown according to the Q-value.


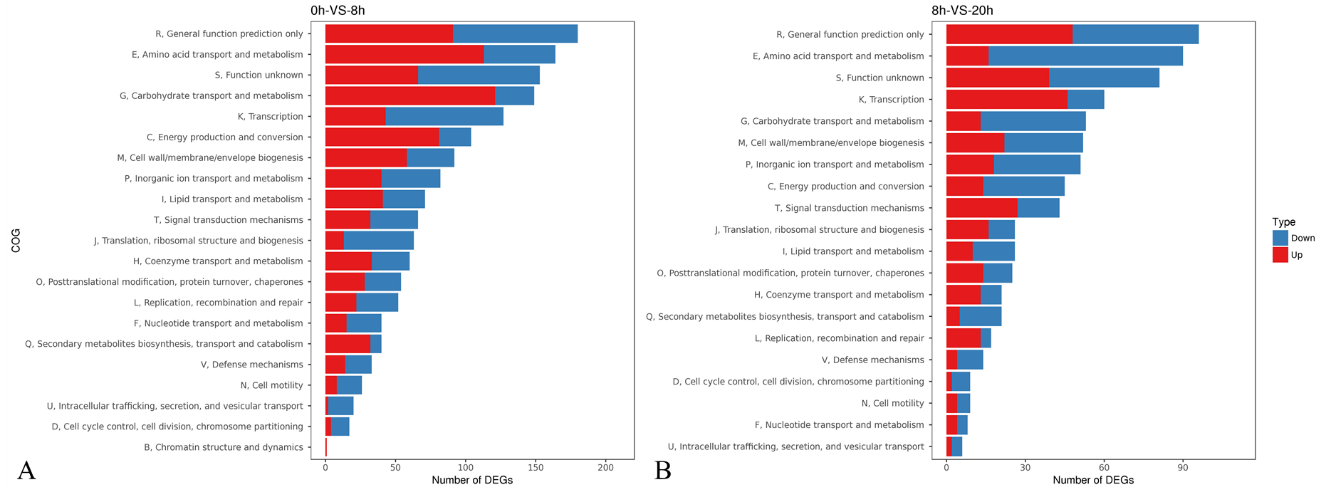


**Fig S8.** Cluster of Orthologous Groups of proteins (COG) analysis of the gene function for the groups 0 h VS 8 h (A) and 8 h VS 20 h (B) according to the gene numbers when *Bacillus* sp. 8A6 grew in feather medium. The up-regulated genes were indicated as red color and the down-regulated genes were indicated as blue color.

**Supplemental peptide list 1** Short peptides of *Bacillus* sp. 8A6 keratinases

| G(+42.01)VSTM(+15.99)ALLAVPT | SVKKGLEC(+57.02)VTP | K(+42.01)MAALHHAVPTG | LVPPNTKGMPR | SSSNGGTGWAELK | S(+42.01)QFEQHAFLKYQ | AYVVFVAAGHDKYQ | QPSPTLPAAPQNK | K(+42.01)DETLC(+57.02)TMR | TAAC(+57.02)QKMLN |
| --- | --- | --- | --- | --- | --- | --- | --- | --- | --- |
| PFQNKC(+57.02)A | QPFNKC(+57.02)A | QPFNKC(+57.02)A | DSFKDVLPVSN | VNPESQQGSPR | Q(+42.01)SQQRQQQEEQPLEVR | T(+42.01)NRTHTVAMEEQLPEVR | TVAMKPLPYSN | FGGTNEDTAEKL | TDVGDEAKSLK |
| LVNNDDRDSYR | RLDYR | KPPGVKDT | VGPPLVPY | PGPVLVYP | VGPPLVPY | GPVPLVPY | PGPVLVPY | PGPVLVM(+15.99)L | GPPVLVLM(+15.99) |
| LAGNFDPSMYSSAF | S(+42.01)EGVNLC(+57.02)TMK | FNQRSPQ | QPGPVPEKNGLAQNPAAP | LVPPQESQKR | TEGMKLTGMDLELLETW | LDQMRPR | KDFLQQC(+57.02)NN | TEVPFSWLQR | EHALFS |
| FPPTADNNPFY | LMAHSTTDPFY | FGGSKPNT | VLPPQEVRM(+15.99)PV | KGFFEL | KLFLFNTDPGYFLE | RLDFDLL | AGGPLTTAEQEQPLEVR | S(+42.01)DTSSTHGKYQ | K(+42.01)STC(+57.02)LTEDTR |
| NGAGDAAEFLKY | QEPQQNEC(+57.02)QLQK | QPDDDDGEKRVQK | SC(+57.02)GKTAL | TGGHENPEGLQ | NALHM(+15.99)LGLQTH | LAGEKDVNVRQ | KLTFLLEEAGMPPLDP | VLPPQETKFPP | LVPPQEFLNVP |
| SVLVFVDPPTNPEF | S(+42.01)DAKLRAC(+57.02)AMC(+57.02)LQ | KLSAM(+15.99)AANMC(+57.02)A | LQGENQDSDLQLVTL | TGSAHNSVK | TLM(+15.99)QHVR | APGVGLKPVGM(+15.99)SVS | TDEAFGLC(+57.02)PS | LAPVGGKSSKHFS | V(+42.01)TFLEHA |
| TPAAGDQKC(+57.02)KNP | NGGVYTPDGQTM | VLNEADANLELVGLK | TLVQFG | TLSDTKM(+15.99)NEPPG | SPQVQSASLAGEAHVGVFE | TVGTVGGADLGASK | VPNKWSWLT | AFPGSAQDPVTVT | GPSAFAQMALPVT |
| SLQEVRP | EQVVLGNYYQ | KEDHPQPQQNEC(+57.02)QLQK | Q(+42.01)SPQSYNAVPEC(+57.02)GPPTGSN | L(+42.01)SGQQDEEDSGALVTL | TNPVTKSGVVFE | TTERKLTGGVDT | ALVRNPTGAH | QPAGHAASGPQAGS | TLQM(+15.99)NKTVVAL |
| LMGSPFVTVVLA | TLSSHHAVVVAL | KPTGMPLYSNK | TLSSKNTTVVAL | AVVPFGC(+57.02)EDGAVS | S(+42.01)ENPRVKLSE | VYAEPTAT | GHPPTVFP | TVHKHLT | KGGVC(+57.02)SDFGRQF |
| TDEAVLC(+57.02)TM | PALDMM(+15.99)VHPAVT | KTDYFEELNK | AM(+15.99)PPQC(+57.02)SC(+57.02)EDLRLN | M(+15.99)APGDDDHGPLMNALH | AM(+15.99)PPQM(+15.99)EMRDPKPM(+15.99) | PHVDPH | TLSKPVGPAADPG | ERSYF | RAFEPPRYEQ |
| LTFAFVGPTNVKMNVLSF | KTEVVGEAGAGSK | ENQLDQMRPR | SDNFEYVSFK | SDNFEYVSFK | KADDGDYWLAK | TAAKPVDGN | VTPGPGVPEKTGWTLEVF | TDDDYKKKC(+57.02)A | Q(+42.01)SGVALEPTFEEDGFGQSN |
| LVPENSPQVAAR | PVVHGTDENNMP | AGGLHETFYNAL | KLDPLNRESE | PDLEYGGGPETM | DPLEYNGEPTM | KTMM(+15.99)FEELNK | WPSVM(+15.99)HPELK | V(+42.01)TFLEH | KLNPMFP |
| A(+42.01)YDSFDEVHGL | NNNPFKF | LMNMPKF | WNPNNKFPQC(+57.02) | LAGNQEQEFLK | AHVALLPSPNQ | S(+42.01)GRFYNPKQR | VVNDPDNELLPPNHG | ASAAYYGKEFNLKLETK | TQPVDNDGLVS |
| TDLGM(+15.99)YMSSH | DRPSLGN | EVC(+57.02)NNLPTVVQ | TAALAYMNANR | TDEAYLC(+57.02)SPR | NLVSLSPK | S(+42.01)EPAHVLRGGPQE | EERVFRTAGPQE | TLAVM(+15.99)MLDFGP | LTEGTTMQAMLELNQTL |
| LESEGGLLETR | TDTDFC(+57.02)YKPC(+57.02) | KNTEGDGALNNL | S(+42.01)NNVLGGTLSTSVFF | KSTVEAPAFENN | TQLEPPSH | TQLEPPHS | TQLEPPSH | TAAEVPPHS | NQPPELNP |
| TGAKNPPSH | TQKNPPHS | KLFYQ | TVM(+15.99)QKSHLR | ALLPVNKSHSFKS | ALLPVNKFDWPK | DPKNPWNPDKLNLETK | LGSTGNVC(+57.02)PSR | TWDQTTANNPN | NLATDDDYAKYVGP |
| WGALFNEGASKEMMGGSLL | TC(+57.02)GLMVDPQ | NALGRSYMTQT | LLFFDAWC(+57.02)GL | TTGPVPRK | R(+42.01)GSLGPVK | S(+42.01)GTNQSHPELK | LVYDRPT | LVYQPFP | LVQM(+15.99)KQT |
| LVFWTPT | PQKSKNPAGLSTVP | PETLKNQPDLR | TC(+57.02)TKTEENAPS | SPSYR | TDSLSTKRST | PPQTGTHEC(+57.02)AR | GKGALGFPGGGLVPA | STAEPWT | KLDVFRALPSE |
| TFSGQQRDNNL | KLDVFR | STYKST | KLPYRATLSP | SPQEAY | SVLYVTR | LDGAKNSTTPGF | EAGPGDPM(+15.99)FTAKEPQEQSN | KDEHVPLYSNK | ELSPM(+15.99)NFEYV |
| KLVPTPD | GPSAKDLM(+15.99)GKQN | TNVLLQSPVQE | KTNNDEFNNY | EPFGSLQAPEF | KLSSHGDSEGGLKLLE | PGSAKVENM(+15.99)VLT | LKDVFR | QNGRSLM(+15.99)EQTM | ADNVDTVLLPKA |
| NDVATDLLVPKA | LVFC(+57.02)PQ | DPTPHETKHTVN | TNTGKDQMHVR | V(+42.01)TLVGLK | LETGFLSPMSGGLTAAQTW | SDNFEYVSF | LVNPESQQGSRP | GPSAPQFHPPN | TLDKSENTLQ |
| THVDPEAH | PGSAVC(+57.02)HLAGSLGFFGMVQ | KGGALGF | EVELKESPPTDAAVQ | PTVGNTKNPATQPTELLF | TTGQADSNGHGH | LSSYLGKQGMNKYLYLQ | TVSLC(+57.02)PQ | EGSHTSEVYLGQ | T(+42.01)SSHSKETESLDEEEGGP |
| FEELNKVLFGR | QC(+57.02)C(+57.02)PTVK | KSPLC(+57.02)QC(+57.02) | SLNRPDGN | TVVDDPKHYL | A(+42.01)KGWDAGSSGPDGLVTF | KLM(+15.99)ESHTLDPQLLE | TAPLTNTY | RVPSGTTY | RVLDNTY |
| KLPDTNNPKSSNFLETR | SSPDLYNM(+15.99)DLS | TTDGM(+15.99)HVLSSGH | FVSVGDNHKQLVT | GPSAFKAW | TPKC(+57.02)GPAHSDTL | KSNDNELSYTL | TPKSSENGYSTL | LTDKAGATLHLPPVPDY | YNDQDAFTVAV |
| LTPC(+57.02)EKH | SAAFSVN | KLFAEGV | TEGDC(+57.02)KVRLQ | PDAVRGESENLKLLETK | MRLYTFKQTTELYLQ | QPPELVSQ | PPQELVSQ | EAGPPLVSQ | VPVVGYC(+57.02)C(+57.02)SGC(+57.02)DGM(+15.99)FAPAVVNAN |
| VTFRAALN | QELPPTDHDPPAAGGPATVQ | VLRKMNGG | KLAHADTM(+15.99)KNLGF | PELTPHS | VNNDDRDSYLR | KDNTGQNNVLLF | EGGTSGM(+15.99)MKVWA | LSSEDEPFNLR | SPHM(+15.99)VR |
| TGLHGKNYSTL | SVGVANC(+57.02)DKVAH | C(+57.02)GPALPETFEEGDFGGASN | ANLGAAPRDGAH | S(+42.01)ELPLAE | ALFKAPGS | AADPSTSQLDKKM(+15.99)AA | G(+42.01)FGPSLR | TPKGAAPT | KLLKMPTTDV |
| AFDDKELLSL | NPLTNPNPELGL | TNPGQC(+57.02)RLC(+57.02) | GLNTRPGAG | SETAFKKKQKEELEDTLMEHS | TQLVVK | KNQRTVLDKF | KDGAGVLTVNKF | KSLGQLLENF | LSVSLDAK |
| HQSNWPELKAC(+57.02) | SYGFLTM(+15.99)M(+15.99)QFG | TAARALEAS | SSSKSRPQ | QPKEVSPQ | FEEDLDAMLPK | DLVADHNQAF | VTGTEVEEEDPELVTC(+57.02)K | DPGNDYEFVTM | GGGPDLEM(+15.99)TYTM |
| TVFGNDLPSSL | QDGAEVALM(+15.99)SLKYQ | A(+42.01)AFRTLKLPSE | TPGVKSNAVPGT | SFENNEYVSF | PDAGTTKASNPNQ | WEVLEH | THKQNPLYSN | KDM(+15.99)YLK | KTPEDTETLRNPDAT |
| TPVGNGKNGQRGGPLHPEVF | KGLLVELHM(+15.99)T | EPEVLAGGLLQQ | VLEHGGLATDDDYPYRKA | LHLSEAFPYPQ | TRAC(+57.02)M(+15.99)LM(+15.99)Q | KMTGPLPYSNK | KLKAGNNTAAPN | SNPTTKKMSN | C(+57.02)NGKLSGM(+15.99)NGELQERGVL |
| TFLLC(+57.02)GPGNS | TLLVPDAH | DPAAYKLPPSGPSELQTQ | QLTVEVKDGY | GAPPSC(+57.02)M(+15.99)QC(+57.02)EDLRLN | KGDPNEFLLKG | M(+42.01)TGHVVR | LVRDTQPKPR | S(+42.01)NNVLTNLDDGQFK | KPFYNQ |
| F(+42.01)GTVSL | TGGSVLENL | SFGEHVT | KM(+15.99)GGQDLFLHD | LVPVVSM(+15.99)VPPVR | LVPLTAYLKPPP | EFLTKDPQPQ | LATFLATM | DPNVVNNELNRL | RVSDDEFNNYK |
| KSEGGFPQ | SELPVAH | TSSC(+57.02)FPQ | LNALM(+15.99)TLEDPLKMM(+15.99)KSDP | KLMMSF | KGFANHNT | SSPDLGNDTHQ | ELAHGQESPDKGGALLTQ | FLAADKDNVVRQ | DPLLEGNP |
| PSEMVNFYNGV | KRAGNSSSSVR | EPEVMVPYMRP | TVM(+15.99)LHSPYMRP | TLHFPESEGGLKL | N(+42.01)SVLYK | SGVNPLP | FGTLELAPMAFHPNLDHQ | TTAALAVLQ | AYSVPH |
| RSGRAASLKHVNERPER | KFNLGM | EPTHDNLDPGP | QPGAYAFANQFGSHSSNPK | TGGLFPR | HGPPVYPYFPQ | PPGHYVQAAHPQ | GSAAAEDYWLAK | FRGLPNS | TLVSMGFP |
| TGFVKYH | TSSLVTPF | TLEKM(+15.99)TSSPQ | YNPKQR | HLPGDAQR | N(+42.01)KTPERQDDDDEEEQPQC(+57.02)VEESHRSEKRHTP | TLTGGNM(+15.99)QQLLLN | TPC(+57.02)EKH | PDLRNESEGGLKLLETR | L(+42.01)PVYDHHMYVSF |
| TYKFSEDPPTGGP | TGMPPGVHKGGAASE | TVM(+15.99)YSTN | KNVKATTFET | KSAAPTRT | KKMNNLYSPH | KDTFVGAATAAAH | SLVDLTN | TSLSVVFP | SSPRDADLTSSL |
| S(+42.01)WSVVEDLPEGPAVK | KDSSENPQLQGV | LNAEHDLYM(+15.99)V | KLAFGEVN | S(+42.01)GGVFVDEGALWNLV | DPKYVMSEGGQGGLLETK | TVGSAPNSL | ERQPQQNEC(+57.02)QLQR | TVAADGQPTKTSE | VVLNDM(+15.99)AEENLR |
| VVLEWC(+57.02)GALSDGL | DFFKVNLDT | V(+42.01)HDLLPMDQQ | TTKQPGSAK | TGGLVEKAS | LLDTNSLENQLDQMRPR | TGSGWHDM(+15.99)APGTDDKTLQ | PQRVTTY | TVGLLAAM(+15.99)GPAPLK | QTKMYQEEGAPLEVR |
| KGLFFNTVYP | LRKVPA | GPAEGETFPEEFYMAGSN | LLDPSTSEPGHPM(+15.99)MQRPR | TNVVALVGNC(+57.02)LPH | KDDDSLM(+15.99)VN | TTLLVADQVAM(+15.99)T | QRGPAKEEDDDDEEEQPQQMEDTKRRPM(+15.99)ALAEM(+15.99) | KMFNESPAEK | KFPNSHLQNL |
| KNGVNLQE | DVFRALSP | TVGELHKAEYK | KTEVQSTPAASR | LSAYLL | TGPGPGASLAPHLGT | LVDDNDDRSYR | KNQFLGM | VVNC(+57.02)DPVTGHPDGELQEGR | TPDQNVGAGSPR |
| GLATPKGK | PVNKFRGP | FHSEFLM(+15.99)AKRTH | SQSPTFK | KLVC(+57.02)FPQ | LDGTALVSL | LSVGMTLKLPSEVLAH | AAAPESQQSGPVR | TAAGADNMVKAL | TGDEKMLLLGPT |
| N(+42.01)SLTQATAVLL | A(+42.01)KGPNTNLQLVN | G(+42.01)KLTM(+15.99)GLNLEAS | HLLEAFSPYMPR | LHLTM(+15.99)VSPYMPR | EQPGMRGFLQQ | TVNKDQLLLC(+57.02) | SFHADGELLYL | LVTGGDFVVTPP | FHSEFEELNR |
| TGQLVM(+15.99)PH | TNEFWVLEH | TLVVPVR | KNLGPKGTSPVGAP | TAHELLR | TVGFEASL | ANLLLPEE | SDKLYQTC(+57.02)GA | TAARPDM(+15.99)VQ | N(+42.01)TC(+57.02)DQYKSSEDDSSSSM(+15.99)S |
| LPVNKFGESPR | KLDRVFALESPVLSN | FALVDAM(+15.99)T | TASKFPEL | APSEHLVGAPSEN | DPNNRSTMENLRLLTL | KLDVAFRLPSE | LALPVGGKHNLTGSPN | EQVYSQQPMNEQLPEVR | TTARPSVQ |
| TVSESQFVLHAENEGGSLL | F(+42.01)DQAAHNNSPQ | LAGGEPVHVC(+57.02)ELTSYVFV | HLSPYDGRSGTKS | TLHHEKDTAT | QPNVHGQGPAAGS | SLQGFPQ | DPSSLYNPPTHETL | TSLPM(+15.99)NSDLK | QAGGSSTEGPHNVFK |
| EHGGLVLATDDDYPYRAK | KHTSNR | GPSSTTLQ | GVTPHDQPEKNEC(+57.02)QLQK | APANLKRAGPNVVSC(+57.02) | TFAPFAEE | TDARFFGGVPSGN | G(+42.01)KALPEEFEVPGA | TVGADLVLS | TVPMGAGPSM(+15.99)VS |
| G(+42.01)SFYNPK | NGADYGEFNARP | TDAPM(+15.99)LNFEY | G(+42.01)KAEDNHVTAH | ATGADFLFTLETSPYLAF | EQLSYAPLTPGELYSGPGA | QGSALTDVA | GPSALAFQA | SQM(+15.99)C(+57.02)GYYVSF | A(+42.01)GSVM(+15.99)M(+15.99)ETDSL |
| TVSNKSPK | HLLTVFEVFGGQVSPQGVQ | TPPKLVNTAH | TLGTPWLLTY | LAWVTDVLTNLEEREF | WVVDLF | KSEQLRQ | TENVYGF | KNYDGEELVLKS | ENGAQSTGALFLPH |
| TAAQKLVT | TFASPQDRGLR | PDGGRLESEGGLAGFLETK | KDNTVYDLFKN | TPLTEQRAVT | GPSAFAWGSLPVT | HEC(+57.02)PVQLMEK | TVRYRPT | ELTWNPN | SVGVPNMGPDTVV |
| S(+42.01)AGPAAFYEPPRLYLQ | S(+42.01)RFTLLKLAGF | TSRDPFN | QSPVGAFLTMLLAGNGPQE | S(+42.01)NQVGFPLTVVQSPNALQN | LSDTQPRG | DGARLKDTTTGN | QEGFYEMRNPVFK | QASGC(+57.02)FPATLC(+57.02) | S(+42.01)ATLVVAQ |
| D(+42.01)NGDSHSVYEFNSHPSF | APEFLKEA | FQPGMEEQLRAL | SSSAHLFFNF | TGGNTSPT | TFAASQPN | QPAADPVVFFVTAPP | DTSFLAGDLNTLGTNPASY | LLGTPHAKYPS | PQVKVPGGFELGY |
| MC(+57.02)RFGMP | KEPC(+57.02)H | KEGTLEWYEEPGAKQ | EC(+57.02)LPLATAHPS | P(+42.01)GEHNHDSYR | SRPTYSK | LAGNQEQEFLKY | LGAVSGKGANVPGT | AMKGYLLDPQFGFAHLQQ | NEGALLLVDLPH |
| DPVTAFKSGKKPVLAH | NNNPFSF | VTLVEC(+57.02)GM(+15.99)DSTHLVTVEGG | M(+15.99)TGGLGTDDM(+15.99)YKC(+57.02)KAK | TDNHSGVALSPK | LGQTNVRQ | LLADPDGPL | FNNQLDQTPR | FPTDEDNQGSPFTDL | VLGLVGQGADPLSDSGVKEAGPLEVR |
| QPSKTLPN | ENQPFAAKSNQ | QEPQEPPHGLPN | KSPNTLLLPH | QEPQESQQRGR | EGKKPASVDGAFVAN | RGVTSSLLADDFVREGNSC(+57.02) | TLYPDM(+15.99)PQTL | GAPAQVSKGTQEQP | K(+42.01)GVEFDDGSDDALQEGR |
| WSLLR | FALTVVFP | PHMVTSLQ | NELTAASLHVQASKPLRLP | DTSNFNNLQDQTPR | TDPKFLSPEVL | EHM(+15.99)TTSYRLQ | S(+42.01)LGKM(+15.99)YNLPDELLE | LATMKM(+15.99)STMC(+57.02) | Q(+42.01)SQQEGSSGPGAPLEVR |
| KDGPSNVSLLF | PQGAAYFPQQC(+57.02)M(+15.99)SQLQK | GSFAPEVTPGGW | G(+42.01)KALHEYTLNP | TEAPLNPK | TLGPLNEVSLPH | LLAC(+57.02)GPM(+15.99)LTY | TLYEAASGARMQ | FRLPTVT | DPLC(+57.02)M(+15.99)M(+15.99)DAKNVLQ |
| KTEKVGQDLQTR | TGVLLFPQ | SFMSSPK | K(+42.01)GQGC(+57.02)ETAAPDSTGNLRSPA | AAVDGSLAR | APLAVMYEGC(+57.02)GN | SANWGF | RSQSDNFEYVSFK | QPPLVES | THTFKLDNLN |
| FVDDMVT | LMENQSEELEEKQKKK | KQDGVLF | P(+42.01)LTHPATLPLPPEPREGR | DTGPSLRVM(+15.99)M(+15.99)TMTPC(+57.02)QC(+57.02) | QPKPLQTAHSNK | G(+42.01)TFLSGR | TDKC(+57.02)C(+57.02)MSEL | K(+42.01)AHRVYDSGDLGM(+15.99)EHDDRGTR | LSLVQSAAGLQGTSLPH |
| TFGKSQARKHS | PSEVPAEGALTKSN | YSPYMRP | SYPYMRP | HGYQVR | KFFFTR | PPKSHLY | KNVMLAFESTVP | LVDQLSFNLGDSGLK | TKMMPLQ |
| TVSHKTVLSN | TGAAEGVSL | SPKRELA | T(+42.01)VFPKAAGYPVP | TPLVHMMGVP | LPSYKTTSVNK | HNAVTSY | GPC(+57.02)PETFEEQKTESNQHA | YNVTEQPLVNP | GVAPDM(+15.99)TNTM |
| LQHFGR | STVYLPVWNFYTLYLQ | TFNLKSQ | KFNQYGR | TVGPNLGYAKSGA | KEALFFG | TGASKM(+15.99)EYWAAVP | C(+57.02)ADKGVLF | SAANKYDFGHT | SSDFLERKSM(+15.99)GDDT |
| SANKYVPH | TLLLPH | P(+42.01)SGYPVEPGAPVGGLLSGGK | KFTDNKPLDGN | C(+57.02)LGGFSNK | TLLPVLYPNF | TVC(+57.02)DLTKKVL | KGLKLF | KC(+57.02)AGVDLFNF | VLDVMNGPKLNAMGPA |
| HPYFVT | VNDLAKE | TASYKAFLAE | KMKQC(+57.02)QAGDHSNQK | KERAAC(+57.02)NDDDPLQK | QVALEPDNESHKAGQ | PVTKYTC(+57.02)C(+57.02)C(+57.02)HVAV | TM(+15.99)VM(+15.99)FAH | KNLSDDLLQQLVME | TELADDKLLHG |
| FHSEFEELVNR | SPHM(+15.99)VR | TVFFHDLQTR | QPLVNFVVAAR | LLHRLDYN | KLC(+57.02)SMFTKPQ | KRSAC(+57.02)NM(+15.99)YK | TVLVDFM(+15.99)QNT | LSGGGPGASAVGVEKLTPDGGTH | KGGTGWSFGRKF |
| TLGSFTVNQRG | KVFVLLYHHVLQ | TGGRGVEWNPH | SSAYPVAQ | G(+42.01)VSAFHPGGDPLELLETR | AYNQQVVVLTTFSF | S(+42.01)QPPVAAQRDGH | A(+42.01)ASVFALKLPSE | PQAALC(+57.02)GHLVNH | KFHHVLFKNQ |
| SVLKPEPS | APQSYC(+57.02)F | PAQSYC(+57.02)F | QAPSYC(+57.02)F | SFSHKAAT | GTDWWSHDAADSDKRW | TVVNNDDRDSYR | S(+42.01)NPDALRPNQ | ASSHKVVDC(+57.02)DDDDKRW | HDC(+57.02)SPGHPPASWDKWR |
| SPSNGTSHFVC(+57.02) | TGLC(+57.02)KNMPGT | LVGNTSQAPTR | KNHAFTTTLYR | LMKSLTPQ | QGLAAPVEYSHT | DTAAYRLVNEPFNLR | D(+42.01)SLAEKL | GLDPHTWQSMTLC(+57.02)SPR | V(+42.01)TASYSLFKAAHEVP |
| TRLLFLK | EPLSVQM(+15.99)M(+15.99)EEK | PHMGPPYM(+15.99)PSPAC(+57.02)KLQ | KEADTNSPFLE | SVAAHNEPKC(+57.02)AG | N(+42.01)SLGKDLASLTVSPY | ALTFEPKS | TLVGFLDQ | QLSVPMFGFVHVGERDQWKGR | RDGSTLYSKPR |
| S(+42.01)TYDGSSVKAALQ | TATKHHS | VVAALDEELNNKFGSF | AGALGPERAGQHT | LLDSFPPDREP | LSETNYF | TVSC(+57.02)FGMPLQ | QDGHPAPR | ESRYFSSLKLL | SLHSTPLFAADL |
| STAPAADDLTSS | K(+42.01)GSAAM(+15.99)VPTKGGVL | TVNGSKKGHVLPH | VSLEC(+57.02)PNTTFC(+57.02)SALQEGR | QPAAHDKDFSRT | TFGNPVT | TAGPAESTLPKA | KFLVTH | SDC(+57.02)GPPLK | DRNHPDSWTSK |
| SLAAFPHM | S(+42.01)GHRSSEVYLGQ | KLM(+15.99)M(+15.99)VGVVM(+15.99)MLKLETF | QERLAGAAHC(+57.02)A | KMEFVVPVTL | KDRTPH | YNNEDTPVVAK | TNHPELK | LVPQVN | TPKVVLE |
| NEGEVSDVLLLPH | GFGC(+57.02)GHVVFFN | RFVDALPSEVLAH | D(+42.01)SMLKMLYVPPSH | K(+42.01)M(+15.99)YKTTLPF | GHEAKQLT | KHMKKLTPH | PNSELHEGLTC(+57.02)R | NPSELHEGLTC(+57.02)R | ESVLVAAVELSKRSSAVP |
| KPM(+15.99)YFVDAQPK | P(+42.01)ALPFVR | SSSYPM(+15.99)QTFNT | DPGGVVNDEMNLR | LPYQLTSYSN | EPGVFEALVFPNDPATL | TPM(+15.99)FV | Q(+42.01)DAHANNQLDQNPR | NVLSQLAAGLM(+15.99)QVFF | TQYNPKGMPAP |
| FGGAMGVEMTLQ | SPEMYGGFVDKAM(+15.99)GVVT | V(+42.01)TTFGPKC(+57.02)SSVGH | VVNAAFADDFGM(+15.99)MDVLLPH | TDAALVKQSQ | DELQNSLAVRVPYH | KDAPLPDEELSPVPT | LAGMGFYNPKAGR | SSPLKPQSM(+15.99)AF | TGNQKKVFPRT |
| TLGKNMK | KMLTPSQ | THTTGM(+15.99)HVRTETADLN | KLGLDTPLLSK | LVSLKP | TFAAKVVSSTFN | TLGAFSLKTKPFL | TGTGWDHPELK | LLVPM(+15.99)SSYKLR | LTPEKNTGLDMQVFLTTV |
| TGEC(+57.02)LSYVLFK | QVGESM(+15.99)EELNRVL | LVC(+57.02)GPHTL | HSM(+15.99)VTKREGM(+15.99)S | KTNC(+57.02)GPTSTTY | GPSAAFAEHRYKPGGT | ERDKYVFTDQ | DGPKLEH | TNERLPVS | QLSKGWVV |
| TVEGHDQTPVR | S(+42.01)HANPVPS | EYVSGKTHGKN | TGQDVELR | TVAVPVDNKKN | KAELVDNLQFVFF | TDPAGFGKNLTC(+57.02)MLSPSEVLAH | TAFDWELNVK | TVLPLPDHSM(+15.99)L | A(+42.01)KGC(+57.02)GMVVMHMPVTK |
| TANPLM(+15.99)T | TSFELVPVKM(+15.99)P | SHNLKNTS | QPFGMPAGQKEEGSKGR | SWDRVL | N(+42.01)TGMSLLPHQQ | SLRLN | K(+42.01)LAENGHHSLQ | EVSTAFDNSKKTVDT | PVQADNVLSTGL |
| NGAPVKYKSGLN | EEADPVGHSTLK | LGGGADSTEMLAF | EPKNPDPEKNPQ | SPKMC(+57.02)H | EQTM(+15.99)VPALTQQ | ELDPASTTEFDNYK | KFNQFHLTN | STVNFKGG | DHYDVFMPY |
| P(+42.01)SEVTPLTQHT | LLKM(+15.99)MEGVHTAK | SLTM(+15.99)QFERT | KLLLLLLLGGN | KTGALSM(+15.99)P | NSQHWLAPGAC(+57.02) | SLGYM(+15.99)VR | NDNPFKF | GGKVAYAGGVASEEFKTME | G(+42.01)KTHLTDVTMLLPH |
| KDRMVGFGAAAH | APSFLLQK | TGEFSGLR | KNVLLFERT | TVAC(+57.02)PLKFPF | P(+42.01)LTYELFEYTSF | VVNAAFEGDSVGEDSLQEGR | KDHDPLYSNK | KLMTTAALM(+15.99)VTL | TDEVALC(+57.02)T |
| HLGTRPDS | SAMM(+15.99)TYK | K(+42.01)SPDHQAKC(+57.02)NEC(+57.02)QLQK | VLNEDGSFNTLV | KLAEHM(+15.99)SGFLKYQ | V(+42.01)MHM(+15.99)VSFMPY | SSSLDNHSFSK | DQLKPKDNLDQPKLRPL | TNNSKDFEYVS | QNHTTGPRVMQ |
| KQGVTKEVQGDLQSK | QGTPLFPR | VYGGKKLNVDPNDGSETL | TPGGKMM(+15.99)S | S(+42.01)APKSLAG | G(+42.01)KEENARM(+15.99)YM(+15.99)EQP | TLAEYKHGNPQ | KNYKAATYM(+15.99)DKLLEF | THPEPH | DRNPHWTSDASK |
| C(+57.02)AGVTM(+15.99)DPQKAN | KVDPTQGHSSDP | LVETFEENLNLGEK | KLYADFFNN | VQLSVM(+15.99)EVPVY | TVLYALDRLQ | RSNELDKTGAVLL | PDLNPSYPAAEEQKKKATSETR | TAFASFHLLAP | KM(+15.99)NNNPMLYP |
| DPLNQSEVKKKTLEQNTYTEKE | TVGTFHDLQTR | KLSSSTTGWAF | KPDSTLRC(+57.02)Q | TEKAHLLHPH | P(+42.01)VPVFRL | VLPGKLTTVFHP | KNVPLLAPG | KPELVPS | G(+42.01)KEALLVPGFMT |
| STGFGC(+57.02)K | TPGGTFGKSAVPGT | STDSHGL | EPTM(+15.99)LK | TRAKNVPHEFTQ | DKGQVC(+57.02)GGSDQGLLSDDPGDLLTAAP | GAPGDTQYAC(+57.02)DLR | SVLVPM(+15.99)T | KAFRFYLM(+15.99)K | STNEVLAP |
| EGALKDNVVLQR | DDDGNRQPLTM(+15.99)HW | EQEENTNDLL | KGPTAAC(+57.02)SLDQV | TNSYEM(+15.99)KHY | ESSYFKC(+57.02)M(+15.99)PQ | NVLFMKHLTNP | KGVLKEGKDNGTEEGHMQ | NTDNVLSAGLGHMAFF | KNTDRSYELT |
| GNGAFWT | PAYALVGAK | SLEVC(+57.02)DF | STNALPMP | APGC(+57.02)NKNAM(+15.99)VRS | QNC(+57.02)GVTPM(+15.99)LK | SLLKFKSAYTWPH | EDLTLPNHGSLH | GHLTVPK | KVLTSNR |
| TVKLLPSF | TVGLPM(+15.99)ANVPSQ | TYM(+15.99)M(+15.99)NKLGQ | TPESSSF | STLLNTAFKY | SNM(+15.99)SPPVGT | EQDHHPLLYLL | TAAWFNYPK | ANLEMQLAPAEQGKLLE | EPAALNNPGPV |
| GPAGAYGMALPHHNKQPR | KESAWNSTVLT | LVVELSKEQLAR | TLGGLSLEYVGTGL | A(+42.01)SLTLQR | T(+42.01)GVTLSAR | SRPQLQN | TAAELLM(+15.99)KGADPA | QSAAETFKSGNN | LMFYDEM(+15.99)MV |
| TPDMYVNFNG | KNKGTPSVLYN | SLRTC(+57.02)LN | LSLLTLLHM(+15.99)SMLPH | NSQHAVM(+15.99)TAC(+57.02)A | LTTGNC(+57.02)SEDDKSWLQR | S(+42.01)GTVFSLYVPQ | SSPDLFETSMQ | NC(+57.02)QGNAVLVAAGPNTLLGMF | TVEAPSLK |
| LFGSNL | QDSHKAAGAPANNEC(+57.02)Q | FHSEFEELNR | KFDDHVFNYT | RFAVDKLYLPSE | KNAFFEALLY | TGKLRPAT | EPPVVVPDDC(+57.02)GSHVMPQ | TPDPGFPGT | SESYYKLFL |
| TKPVAFSPGASKS | TLRPVAAQ | KGC(+57.02)DDEMGSPR | DPNELAQNNLR | QLTVKFETM(+15.99)ALE | TGC(+57.02)KWT | APVSSLPGK | E(+42.01)SKTKKKQKEELEESC(+57.02)REEK | VVSHGADYEM(+15.99)SAR | LPLLPVLKE |
| SDNEFYV | VSGNQPALAETAPSGHS | FSGHTLPWGRLP | VFWHLYFQ | TPRVVVSF | KAPAYMPPGGTFAQ | LLNTSGC(+57.02)GMMSLLVC(+57.02)RPR | KGVFLNADSVT | TLGSFMLGTAM(+15.99)EK | THSSLVKFM(+15.99)SE |
| THSLVFVKFP | F(+42.01)SELNKVLKMEET | TVM(+15.99)SPQHPNL | SGHDVDLK | ESRVWKETPAAH | SPQDDGPF | EGGGALVVQDDHT | LENPDKLLQLL | TPNGM(+15.99)HPELK | LLDTMLNAAKTGPGMSTPPQ |
| ADAALFDWLQEQGVL | TM(+15.99)LKVGL | TDSFNPSQ | KVAFNPPKLPH | LAENHQT | PPSALLAAYPNGPQ | SHKKTTL | TVNHDLM(+15.99)HR | TVVAATKPAAEKPQ | DPLNQSEELEEGAKKKK |
| TPAKLT | LPYEVHTVFSNSSVT | TKPLMNN | TAGPRSATQGTY | M(+15.99)DMGGGAVLLTKLAGSSLEDG | TTDGPNAKPSGGL | T(+42.01)QVNALVRVVFEPMVVPQ | LAGSQDNVLSRF | TVRAMYNGRGA | TPGAKFLN |
| T(+42.01)VYSTMGMLNKVLFSR | S(+42.01)QAPGFVGADFN | TPGKSGPALFFGPGT | WGLEFK | TVPLGGKAAC(+57.02)LGPP | KTTHFLVQGAQ | ENNYMDTRGVA | KDSPFKSSLPTAQ | HFVLL | PVAKPATAF |
| VVNPDSSSKTGGH | SPAKYR | EVSGTLGETLEQR | AVYAKLSN | TSSEPLMNGPQ | FSLYLN | SFLWTHDPAK | P(+42.01)PVVFLR | TGKASHH | KDAGSSGFYHPS |
| LADHDANK | TGFFSHGGA | TGFQNGGVT | DPAVMKTDKGDLPYVPQAWEFGHL | EAAYEKNVLATQ | HLVFVL | KRFTDS | KNLEPVKMPN | THYPSSNKAPA | TAPKKLPF |
| TVSFHPTLLER | TSAGVFLDRATLQ | TNVAVLSM(+15.99)SKPH | SVDYAPK | AHDAPPQALSM(+15.99)LPSQ | TKKKVSPQ | TLGPGVFASPTL | KFGKELEQSQNDPE | VLVADMGMNNQHGKVMLK | VHLSSSAC(+57.02)LTGDSPNYK |
| APLTEVH | TGVLKNMN | VGQDLQSK | PGSAFESGTLQQQGTWGSAQ | TLSPAHML | SSLGNLPSGSNKLQ | TDGGHQYRVC(+57.02) | AAAPESQQGSKVPR | TAEQKLANYL | LVEVPDSSDSYR |
| TLPEFSMQLF | LQVDYC(+57.02)A | NSEQLAAALSR | TTAM(+15.99)RTSYGAQ | KTEVAVM(+15.99)VELPTVDLGASK | DPEWAPYRAY | SLNSTTPQ | EAGLKMAC(+57.02)WHT | TPKVSNLK | TVM(+15.99)GSAPAEC(+57.02)RL |
| VVDDFFDVHQ | DAGAGFNKGELQENVL | TMAKEER | TDKNNC(+57.02)FLES | SQPVAGEPPMFDYFL | RAAEAAVHLSPYSPDVVSF | FVLGEPM(+15.99)WYN | KGPVVTDLPAPH | FVAFRYGGC(+57.02)PQ | KLLLLK |
| TLLSPQY | DPVMTTNFEYVSFK | KGEGSTTQEGGVL | TQGLNVGSH | LQVTM(+15.99)DDEGRVFDADLQEARVV | TTKKHM(+15.99)LSPK | FGGGNQEFLGGVK | DDDSKSC(+57.02)C(+57.02)PDQC(+57.02)AC(+57.02)TK | KNADTNSFEYV | NTARSDNFEYVSFK |
| LVELVS | TVSSRDDGSNL | TVGGDLAEK | SDM(+15.99)M(+15.99)M(+15.99)YTSF | TTC(+57.02)DM(+15.99)M(+15.99)NGMR | GKPPVPH | SAGKNNLP | SAGKNVPQ | KQRGTGLPYSDK | VTGTVQGKTKEVGQDLQSK |
| NLRVT | TPQRLTL | TFFVGGNNPGLP | S(+42.01)YGGQPKKPVTGWPGAL | KNGELFMSVAVP | S(+42.01)QGWYC(+57.02)NGPAK | TGDC(+57.02)DDDSAPGPV | TVLPTQR | TGGEDAAYSAPHSEDLAELK | LALQELPS |
| LVVEFNYVP | KDSGHTVR | LFVLPA | TAAFLHGMVK | TNTPVGPGKATF | SVGC(+57.02)M(+15.99)QNP | WAGAGGC(+57.02)AVFDGELQEGQVL | KRTEVAAQRKTLQP | PNPAM(+15.99)NPETKY | TSM(+15.99)YGPV |
| TVANLLPAAQSLGAH | AHGYSSEEHC(+57.02)APAHPFEWKHR | TNPQLLTKVPH | TGNGWHPE | TM(+15.99)GSVLK | SC(+57.02)KGLVPL | SVNSKGHDAAPGP | VSGEPKKVVPPHS | LAEGFTHDPSQPLEKVR | KDC(+57.02)ETFFLSL |
| EAGKGFNGELLPPLVEPV | KDGGVLWAADPGVQHG | TSFGVMKYVTL | VDPFGNGNPNYSLDDLR | EHNLVLAT | KVARVSC(+57.02)HLLT | TVASKPESS | TLM(+15.99)APLLSSEGL | TAGGLTAFMVPQTQ | TTRKGSH |
| EGPVPPAAYTRQR | KMTLSRHDESP | KGYQTTQLDPDAAAAR | EPHTSTPVAPPAP | KLHELLPMPS | LRDC(+57.02)SPTLPVQR | TLEVAPPQKEGVR | NAVAEDNFRQN | FNVNHLKAHYGVAAETH | TVPDRGNMNPFY |
| TGLAVVPT | KSQM(+15.99)NSLLAVPT | TVHM(+15.99)LPPGSVR | VTDVDDPPLTTVFFN | KNPDTPHT | TSVM(+15.99)QAAGPGSNP | KPHWLT | TLAHGKELLVNFP | EHGGPRDDM(+15.99)YPRAYK | SDGGGLRGAV |
| QVPVLVDLDGNH | TC(+57.02)MLK | TVGPFKLNSSSN | WSTQLTGLGFGH | APGC(+57.02)GHEPVNAQDRGGPPG | TVPKLSTAP | TRFDFF | TLLAC(+57.02)KPLEVPH | KDLFQP | SFRLQP |
| NGNQSLLEVC(+57.02)L | KNKPLPH | SLEAFTVN | QGTPLDVAP | SEPLKRS | PEKGTYPSLHFEMVKSH | TAKELRK | L(+42.01)THLSM(+15.99) | S(+42.01)HEDLSM(+15.99)VNM | ALTDNNKLLML |
| TLFEQYRGVQ | EAGNGQEFLKYQ | PDGAGVYPEVMLQ | KGVNDKL | KNGHSLVTPPHS | NLENLFSKY | SPPVC(+57.02)GWT | KSQGKMPAGLLVSSH | KGHTDFEDLYNYK | S(+42.01)GTTHQEVYLGE |
| TDDDLAYPKHVGP | TVGDKFDAVGGNP | KDFNVGT | DELTSVLGEVDEMLAER | KTNPPHS | TLRDTQLSNH | TTYFLLRQN | TGGASWC(+57.02)PHYSSK | EQPPVLTM(+15.99)SQQ | EAVGNGDFAKFN |
| TGDLFAALESGGY | AHGATEPGGSPDPPK | SHLDPTF | QEQEFLKYL | TLAGLFKLSGAQ | TNPPSLAAYSF | TLM(+15.99)KGKSVPTY | TLPPPKSH | KAETTGAPLDPPAAEGGY | QPDLEGHPQAGF |
| TSHKTFR | KGM(+15.99)QKKLLYPSEVLAH | KDLAGVGPTPHGL | A(+42.01)NSNFPK | N(+42.01)SLFNNDQNRKNT | ALVTAAGPHGPKNMMWPK | QPRVAAHQRAN | KNRNTDC(+57.02)YSF | KDDQLMAFHT | TTKNSFVT |
| TGDKLM(+15.99)PAPAH | APNKGNLSP | TVGAAHKFVRPQ | S(+42.01)NWLKDLEAY | MAFVYNHLPN | PHHADADLLLFL | SFC(+57.02)RLN | LSGGSGNKTNMSGTGNLADAK | TVVDYHQ | SKEM(+15.99)VTP |
| M(+42.01)WMC(+57.02)SSGLYLKAC(+57.02) | NC(+57.02)C(+57.02)SSDRDSYR | TLLPVLDKLEA | TQPGHNM(+15.99)NPSQ | TVHLSYM(+15.99)VPLK | HLAPPWKTGAP | LVSGAPC(+57.02)C(+57.02)PC(+57.02)EP | LEDHTGHS | FNTDEADTELK | LVEHNLAL |
| QVNNDPETDAPK | TVSKYNPG | TDQEKVL | A(+42.01)AAKLPPLELKVL | TSPDYKSP | TPFSSSM | TVGYLDKADFH | EPGRVQDHSLK | DPDMSAQDLM(+15.99)T | S(+42.01)VNNDGVLSRS |
| FRTPNHLVAM(+15.99)T | ERQDEFM(+15.99)GHDEDKPRKDRAAAPQ | TLNPGVNGL | QGVKLC(+57.02)R | SALHFSR | LAEDGYSVGEVR | SFDELKKPH | ATALGREKADDAGGDKWHR | ALQPDTGHKKTHAAHETK | TVGARALVEEKPQ |
| QNPPVKQP | TNFC(+57.02)DPVAELK | KGGQAAAVSL | TMHHPC(+57.02)KGRR | KAANDKLENNPFYLR | EPC(+57.02)KKVPEE | LAEPDMKVYSE | NGKRQTLPYSN | SSYKPK | TM(+15.99)RVTQ |
| AYLHLLF | KNEGMNFGVTL | TDELADHQDPV | NSGLRLEYPQK | KAM(+15.99)DC(+57.02)LESQQVLF | KDHPAVVNGASGF | LVTVTPANEAVK | FHDDTVDM(+15.99)TNC(+57.02)MC(+57.02) | SPGAEAVEKKPGGSVPTTALT | TLAMTDKALAFPQ |
| LHRDYPYPR | GPLVYGMP | TVM(+15.99)MERRSL | TGGGSEKFL | KGFNFF | PQPKYLR | TAALNQGNYPF | G(+42.01)KGFALANPPSQ | LVDHNMDPQPNT | TNPLNFAH |
| KDPSEYLYNP | SSKTC(+57.02)PT | TLLEEQVRNN | PTVVGGAM(+15.99)SKDPVC(+57.02)HW | SVGVLTNAK | TPFADKSPAPLAL | NTDGEVPVALSK | LHGPPLVTKAKH | TSPPKKTGDKS | TGSPRVFQ |
| K(+42.01)VQC(+57.02)MEAVTC(+57.02)R | SMVM(+15.99)FVA | SPELVLSN | NGPVTHSSDAAGN | TVELKLPT | NALNSFEPKLQH | ELFFLSSTLLQ | SLKPWLT | SSPPYLSN | TGTVYGEC(+57.02)VSSNK |
| G(+42.01)KALEC(+57.02)YVVPV | TC(+57.02)SHKLPALK | ALEPVLTNM(+15.99)QPDDSLY | TVLLLQQKYN | TVRRDPLYSNR | G(+42.01)KNVPC(+57.02) | EPEVKELTTHT | TVNM(+15.99)LSSPTY | FPVLVQ | KQETTDVLMN |
| TLFGSYGPA | TVNVSC(+57.02)HFGA | K(+42.01)DETLSEFGQ | T(+42.01)VLEDPC(+57.02)PVQ | VLLVQPN | KQAAQAQDQEQC(+57.02)LQK | APLAVGC(+57.02)QQAVGN | PQEALFSLRWP | Q(+42.01)LSFMGQQQGEAGLRQ | KDVRFTTLGA |
| KLTTHPVEGLQP | KADGDC(+57.02)PLLLY | LVDLNGNH | TLFPASDQTY | AHLLFVGL | KNGLLTSFPGTA | KDAYC(+57.02)C(+57.02)PLTTK | KGPVVNLM(+15.99)SET | TGVFPSLT | TLMGHNAGAPTF |
| EVLGATHKVAGL | TEEM(+15.99)DLLKTF | KLLSVSFP | KALLSPS | EGHRYNTGGQ | GVALC(+57.02)DK | TVGNVPGAFKPY | TGSDVPVSSLLPH | TLTLQPNPGPAGQ | AEEVAQFEALTEVVGDHQ |
| QPRFNGDEPFMYK | TVLM(+15.99)ADAM(+15.99)ATLK | TDKC(+57.02)LRPR | TDYHLKAA | G(+42.01)KAPLEE | HSSSKDHGASSPSYHR | DFVSC(+57.02)C(+57.02)SLGLP | TLGHM(+15.99)KDYTA | QSPVQM(+15.99)LAASLVSHSVEQ | KFNNTK |
| KNPVTATMM(+15.99)LT | LLNVKRQSF | TGPSKLEA | TGQQESKPAAH | RKDNPESTVPM(+15.99)DVYLPR | V(+42.01)TFLELH | HLGATTSGGHYVLK | TVKLDSENNQ | K(+42.01)GVNPGM(+15.99)KPGVL | TVGDTANQHTY |
| TVDKM(+15.99)GDPSSAGAP | STHLLLS | TQSDDPL | MGVEPRQSTERL | KTFEEDYLNKVLFGR | ALLFPH | TLMMDNYGKAF | EGHPGDMGVSPAK | TGPHM(+15.99)LAGHLTGP | QVAKGPTPSLGAH |
| TVDVVLHDPRGPQ | KDGTAHLDHLN | KLDNYTNGTLK | TMLPKPVS | TFVDAQPK | TFELYGAAFL | TDESSTLDYEVN | KQHVDQEAAALPH | TPNDNMVK | FLYDDC(+57.02)GKPR |
| SVLKTPAY | GVAAPEQHLPTW | G(+42.01)LRRTPKAH | KTEKYM(+15.99)HSSK | LHEGAVAESDDYKVPHGP | QPSRYTEDLNS | TLKNLDKPF | QAGC(+57.02)HQQAGAGY | TLLLPH | KAM(+15.99)PQNGLVLL |
| ALGGTVVGNESKY | TVAHDKSNGSL | LVVELSKK | KDDEM(+15.99)M(+15.99)SPVVA | APDDSLM(+15.99)P | TLEGATTSLLAVPGT | S(+42.01)PELNALLVLQ | SC(+57.02)KLTKF | S(+42.01)GADNPGDPKPGF | TVPGHEKLQNQ |
| SAC(+57.02)FNWVPL | KDNTFSC(+57.02)QVQ | SFDPTSR | ATGPVPVLSVVKTE | GHEDKDKNSVQEMLKR | TNYM(+15.99)VLP | ALQDQPTPVR | TLLFSPQ | TDFTKVS | LLVPQGTTVAAPQ |
| PVTKESGGLETTLLATTGGH | A(+42.01)SDGSAKNYPT | PDLM(+15.99)TELTPRLEYANHA | TVGKPEQVVSAKY | TAPAKLHFAH | LVDLDGNH | APNALKPAN | SEPMLGTL | KNLVTTM(+15.99)VPQ | SSHGEQPLEVR |
| TNESLVL | DEVVLPEGKGGKVMAH | TGGVAAGHTEAGYGK | TAASVKGDGPVRQ | TKDFEELNK | TGALNVGEQ | TALNYSSF | SM(+15.99)ENSTLLVPH | TGAQNLSAQPN | TGVGFDK |
| LLAADESTGTLGKR | FGADLFAPSPGGTVP | TPNAEVGL | HSSTAPAGHKLR | KC(+57.02)SSYH | TGGAAPGPFAATC(+57.02)Q | TDNM(+15.99)PLTTMR | THM(+15.99)KATVLVSL | TLFLETHKNT | TVALPMLNGPTL |
| TPSHNQDTM(+15.99)R | KGADNLVSS | LDVATFGRFKN | LKELEEK | FGSVTKLTYPAG | FLWYGR | LAVYSTGS | SFGLTER | SPEMSTTVYSGV | TVAHLPDHSPY |
| TVGYNMLGGLF | GPFLVTK | TGALLVNDDVQ | P(+42.01)PPPQTL | LLVPPQAPLPPSH | TVC(+57.02)GGNLK | TTTLGKVN | QYVGKQDM(+15.99)MGPVVT | TDKKPAGGTSVRQ | VLLTGVVEPQST |
| TPHYNNR | SAGNLHQ | WGGDHGLHVM(+15.99)MT | TPSKNLNVPTF | TLSGELLK | QPAQFAASTKGAP | KGLLPSVS | TLNDFFGA | ESAC(+57.02)FTVAAPDQNFVRPY | SENRVSLKTN |
| TGNPSKFR | TVGGAQEGAKWVT | KGLKAGPV | TVNEVAPLFHRGT | TTGPEQELVAV | TPESRVFC(+57.02)H | LAFAAVC(+57.02)KM(+15.99)MGPL | LHESTPAK | DPNDSPKENLM(+15.99)R | QAGAENQC(+57.02)LQ |
| N(+42.01)SLDVPC(+57.02)WVEFK | PVLQPQPLPPHS | HYRC(+57.02)SAVVM(+15.99)MT | TQLYTFGSPSQ | TMMNLWQSY | KPFKAAPHGKT | KMGDWHPELK | TPVSFFLTML | R(+42.01)M(+15.99)TLM(+15.99)EPFSDVVNDEGR | ALGTVATAGDRVTDLTPGAGR |
| A(+42.01)KNVPC(+57.02) | SLVVAC(+57.02)C(+57.02)MLT | TDPEMKNSPAP | EDAASTLTGKGR | SPETVLYHGSP | KGGNSLTEGFKGHEDPQ | FPRMRVFRLATM(+15.99)KEY | QPPKYLR | TLPVVHR | QNPPATGQEAEAGP |
| SNVLNPAL | S(+42.01)LHSSPYPFPQ | S(+42.01)LLAWPVM(+15.99)AQ | TVFGPGPAVVC(+57.02)GM | TADLFLTK | TVSALTGFP | KDNNQVPWSRVTR | PVVVLEVNGNH | T(+42.01)TVLWHSPC(+57.02)PPHS | TLM(+15.99)AENQELT |
| VPGLQLPY | TVFAARALLYPSE | KQGREGLSFKVE | TRYVFR | KFSPAK | QGGPPEKLSNAA | TGGPQLVVTM(+15.99)DGL | HLSPYSPYM(+15.99)PR | TVGAMQNSYTT | S(+42.01)AALLPVVYGAA |
| KLLDM(+15.99)T | KDVFLLNPDT | AGGLLHGALPMM(+15.99)T | VGGSTVC(+57.02)KLVC(+57.02)YSW | YADRVSAGPKPQE | LKEQQQGEEHAVQLQGKA | LLLNPH | SNLVSFAL | TVGM(+15.99)HGELHMEK | FDL |
| SLAHVVAP | TVEGM(+15.99)TLQQSY | K(+42.01)DPDKTHNPSQP | TQLEPPHS | TQLEPPSH | SAFTYAL | TPGHLYYPEF | EGGTGRGGVTDLGQK | LTEAPDKFNAQ | SDYMKSF |
| HNLGQTV | VPENQSFSELEEKQKKK | TVNPMLLL | LLGFKLNS | KGPPTGPNHFSM(+15.99) | TVMDDVLNGA | KDLFLTH | TFM(+15.99)M(+15.99)M(+15.99)MFT | TLGVELGAK | QSLTEM(+15.99)C(+57.02)GC(+57.02)ADQLEGR |
| SDVVLPQ | KDFQLTTTNP | TVLAKNYSSTL | SFVHVSGL | DRGGPNWTSDATR | SVSPSGQLLVLSS | Q(+42.01)SLESVDC(+57.02)C(+57.02)EDMVEDNP | TGNTTPGKPAAH | TVDKPDTN | TDGNSKTL |
| TESLLRQ | KANC(+57.02)DVGA | ALNQPPTFGM | A(+42.01)AEVSVPDKHT | SRTPVVAL | P(+42.01)SPDGTPGVC(+57.02)TTVDKFGGH | EPPGNPGFPDLEHPMTM | KNGM(+15.99)TGLALVPAT | KGGLASM(+15.99)GALVAPT | LEGLGKRFQHT |
| S(+42.01)VGHLLC(+57.02)KTDQ | C(+57.02)VDFSYT | TQKNPPHS | TVGPAQC(+57.02)L | KDVYEFADGPAK | APLDHLLFANGFPYELK | VGALVSSDPAKSLPYHLQ | KAGDGENVSGADKKMPVN | LASENMLALTY | A(+42.01)KESLVVE |
| TVYHEFP | EAATGGPHSHVDLK | LLASNFAT | SVDPLT | KDEQNAH | TQLGKNFRPF | TSVQLPGL | THAVSPLYTTH | EQAADPLEKVR | VVSHAM(+15.99)T |
| DQGPPRLPGNHL | TGWFKDAAAH | NALGPTVRGQQ | VPPVTTLVPPGYGM | LAVKLTTMTHLY | SGLKNM(+15.99)TP | ELSELPHTPVQ | S(+42.01)PVKVPNYLHT | AGPGAEEPAGAGRGQ | ALGTMHDPLSAAN |
| SLM(+15.99)RNVT | A(+42.01)GPGAC(+57.02)RFQC(+57.02) | SVDDKGPL | TPKDLLGC(+57.02) | DHLAFHTVPDQQ | APDAGHASVYMMSFK | N(+42.01)SQEPAFAFLPH | TVPM(+15.99)M(+15.99)SNTLK | TVLLRPN | QNPPFKTLPVGAP |
| TDPSVM(+15.99)TQYP | KC(+57.02)DLRN | TLDNKVPSSF | SHHLVTKTHP | LAGVLC(+57.02)DPNGPATK | KLVTPPSVPHAEK | HEGKFENLARPS | TVEVPPALVVST | VSALPTDPC(+57.02)C(+57.02)NLHKPDAH | VASLTEAKMSEELNKTK |
| PNAAHPSTHQPTAP | KPVDVMT | SPNNPTGDDLNK | KFNQYKVPSH | WTPALTVM(+15.99)PELK | SYKKDDHMM(+15.99)S | SHFTVPNL | KSSELYM(+15.99)PH | LLVPPM(+15.99)VVERQP | HALLYQPVDHQADAF |
| TDAFTTC(+57.02)C(+57.02)QP | KNYDLHSLM(+15.99)TSGWQR | TPSLKYP | LPLLVLQ | LLPRLDGM(+15.99)SSST | KC(+57.02)TPHH | G(+42.01)VGSPRVAAEVALLKMGQPAS | SPVKVGP | TGVQAEKL | RWTKELWMMVVGWDC(+57.02)S |
| TAPMDGLAKSVQ | TPGQLAHQRLS | SVKPPSL | TVM(+15.99)SSSDP | FNAELMGPSTY | GYETLGLFKVYGLGH | SC(+57.02)FNLN | ALLEALPSRSL | TVGVDLPGK | ALDPLTPVVATALC(+57.02)PPAEGP |
| TPKNLTC(+57.02)M(+15.99)TM | HDDLDRSAR | SNSYTPK | KLAAEQMEM(+15.99)LT | TC(+57.02)EGDDN | PAGGGFVFSLKKAVLYGPH | TVAC(+57.02)DM(+15.99)NKYL | ASDLSKTLLDTSLHQ | KAGSNVATTSLPGAP | KKATPDHHPKT |
| VTDLLTAF | SFSVAAFL | KANEPHS | TAVMSQLN | LLVFC(+57.02)PQ | G(+42.01)KGSPVALTPTY | QVSC(+57.02)HSADC(+57.02)RC(+57.02) | HYYDWGTLMDWQLEGR | TC(+57.02)C(+57.02)LDC(+57.02)S | HGALPVTPN |
| TVSHKTVLSNL | SPGALFNVT | NFYPSDGYSMV | KNLVKSPVLVAG | KDSM(+15.99)PH | TDEGKEEC(+57.02)LL | SLGPLVGAM | FLLLPH | FLVEEAAPQSF | AAVGGMRYNNMT |
| KLLETC(+57.02)GDDGAV | G(+42.01)KAHHYHPPSH | SFAVTPML | TEALLVGGQ | KPLVEMGPAVSL | EPHNTLQ | RFQKSTP | PTKPPGAVPMLST | TDAALRPASEN | QGGEFYGGPDLPK |
| THYVAANGTPAAL | K(+42.01)GPAPSDDETNN | TDFNKVP | KNHGLKTFPK | AAPGTTTTGVGLSVVDLQSK | NQPPETYYLGGK | SPQYLLKLDTPV | KLYC(+57.02)FDPH | STAM(+15.99)PQSEELEEKQKKK | DLGGAHC(+57.02)NEPRVLDL |
| HLQVAKGESTPQ | G(+42.01)KSAPLTSEEPH | SLPPVTVT | TLFLEMARMST | QNGAESSMVLKYQ | TVRPSKFA | QLVPAPHYKTPS | SLAKGYF | SFVSGHPT | KLWLS |
| TLEM(+15.99)VSKYAPL | QPPTSSSKLVTQSFASQ | SFPDTKR | TLAPASC(+57.02)DTKF | SLSKTLLYPPGA | GPEDFKKPVGAV | KDALEFLALSDK | QPGASHGPQPQQR | TGEPSVWVVK | KSWQHQ |
| SLAENTPK | TLAAVAFTQAEGK | TKLLTPK | SSSVAESNAAVNT | HVPPSSTQELDLLAGSPM(+15.99) | QGVAAVTKHMFLQ | EHGGLLATDDDYPYRAK | TVSAKEALP | LVVELSR | Q(+42.01)TVQKLL |
| TLAGGPDAVC(+57.02)KTR | QSFLLTPNSQF | C(+57.02)DVVLLN | TGGGHSNPKGRVAT | TVSGPKTK | SLGAHFMT | KSYTKLLFDT | EGSRVVLPHSGGP | TLEPM(+15.99)YH | KTDKVGQDLAGTR |
| EPEVALLQ | TVEALDPPDAKASVGW | TGGRGKKKKSDSGP | PSGETM(+15.99)MLSTKP | S(+42.01)GGVFHLAPVLN | KNHEFEELPEMT | TLGPAAGSPGPGTLHL | TVHSPYF | KGGAANESQVGLK | GHAKTM(+15.99)T |
| KLPDQFFRL | TVDKAHVAKGLVF | GPGAASTRMKAGPPVTAQ | SVM(+15.99)DKLT | HLAAVEPQ | TPRNLLF | KNMDC(+57.02)EVLAST | HNASSVY | PAKNVY | SLRVFG |
| GHEALNNNSVADGVTH | TPAKLLPA | TLPM(+15.99)KDWDQ | TGGGPPTVGDALPGW | SLVTMFAN | SFRVPH | S(+42.01)TNEALGWFN | TPGDHGSH | TTDPNMGQPLRL | S(+42.01)LLVLVQP |
| TVNHQSLTSVSPK | SMDVTPH | TVC(+57.02)KSSMLTY | TLEDLLPNHGSLH | TVGSDDDPAGHSK | TPAEKASLGGPV | KGLEAM(+15.99)DM(+15.99)YP | TTMATPGGKGGESTT | QNNFNLDQNPR | TVPFARGK |
| RLLLTL | KPNTLVGPEFPQ | KSKTAAPGLPGSK | KGGSEVAGNSTVAR | KPGMTFF | SFLMPK | LVPLQRN | EVLPSGGH | TVC(+57.02)PPLAGA | TGEGC(+57.02)KSSSM(+15.99)N |
| TSVSERT | EVPAQGSFDDET | KNLVLM(+15.99)SSTVP | DPVSAGKC(+57.02)YYVAAGSF | G(+42.01)KNLPC(+57.02) | TEAGKLVSV | TPGANDLVK | TVAQNSSGK | TLFAAC(+57.02)C(+57.02)HGQ | EDTYLETPHQQVM(+15.99)P |
| TVHGRNDRFTGK | LLLLPH | SETTNVY | AMDFLGAGT | GERKEHDHPVVDYDVY | TRGGALVVD | TGDLADKLRFES | K(+42.01)SC(+57.02)EVGLGALDDDLGRLR | TNNSVMF | TPASKLK |
| SRGGLLEASYDTK | QPDTTVFNVPY | KSYVLLG | HLVFVL | AFLNANGAK | GAPADPRTSEAC(+57.02)SSLQ | TVDKGLVPGYF | LLPLPVLK | LHYNSH | EPNLDVAASGSLK |
| LVAGNDQELLE | S(+42.01)GFPKSYALAGGP | TLPPEVVVGM(+15.99)TS | QDLLM(+15.99)M(+15.99)M(+15.99)PNK | TFDVVAGQWGVT | SNKHVFG | SLGYFMKPQAP | TFAERANQTT | KDC(+57.02)GGALDENKVK | TFLVYSGN |
| S(+42.01)LTTHASYKMQ | TGRC(+57.02)HQYM(+15.99) | TVEEPLHS | VTGVEVEPKSEELNKV | C(+57.02)GDLLL | TVC(+57.02)RVEDVKVPH | SLGNLLKAANL | S(+42.01)SSLSGKPPQVPAG | SC(+57.02)C(+57.02)YKP | SLAERLN |
| TVC(+57.02)DSATKLVP | TQAEKKKLVN | SVDNLTH | TEMSVAF | LGGLVQPPEGKAAAP | TFSPNLR | TFKRGMP | TPKPLSGF | DGDLLVLVGKNTVP | EPQATDDYFTP |
| TSAESPLTL | KSWQHQ | HLLPH | SVLYVT | TLVPDGGAPKLNT | KHSVDNYLTLPEHH | K(+42.01)DETLDKNGVP | SLKPTRGG | SNNTQALL | KNAAPVLST |
| TVFKPVMA | TLNRSLLEHGL | VLEDQNNFPKVGL | KLEEEKEEQEVKKKKK | TPDDESPK | KPQTPN | APAWMLLHDAPGVA | KNFGKEYVGQK | SLDMFSK | SAPTVYLT |
| D(+42.01)TLFDKVLFGR | TVFKTAGDDVPF | AGVGFVLTSC(+57.02)SSQ | TRSSSC(+57.02)Q | TVDDPGRN | TYM(+15.99)LTVKFFDY | LLVQAEM(+15.99)YQPA | G(+42.01)SGGPTLTN | KAAGPPM(+15.99)P | TVVPGAM(+15.99)KVPHTSL |
| ALLPVNKERPGF | TVDHHLSENQ | TLDSSFY | TVVM(+15.99)TLADSF | KDNAAFC(+57.02)PTVL | GTAKLPHSP | TLGM(+15.99)VTATPDVGQ | KDLVH | TLNNLLK | AGAGPVDAQKEEGSKGR |
| GHEQSLVESVGGK | TVAGVLDDKYDGP | EAKGPAPVTAAGGTK | SNFALPH | TVAKFTEETLKPS | TVGAPM(+15.99)LAK | DQGAATVPK | FGSAFKNYLTQ | TVLENSAP | TAVDNPPGL |
| LEAGPVPM(+15.99)APLPN | LTGNPDLGFAASPQ | TPKVMEH | TVPKPHEMMLFL | EPLC(+57.02)M(+15.99)AGPVVLVTC(+57.02)K | LVPKPVAAAMTPK | TGVDALHAL | KGGETPSQ | KEGHWQSFLQK | TPPVFST |
| TVPPEPMPGAKSK | A(+42.01)KHVNF | TADLGC(+57.02)EPRN | KEKDRRFLPNEGH | TLESVTAAQQTL | KGPYETNSPAGVP | TEALVSHG | TVEKPLC(+57.02)GDL | SGTDLVPLN | HLSPAFTP |
| TPAASEVSLGRPHN | ALKPVTY | TTEHFDPAGSPQ | DAGMMLFEQTTLVPPATW | TVKKLYNDT | AFDLVKKYAT | SDNYLFE | TVSC(+57.02)C(+57.02)MF | KKHLFN | ANLQAGSYC(+57.02)VAQ |
| NNELSAPKLTL | TAAHMSKYPK | A(+42.01)AATFSTHVTS | TLFKPWQ | TGGQPTM(+15.99)DGWFR | KGNAEEFQLQ | S(+42.01)FGGSYKVVC(+57.02)Q | GNSGM(+15.99)SSLLLLYVSQ | TNPM(+15.99)M(+15.99)DKMNP | LLPEQLS |
| SDHKSSDSQPSYHR | EPELLLFKYN | TLAGMGPQNAGT | LVQERAH | KGGQRTSRPPHS | S(+42.01)LAFQKKSTQ | TGANGKGYADPV | SPADHQLT | KVDSATLDPSKQ | TLLNMVLL |
| TNDGDLPVA | HADADHPLLLY | TLVKYAPGPSLEN | SEGTSKKMMGKPQNQGGK | TSFEGKVP | SDPAALVT | V(+42.01)MVLLTF | TAAPMDKGS | TPGNSLGKVPLT | KDVLAVGNP |
| TLKSTVGGGA | TDDASMAKHLN | EPDLLEGVTLGAK | SPDLSTC(+57.02)PPAPQ | SATKKWT | QPHTSKYNTNP | KATPVSTL | S(+42.01)KMFM(+15.99)WNEATLPAK | TLLAHM(+15.99)LT | TAAGPPKDPVPVP |
| TGFAFAYT | LVVLQPQ | PQVKSKGE | PVGLMMAEDPEAAESVPFY | P(+42.01)SVLLAPEVQH | SLEAKSVP | PVPKKTK | TVGKNSEVSHGF | QELLVEPLGHDR | KPFTLNM(+15.99)SVHGV |
| KAAGGSTKTSEDPFNLR | TWEQVY | TYHM(+15.99)HNKYV | LHC(+57.02)PPSP | APGKTTNPAG | KAMDVK | STLKKKTG | KNATPNALPVVGASP | TC(+57.02)DSHLP | TFNC(+57.02)ESM(+15.99)GMR |
| KSM(+15.99)DHT | KFTKKK | S(+42.01)VPHYM(+15.99)GNPVQ | GHRNTVFGGGPVAVVEKY | SC(+57.02)GSKKSC(+57.02) | PDTLRNDEGGLKALLETR | TFM(+15.99)LKVA | PSAKEFTAPAVPPGEPPAGAP | ELAAATM(+15.99)QVDPA | TLM(+15.99)PVPSATPK |
| TGRLLPTL | TGLSSWLQ | HLAPEPLLRLF | KPAGC(+57.02)M(+15.99)ENYPH | V(+42.01)TYVVFK | TLPFTVT | SQDTVVTL | TGFLGKGSQ | KGPETTEEMSPG | KNLVLDPPHAT |
| FVVQAEA | LSGTDPLDAFGPGLSTPFPH | TVLGAPAMSEKPGGP | LAGPGKDVEYVF | KSLLQEFMLVP | TFELPEK | ESHFEELNRVL | TAEKPNELVVF | TTGLEAKSLFLAT | KSAATLVGAP |
| LVEGHTTSLEFK | TAANFLKS | ENASAVENLLKNQ | TLAHLGEPLLNGL | KAAQTVKHAKSS | SKEPPHS | TVPKDLMV | LYSVVTK | TVGAFLAKYFGRP | KNWM(+15.99)QEEQEWKN |
| EGDLLKAL | TVLDDKGATASVR | TGC(+57.02)KNPVP | TVLESLK | SLDTKVGT | SALHPHR | TLDQM(+15.99)YL | TAVAGPTKDYGTGR | KVPDYGDT | TQVGHHFTLK |
| SAKTM(+15.99)YP | KPHNSLEQR | TFDHKLRSW | TEM(+15.99)TSLT | QSARGHTEAAHPAK | S(+42.01)PEHMLYKVT | TLFLLK | TGFGC(+57.02)AGTVNPQ | KM(+15.99)GVLLPH | TLGPKNPH |
| TPAGKVPVK | GFDLRGVT | LYLLPPDPQSF | TVAAVGKHKAFC(+57.02)R | TLGFSSLAPVPRL | TVAWFAKC(+57.02)TTGPL | TVGPPTLHAPKAY | N(+42.01)QM(+15.99)DNH | S(+42.01)PLTVHWEKC(+57.02) | TGAAAPQAYVPPSH |
| KAGKVNEKHAKSS | TPTVADGKRALH | SFVNTAK | NPLEPQPVQDKFLTH | VTTKAVEKAKAGPV | KAM(+15.99)RLGTTWVP | S(+42.01)VGGYQGQGAHLLTAPAKTF | HNLFLVL | KLGPDHA | LADM(+15.99)M(+15.99)TPYTL |
| KLKNVPF | HGENALVEWDAGVTH | KAFTMGLLYN | TPAETKPPGVM(+15.99)L | HLLAPTLS | KGM(+15.99)RATFLYELSW | S(+42.01)VPFDGNKAVGFH | TLGPASM(+15.99)TL | TVTNLTPSKWL | TVSGKKGTSNPQ |
| TLLLVAPGL | A(+42.01)AGNGFSSNYKH | QASLPGNL | KGADGSVEAAM(+15.99)QTH | TVFSGDNELDPV | LAGKTKYK | TFRAFAP | TVKSFFGVAPT | AKPSQWGGESGM(+15.99)SHWVTK | QPDKFLLPYSGGK |
| SEPPTHTT | TTPVSRKN | TELKRPSLAGL | TGFFKLMFAGR | M(+15.99)DGALLTSNSTP | TVRSENF | N(+42.01)SGALDFETVPKN | KM(+15.99)MVPH | TVALLEQWKVS | TVPQGYALGGFKAQ |
| G(+42.01)KLLDEKNQ | HVDTF | S(+42.01)VSYAFHHC(+57.02)YRAQ | TM(+15.99)NNSPAAVEKH | TVGSSEVLKFPK | TQNLFPPP | KEVKYGTPTKQ | TVGM(+15.99)NQPK | KNPAVTLDMAC(+57.02) | TVALDSHNKSV |
| K(+42.01)SQTTSPRATGRNETTPA | M(+15.99)TSVKLLDHFVSGSS | SVVDVVDL | EDLVTGKNLAVP | TDLENFAAPR | SLAHKKMVLSK | TAPLSPPNWALAAP | TGM(+15.99)NTEVYSF | SAAVKDTL | SPEVDNFPVNGV |
| RESWTSSSQQGF | SVHVFTR | NSLKTTVH | EGRANELTPASL | KAGETLFLQKTH | TSSTHLLP | SAVGTM(+15.99)PH | KLAADHKVRTQ | EC(+57.02)C(+57.02)M(+15.99)KVVLT | TMEHGKPYLE |
| KDNVAAKWANPFYFR | KARFFEAAHGAP | TDELRVF | GSALGMSDGFWEAK | TC(+57.02)M(+15.99)SPY | SYPTEAK | TLAAHDDDDM(+15.99)N | TTGLEHVRLKN | TLSSC(+57.02)ALNHSF | TVALPRAQQSV |
| TLPYSFNANKLS | QPFLAPPSNKLF | TLANDPM(+15.99)QPTK | N(+42.01)SLADTDN | KPKPAGHHKLSE | TVAPQNC(+57.02)YMFR | EPTPSGHKAKPQ | KPLC(+57.02)KDYSQR | SGSGEHNKLLHA | TVMKGNFP |
| TVRVEELFSK | TDNLFPT | TTAKALVTT | TVHSYTVPGAKL | QPVM(+15.99)YKP | G(+42.01)KPVVPLGFDL | KVM(+15.99)VHP | LGVAHLLF | SSSPNNTYSF | WAVLDKL |
| SLPTKANK | TKPVSAKN | TLPSTSLDDVLA | KDTASPPAPAVDM(+15.99)T | KALTSSNAA | K(+42.01)AFNSPELHKSL | TNVFLSLP | TLEDGVPK | SHGKMPTH | TLMDKDGMHY |
| SSSNNLQDQTPR | TESLPKGM(+15.99)DLN | K(+42.01)GGQYTPVNKDVHGAPPF | KMFLLLFTWT | TTEHKLM(+15.99)GPY | SFGVHELTPSSYGPGVSTF | TLPVTKAF | TVQC(+57.02)EPVVFGT | TSADKSVP | KQHVVLGKYN |
| HLLSVPTFFGKPGSN | TLSC(+57.02)KFGNNGL | VSEVAC(+57.02)SSNDAK | GFGYSM(+15.99)STTNAQ | TLAM(+15.99)PVLC(+57.02)ATY | TVLLAMAQPLY | TAASVGGMKPGGSPDDHVTR | HDFVGLL | TGFYVGSAHLVP | TDNLSM(+15.99)M(+15.99)M(+15.99)D |
| KDYEF | TAAGQGTTNENNLR | KGNLFEH | STLLPPK | K(+42.01)DETLM(+15.99)MNR | TVFKGSTLLSY | KDPPGHSSRLHAGR | SQNLETVP | KYTVFMQTR | L(+42.01)M(+15.99)MHPL |
| SAGDFVTPL | SGHKKLS | NQC(+57.02)M(+15.99)ETFPK | FDRGYVPRPTEAP | APQYVLGR | TDGQPFVLC(+57.02)TMR | SAPVLTAC(+57.02) | M(+15.99)TSVFPL | VVVNATTEM(+15.99)M(+15.99)DVTAN | KGWYGSHKTVT |
| VSFTPH | TGPVSKAH | GVPPLTC(+57.02)DPGHLLVGY | TAGAVGKLSSDPS | TGMNVDLK | TEASVVLKC(+57.02)R | QNPPTTVKAYLQ | KKFKH | TVSEHLDP | LSVVNRP |
| DDDTYKYVPQ | TGLAESEC(+57.02)GVLR | QPVTSTPEAGSPR | LAHMANPFTPEK | QPRTVLHYFER | SNWVL | PQPKPH | ASVGEAC(+57.02)DGQC(+57.02)C(+57.02)K | SYC(+57.02)QLY | VGGNDDRDAHTP |
| TM(+15.99)LGYSTL | LLPVPDM(+15.99)T | GLSGVKLVVGGESPNKETH |  |  |  |  |  |  |  |

**Supplemental peptide list 2** Short peptides of NY100

| K(+42.01)DETLDYNR | QLREAQQNEC(+57.02)QLQK | TGAVSKEYKPHG | DPLLTPGHNVNP | DKAALWM(+15.99)YLSPVPT | TNSKPGTDAGLFVLPA | KSHFLAQ | KATTFPKDELM(+15.99)KKPQ | KFLYQ | VGEENLC(+57.02)TMK |
| --- | --- | --- | --- | --- | --- | --- | --- | --- | --- |
| FFEKVALTPE | TFALAAGSNPTL | ALFPDPVTTEK | A(+42.01)QAGAALFVAEK | PQVVKFPVNESF | PVNKLPHVDPGAF | KNVPLKTWGNSF | Q(+42.01)RFGFNQNLDQTVRPF | HC(+57.02)NEGEDKGALVTVK | LDTSPPLM(+15.99)YLDQNFVRP |
| KANGQVYGALLFLFAM(+15.99)T | ALPEEVLQHTFNLK | GVEENLC(+57.02)TLK | LAFPGSAQDKQQ | PGSALFAQDVER | S(+42.01)PVTAVM(+15.99)RVEP | T(+42.01)TVPPHKDAVH | T(+42.01)TVPPHKDAVH | T(+42.01)TVPPHKDAVH | T(+42.01)TVPHDPKAVH |
| TNKPGAHPTLAH | TLQRM(+15.99)VSKGAH | PVNKFWWSLT | LAFPGSADGTKPT | TDESSTLC(+57.02)PSR | TSNDLFNNQLDQNPRVF | TVELVTSNDGMDQTPRVF | Q(+42.01)AAAGDVANGAKFVSHPSGAQ | TANALLE | P(+42.01)SEVLGAVALQH |
| S(+42.01)SVALLC(+57.02)SHLLAVTP | NALLKPDRNLE | QGLC(+57.02)VREDLNPSPKLE | EEPVLALAGHTFNLK | LGDSM(+15.99)TLQDEK | LGTSC(+57.02)LTEDTR | KPGGSSDGMPARRKRREGGLSVLSPK | PGAANPPKLQTHV | VLRNL | G(+42.01)KALKPDQNAAK |
| GPSAVAGELALFPGSAQ | PPGVNYLVGFHVLHNVYP | PPGVFMLVLVQDPGHRPY | PPGVLMMAGDVLVLPGVPPY | LVPNKEGAPEAR | QDAC(+57.02)HGM(+15.99)AWWVTVK | EGGADQEEDSQLVTVK | PVNKALFEGPRSF | PLVKNVQ | KKPLGVPGALM(+15.99)TSF |
| NQLDFQMRPR | TSEAAKHAPPP | KFLVPPGALAM(+15.99)SLP | GPAPAPVGQPPVTLR | KFLVPPQEGTGKR | PVLANKGHTYK | AHESTVVKSSAK | KPAAPYLPYSNK | EHLFAFSVDKQLAKNL | PAGFYFFVDTKPFREGF |
| RESYFVDGAAPKKDASVAN | PQFVLPVVPPC(+57.02) | EGGSKAGPFNLF | PVNKALFAFFDADPSSL | S(+42.01)LHSLKNNNPF | KDVM(+15.99)YNKPAGR | ERGFYNPKAGR | KPGVFKANYAM(+15.99)P | KFGVAKPNYSLD | KFSVFFSSTTLGN |
| TVAPTGSFENLKEVQ | LHKKKFASSM(+15.99) | FSSREQPQQNEC(+57.02)QLQK | PGSAAM(+15.99)WGVER | AFPGSAENLDR | SHDQKELPKPPPVDSHQK | KDYSLHPTLTAKDYR | NQLDFAKDPKE | ALPVNKNMVLE | G(+42.01)KALKM(+15.99)MANQHLVNFLE |
| PDLEM(+15.99)EYPESPQQQ | PDLEDPEGDAFYNPK | EC(+57.02)FDLC(+57.02)RPC(+57.02) | QM(+15.99)RC(+57.02)PDTEGF | SSREQPQQNEC(+57.02)QLQK | AGGLETWYWLPGC(+57.02)LLAG | N(+42.01)SENYSPVVAV | KDM(+15.99)M(+15.99)TPALNNL | EPEVFFGALKFVGAKSQ | KNLVC(+57.02)C(+57.02)FFQTDTVK |
| NPTETFLVL | QMLLVRN | KSRKM(+15.99)TL | HFDLNSLLY | LDTSNFFAC(+57.02)LDQTVGVML | LSENSTYTC(+57.02)LDQNFRPV | NPVPRRDGYKLAM(+15.99)LE | KRTDGAKC(+57.02)KPFNLR | TVGLLFDQKMYDHT | ANLAAPPALKNESGM(+15.99)LLEF |
| KVETTTSFLR | DTSTLNAAEMDQNKKGW | KNPPRNMVKNFVT | KDADNVGTLPRT | ALAQWALAFPGSAENVER | LNALKDPQNNRGNHSALQ | TDELGKM(+15.99)SPGP | SVTLYYFLPQEELYLQ | KDGYPPC(+57.02) | GRSLM(+15.99)PC(+57.02) |
| LVPPQPHTHR | LVPPQHHPTR | NRLQGENEEEDSGALVLSK | NRTNGLL | G(+42.01)SLVTWDDVFVLAP | LALMRYC(+57.02)YDFVT | S(+42.01)ASRLHT | KAGVNFTT | M(+15.99)(+42.01)LLDGFETNQDDGM(+15.99)LQK | KDDTSVVWR |
| KDDTSVVWR | QPNGHVGSPQAGS | KAGLVSGA | HSNLLE | TTRC(+57.02)LTDEDT | VVLQSPP | P(+42.01)TRKSGAL | K(+42.01)AKSDPDPVKGGTTLTVE | QPFQPQQNEQC(+57.02)LQK | KLDVFRALPSE |
| N(+42.01)SGGAATLVNQL | SYPPTVFG | KSLKMPRRV | VELGC(+57.02)GKKPVLSQHHPTR | DTNSFNNQLDQTPRVF | ALGPTVMWQDPFPTVK | YNLGAGSVAGPTAK | AHHPVDK | G(+42.01)KAAADLEGPSHAGPPT | VGAELAM(+15.99)NFVQAGPPT |
| KNPEPSVKLFKLSAH | SPSPVTGN | SAPKVPC(+57.02) | TNSRDNFEY | LVNSKVSEPNPP | VLPAKMAATGQPP | GPPLLTLS | LPPGTLLS | NLSVLSPK | PLGPLTLS |
| NLVSLSPK | PQGNTLSR | LPAFLRQ | LPAAAFVR | TGKPLFVNDAPGA | LAAHQVAY | KNLLVF | TFELPEK | NEGAPYVPTFLPHFNSK | KFNQYGR |
| PSGAVLQELAGTDHAQ | HPDEKQQPQDEAC(+57.02)GLQK | GTLLDDNFKMVGTL | TVVNTVKAAFAHNLVTVEGAVSVLSKP | LNAGAARAGASNSSSEVKFF | TSSTAGPLNEK | TPALAAQVGAH | TEAAKTC(+57.02)LC(+57.02)PS | PLNHFSK | PGSAKDLEGGLLFALKSQ |
| LQSAEPTSYVHGLLLEGGLSVLSPK | DPNDLRVKFSNVPHFGLGSVLSPK | TMSQNEELVA | LLVAC(+57.02)M(+15.99)AATLGPAADYLLQ | LLVVGSMTMQGPAADLYLQ | SHDQKFGKKRLQDSHQK | LHAEAAEPKKLSHNPY | KVALFELTPE | KGLFFELTPEK | GLC(+57.02)FMAQYVE |
| TNPLVQEL | NYDQDATFVAV | S(+42.01)NRVVQQENQC(+57.02)LQ | PETLKHDPAVH | LVPQPALHSPHG | LVPPQETNKRA | ESPPAKNNVVPVVDPT | KNTGVGM(+15.99)GLLAVTP | KAM(+15.99)TALPPVC(+57.02) | ESSTGM(+15.99)QPVVAVS |
| FLKVPPGAEFAALP | KFLVPPQKESQR | FLKVPPGARSTVGQ | KAKTRPQERTKN | KPFHHGAPALLLGV | TNPLVQE | LPQESVQ | TDEHVGLDPN | VGPPM(+15.99)MLARDPVLGPPVLM(+15.99) | PPGVFMAPGDVLVLPGPVPY |
| HAFVVDR | KAYSSHRGRVLE | KLEPVMDGLM(+15.99)KYQ | GNPDLEYEPSDHNGQ | KDLM(+15.99)VVKLPSE | ENLSAKNSVGWQLDQ | PQNVLVFVAA | YVVNM(+15.99)SNDEDRRSLTL | THWASLN | PGSAVQLELAFNFLFWGF |
| APGNAPFEELNK | KETYLLGMDELKYQ | SLENTPC(+57.02) | TFSEYPGKRQT | SQLKFEGPVVTLAPAPPKQSGM(+15.99)LSPK | QKAAQAQQGDM(+15.99)M(+15.99)SLQK | SLALQELLPSVS | S(+42.01)QNGPC(+57.02)FLKYQ | NGAGEQEFLKYQ | GPAAAVHLLLTDPVLAH |
| TLEDM(+15.99)VM(+15.99)TM(+15.99)C(+57.02) | FAGNWEM(+15.99)YMN | S(+42.01)GTC(+57.02)C(+57.02)GYYPPPVV | KSGAYHF | SQGDEFAVALSL | SSLSVREKASQLQ | SPGAQVLEALFPGSAQA | TM(+15.99)TKHKPLK | SGGGKADEFNNY | SQTLPC(+57.02) |
| LNVPQPR | QVVPQPR | GPSAKQYSNKLGK | KMTFFHQTL | SERNKC(+57.02)A | QPFNKC(+57.02)A | EGTTPHMLPEQ | GPSAFLAQ | KYSANPGGGGVVSSQAC(+57.02) | TLGAC(+57.02)AKTFNGPASVT |
| KFNQYGH | TVDDPDLGM(+15.99)PL | KAFTLLLPC(+57.02) | KGGHPDNGEPTM | KAPDLNR | DFVHPC(+57.02) | THM(+15.99)LPC(+57.02) | ALPEELTPGMTF | KLVNNLQEPF | LVQPNVFVAAR |
| S(+42.01)HAFFLVSDKQ | APGTSLY | WTGGPAKKC(+57.02)AVGDDGGGAL | KVNVYELHVT | TTSTFPQ | SNAHKPQ | FPGHVPQ | DPSLLRQPYEMQQNPQ | G(+42.01)SFYNKP | G(+42.01)SFYNPK |
| TNFSDQDLAEK | KYQESQPYRP | PDAEVLNP | TDEHGGTFKFR | DPLGNKNP | SNNPSSKF | SNFTHKF | VVNC(+57.02)NADRVFDADLQEGR | LTVVVP | LTVVVP |
| S(+42.01)VPELYNQPE | TNDSRANFEY | PLSPFDGHSEPDLGVL | C(+57.02)GPM(+15.99)WLVY | EVQFLAPGSAGA | PSGAVGALEALFNFGWLFF | GVATTSAAW | PC(+57.02)C(+57.02)DLMVSDSHDEMC(+57.02)MVMC(+57.02) | KTYDFEELNK | KYTGGGNPNKFPQC(+57.02)A |
| S(+42.01)AM(+15.99)QRSYLLALVTP | PC(+57.02)RVQC(+57.02) | TVM(+15.99)KLVAH | KLFVTH | PVALHEQPPLVMPNFPTR | A(+42.01)AGALVQELAFPGSAQAVEK | KNALLELLNESAEPAGPSP | S(+42.01)DKDFRGVADEKKETNR | ESSYFQADVPQ | KGFGLGM |
| DTGAELDHEC(+57.02)SEGMADPVDGWHYQ | TFSLNKGVPH | KEMPFVTVVLA | WGQKKNLQGENEEEDSGALVTVK | LFGGHNSPLEL | TVVAPLM(+15.99)NTE | C(+57.02)NANQGSMMVVVWLQEGRVL | SDNHHYTSF | A(+42.01)PVRVELPKELVGDPDEEAGGLKEHT | VVDRQLV |
| EGAC(+57.02)HAATNGKRGTVK | KSLKQRLELS | ALPEEVLQNPVLALPMAH | HAFSVDKQLAKN | QGGSLNLALPEE | ASYDTKFEELNK | GVSEDFEVTRETERNLR | LNRLLTL | A(+42.01)KSYDGESSFGRC(+57.02)SSNGMNC(+57.02)TSPYVANHF | EPQDFYPDKETEGGR |
| QPEDFTKC(+57.02)AATTEGGR | SSAALGNNLGC(+57.02)DLSSDD | TVENRVPQTTY | LLVPDAKC(+57.02)VQ | KNLPQEHKVT | GPSALAFQKDNNLLK | KLLHEGGLARSP | S(+42.01)HAFLFVSDK | KSLQEAR | S(+42.01)HAFLFETAKQLAK |
| TEPASPANA | KPHSLYR | VPSVVTLP | SPVVTLVP | SSPDLYNGNYQ | GPSAVGALELFAPGSSTTGPL | S(+42.01)VTSM(+15.99)TYNNLDQTPRVF | VPNSGLGPELAGGLLSSH | T(+42.01)TVPPHSVQVH | ATSMMVAHHC(+57.02)PQPPVAH |
| KAC(+57.02)EAPGSWASYAF | KKNRVPC(+57.02)C(+57.02)C(+57.02)PQ | GHGLANTSSPDLYNPQ | KENVGAC(+57.02)C(+57.02)KDSHLQK | TC(+57.02)C(+57.02)SLLP | QHLVTFNLKSQ | AMGLYHVFC(+57.02)AGVPLLSDD | KLDVRFALPSE | TVTTPAAAVEPVGMKAEGGL | KC(+57.02)GSTPLFNF |
| A(+42.01)ALLQGWPSP | VAGVNPC(+57.02)VLTKP | SSSRKTLSTDDKPFNLR | NQLDGANPVRF | EGTYNTGDM(+15.99)SLAVVT | KFLVKGHETEL | PSEMVNGPQVNRTPVTNY | KDTGLVTYYYGGLKSQ | KAAYDVHKANPFYLR | SVGAGVSLAK |
| ESVGAPM(+15.99)KVDAH | KELGSNSPVKKN | KFLVPEQPTNK | KFLVPPQEKNT | KGLFM(+15.99)YDVLT | TGFVKHT | A(+42.01)AEVGFKKLRNVLSN | EGGAGQAQC(+57.02)LQK | HNAVTSY | PGSAPDSLGRAATTYAF |
| PQHHTNLPVGGLKFVKF | KNVPLKSVLVVQ | QPKSYVPQNF | HAAAALT | EYVVVQGVEKTKNAYLDPAQLQSK | TVFFHPGPM(+15.99)SK | QGSC(+57.02)NEC(+57.02)PVR | ANSC(+57.02)NEPC(+57.02)VR | L(+42.01)SVEYDVLSENQLDLLHMALVAHE | PALDFPAQAPAVT |
| K(+42.01)GAKGFAFDAQEPTFEKQPAAN | ALDGALATSAHSQVALKLPR | RDAAPTQQLAN | RSKTLSSEDEPHAKPPSR | TNSGPLVSE | YVVNPDNNENLR | FPDTGSSAKGKVSDKPFN | N(+42.01)SLKDFETVPKN | DPLSSYNPQQEAPSP | SSSNGGQLDQTRKSQ |
| PTGVFQVVHSRAW | SALPMDTL | TLFEEP | PFKYYLGNAMPFPKLF | AHAALATDGLVSL | TPAKTLEPE | DVRFALKLPSEVLAH | SPDVPPSEMVDGVTLFAGW | TVGNGEDPVAVSL | PVVAFGTDC(+57.02)GM(+15.99)RDSP |
| SSDDNMQVSF | TGAAEGGYM(+15.99)PLAK | N(+42.01)SSC(+57.02)PGEEDVLQTVK | FNAVLTEYLN | PVLVAVS | PSSKAASPDLHKVRFPQ | GLAAPLGGLNPC(+57.02)EHLMEK | FHDDGLPKSTM(+15.99)VPSL | TQAYPPC(+57.02) | FGPPQGVPHAALL |
| S(+42.01)PELPVKSHT | K(+42.01)GVPC(+57.02)RFPNMRGVVQLK | HLENPLE | LVTGGDSLGLRN | A(+42.01)KGSDLAR | SHDPLYNKSLGK | NPLASNDTHGKF | SVLTVVP | APGGDVDAQPQQKEEGSKGR | STDAPPVL |
| EPVRSHPPVPE | KFYASVWKGPFKFL | ALGLNAENPVFYVHT | K(+42.01)GVPLPPRKDYTLQTLC(+57.02) | YNPKARG | KQNAAHA | EC(+57.02)YDLC(+57.02)RPC(+57.02) | EPDGHSLLPVNP | TGNVEGLVTVK | ARYPC(+57.02) |
| EPDLNNQPQ | TLFQPLT | TLFQPLT | PQFFSAGPQAGNEQC(+57.02)LQK | TC(+57.02)FFLK | STDAVTPTL | TGLGDKPM(+15.99)ASTL | KLSAM(+15.99)GHSYGLKSL | TNTFPC(+57.02) | KDSVMC(+57.02) |
| ARQVTNNAAKDSF | S(+42.01)HAAPTPQ | SFTHKYTESGL | KVQARNTGLSNSF | KQLMKC(+57.02)QDQPQQEPQ | TVAVDEALQNKR | NSLTLFLRAGPA | S(+42.01)LAATTTLNAAADTTPGLLK | ELALPLSSC(+57.02)M(+15.99)HLKAAVE | KHKGSSQLLLAAT |
| NNNKLFP | KFPFYLTQSLGFPKF | ELTM(+15.99)NHLLAVTP | V(+42.01)VLTM(+15.99)ADDVTPAGGGYSEGR | SAPKLLT | KELNLL | KFPTSSQPLYQ | SPRVTGTY | DTSNEHMQLDQTMLLAA | RVDNLTY |
| KLLGPEKGRNPPQ | KDDVKLLSPC(+57.02)G | FSPQHEDLVPFSFLVDGNKRALSH | EPEVTPTLKALQGA | EQFAAPMKYQ | NNPFKFL | S(+42.01)FLLPPSKKSE | ESRYFVDAQPK | RVDMPTY | A(+42.01)QVEMSYEM(+15.99)C(+57.02)KSSEDDSSSSM(+15.99)S |
| WTPNNKNM(+15.99)EGM(+15.99)PYLAK | G(+42.01)KDETLM(+15.99)NMR | EC(+57.02)FDLC(+57.02)RPC(+57.02) | S(+42.01)LM(+15.99)NLQSLAVP | ALGSKEAW | TAHKASNTNPQ | KALTSVP | TLFKNQ | LVNESHTQGHT | KLNQFKALPSEVLSGG |
| PVVGGLGGFPPC(+57.02)EHLMEK | KFLVGTQAM(+15.99)FPSAKADPAG | F(+42.01)LVEHRPKAFREEEPLEVQRY | EVTSNFDNQLDQTKRSQ | VPVLVNGLHAASLWVNC(+57.02)EQQYSKS | SPGAPLYGTTRLKLVNLLYYTPNP | KC(+57.02)FLVPQ | KAGSWHQ | M(+15.99)TEEQDENPYLFE | GPSAVAGLTTKFPGSAAG |
| PLYSNK | TLLGC(+57.02)DDHM | TGLPM(+15.99)NPHLVP | S(+42.01)PHSNSPM | NEKGGTPNPVEYEEELLSGFAPEF | ADALDLDVPPSGWEKNLPN | TLWHNTWKF | WAMTDEENSRGYG | KRTLSSEDKPFN | KLVGTM(+15.99)VN |
| TFGSLFFEPDKNPTLAAGLSGPD | N(+42.01)LVSGTPM(+15.99)GALLLPHFNKS | PVEENGYPETM | TPEGNM(+15.99)GYRLQ | TLGGTMGGNLGQC(+57.02) | AHGLVPTLP | SVLVELSKSNGKKPV | HAVAVTPLP | LDDDKRKKGPL | PSGAVQLELAFPGEQDLFF |
| KGARDLLTYSQPLGK | ADSLDLDGYTMDELYVKH | VPGGKNLVWSM(+15.99)PPQE | TTARALVQ | SLESNFNDQLDQTVRPF | LVGAHDGALATGDLVSL | PGGTVQELAFGLP | KNFSHC(+57.02)GPAPL | EGGADKLVTM(+15.99)PVVTVK | VVNPDDNEVQR |
| PQLKTSAP | TYFRGLEGQPTKNAPEVTGWLQSKQA | TGVNGHLTKARMLHQTY | ENENPYLFE | VTGVEEC(+57.02)EEGMKRRFGTTQQKEV | LNNTMFNEYVSFK | TEKLLLNYT | KAEFTLTTY | KADMPLLGVC(+57.02)GM | TVGAYKYEFLL |
| N(+42.01)SSSLLDTSPPTVPGHVHFR | KGLLLFETPEK | LDDDLGLRRVL | TLEFLPE | KGPPAGESVLTKH | LMHRSFPNGGLKYAAAAY | STGPRYR | QGVAEGPVVC(+57.02)GFG | EHNVGEYVVKPE | A(+42.01)SNRPGAHTPAQ |
| V(+42.01)HLSANFSLLF | AHELYVLNHGSDKLGAH | TGFLPHT | SLHSGHVKC(+57.02)ADLFNFANA | TVALLEFGKDVS | NNNFPAYLVPFTGKLFGM | KDAC(+57.02)RVPEHAKPPKN | STGSPQNT | KKTFTH | THQNFLK |
| RYLPC(+57.02) | TLEAHKPT | KPFAKLSTALTL | TQNQSKVLKE | TAPHYGAEFLAL | LKVNTDAQ | NNEM(+15.99)KHGLVS | GAPANM(+15.99)KLVQPA | GPDNLAVGFNVK | QPFYFVDAQPQQKSVPANKFQG |
| TPNKWELTPE | WGREWRC(+57.02)GWFRLE | FEMKGKPKTTPP | LLDDKQSTDMLDQTPR | TVVLC(+57.02)DEEEEGLKKLHA | K(+42.01)GAGDGTTM(+15.99)C(+57.02)AKLAGVTVK | KSATHLTKDNAM(+15.99)YLT | KSVTGWF | TPSAC(+57.02)TMK | FSVYK |
| FLAEKYLPC(+57.02) | SFKPTYN | TGGHNAPAGSGAKNTT | KSRPTLSDGYLGR | S(+42.01)EVPAGLALHTFNLKSQ | KC(+57.02)GPNMFEYV | S(+42.01)LAPWLQ | KLLNQ | KNLMFTVKKLQ | TVEGALVSL |
| SVLVM(+15.99)LC(+57.02)PLM(+15.99)AAHT | TYYVVNDPAEPQPFA | KNVGAETDEFNNYKL | ALMHHFG | RMAPSLLGVGC(+57.02)MPDPDASYPTGLES | TVPGEKKVDLS | TGPKAATP | E(+42.01)GGEYLTDQLDQNPRVF | LVPHPPKEVVPP | TFTTAHYPQVK |
| KPWGLT | TLLTTTTSLMQ | KC(+57.02)PDLT | ESVHNLRWKFDFWYSRSLTKPQ | KNKKKLLLDM(+15.99)DTH | QLMYFYVKKFGRFE | TGHKLPGQ | KLYQQQ | KAKPLPC(+57.02) | KSRLTPSNYGGR |
| QPKTSENHTKPQ | TREVTERNLR | HGGLATDDDYVRPYSKGLESAENK | NSGC(+57.02)GWAF | LGTVGPVR | NSPHDEVPFVAK | KFSNDGWEYVYAKSQ | LLAWAC(+57.02)SPLPT | LNALSGSETKGAH | VSGGPTPPTTTGPAAC(+57.02)VGVTSK |
| EVDSFVM(+15.99)DYKRDDLDLKPFFGH | QPNAFVLVAAK | Q(+42.01)RFTNEPRQEWGGGSLL | NQLASHF | KSEQLRQ | QVVLDLC(+57.02)RPC(+57.02) | GMVLYTKKMTGHHLYLQ | AGEGGPNVYDMVVFKTH | EGGGKDEGALVTVK | VVLTVPGP |
| VVLTVPGP | TTMSVF | QVVMTSVPGASAPQ | NDELNRMLTL | FREETAL | KDSLQGM(+15.99)PQEPFNLR | GVHLLFQ | TVDLLNVSPK | DPNNPVNMNLRLLTL | NLRLLTLA |
| S(+42.01)GGDFMDEQGALVTVK | KDAGFAVSFLLEHAKN | PQRTTAVK | WVVLFGKLPSEVSLNSY | KTEVGQKNTPPRKSYNR | KFHHPT | KVHAQVVRPTY | NFSLGGTALGNDK | KRTLSSEDEGVSTVLASGV | DPNDGSVVVNLFPRTL |
| KDEPALSTGWFNLER | KNSWSLAALPVC(+57.02)RSLQ | TNPKSLL | KASQPEDHGGVYSFK | VGAGQYQPGKRLENLR | SRYPC(+57.02)P | KPRTLMQ | ESGPPRTSLLLSE | PEEKSGVALFHAYQLK | TVVNNDDDRSYR |
| SKSVTLEN | TVEADAGSGC(+57.02)HDDTAGPRVF | SSVVNTTGSGKESTLGVQSR | S(+42.01)M(+15.99)SYPYWLA | KGWDSNGRPNNSFK | TRVLVVPQ | LAGVNFGPKHKT | EQGHRAAFAHVTPVE | NPSLTMC(+57.02)KVPL | A(+42.01)SAGALPVSKGFEKEANK |
| TVDEAALGKNR | LSKSSVPR | PGSAAM(+15.99)EKLLVPGGT | TVNRLDVTR | T(+42.01)GKGGETPVTGGTARDAEAR | KVMRAL | WTNKPNNESRC(+57.02)ARALNK | VVTM(+15.99)AKY | TAPGHYAEFLQ | KAPPWKDPMFP |
| S(+42.01)PPPHYGRC(+57.02)AGVTVSK | P(+42.01)AALKPDSTVGAP | RDDKYTLGGNR | QAPKGM(+15.99)VNAHT | SPGALVQELAEAHGSAQ | EAAKNLLVPLVC(+57.02)M(+15.99)EAAK | PGLENAALQ | KDAGKLC(+57.02)GYWVTP | TNYPSVNSRLQ | DSSDC(+57.02)M(+15.99)VGALMGDTKLSDD |
| EPVTPPKVVPAM | S(+42.01)PEVLALQ | EPEVALLQ | YGTKVRN | KNELDKAGFKVLTH | TQNHTPFNVAH | SPETPVSLYGGP | LRSHDPLYSNK | GFGGLHAAGL | S(+42.01)QGGPMKPDQTLEPQ |
| KGDNLVHSLRALDYT | EPSSSFTTAAVETESLNR | S(+42.01)SSYFVDDVMT | QQPAGHTVLYQWPSTFEEPQQPQQLMP | THHKESH | PEEVRLAMHTT | PLASLEPAPLLLVVPAPV | QDGMGVGGQLLLPHFNKS | TVAPTHFSQLK | ALGATM(+15.99)EEDMESLQTPKGK |
| TDVLKYE | LSAFLPVNKFC(+57.02)AEVTL | KGGGVFNATSGDQ | KNAYC(+57.02)AHTLLVQ | TDEMAKKKAK | FFEPQGAEPEGLTVDGLLPDAVYVNGH | LNAKLPDN | EDFKRGALLTTRPT | LSSAAGRGATKEVGQDLAGSK | LSVLDGMTDLLNSESFMELVPV |
| EDHVGTELSTAPLDQ | SNFTQL | TVVLSKP | TTGVLLAKHMT | QAYGGTMKYGSL | KLQSPDDEKRQ | VVFDEDM(+15.99)VF | QPPTVREPGAVVTYSELVPLEVPAAHA | QPNHNTSVLAK | TVQREDGWFN |
| LSNPPKGLPQEAEGQPPSHS | NALSHGGTPVKGYPARKSQ | TENHHKC(+57.02)PS | SYPTVL | LVSVELKSRSVGKGVLSVH | APLVLNKGLTGM | LPADKAATL | LTEVGPDDEKSWLQLR | AGGLLSNGALPEEVLGAH | TSYENTAQLQ |
| VPGGASLPPLSAHS | M(+15.99)VM(+15.99)GNAVRLSMLVAQASVE | TLMGVSLKYLPSE | SPGAVAGELAKLFPGSAQ | LVESVLSKEQLRAL | HGEAFLLPSLK | TNDDKM(+15.99)YVSF | TGFGLARPVAQTAH | EESSKEEFVLT | SLLC(+57.02)SHC(+57.02)WRNLQSVTVK |
| YFVSEPK | QPPALKPNNNPPEQ | ENANC(+57.02)C(+57.02)DGAKLVTVK | QPPM(+15.99)GHVVLVPPEQ | SPGGEQALFVE | FHFGVSTMGLDADVLALH | A(+42.01)ANAGVNAGLFFAPHFNKS | TVAAFRVGM(+15.99)M(+15.99)PT | QM(+15.99)RC(+57.02)LDFC(+57.02)Q | PAAAGAVFPPASKDKLEC(+57.02)KNSYAGAPA |
| APGSSAHLLGVLVTDAYF | HAVALPTVALNPGASHF | NNNPFSF | QSSLTEKGSQNMAVGSPK | QPFMMELKVNL | TPAANAQVTVLLGAH | PDLKTSDDNAGS | TGGHNEPLKEGC(+57.02)AVGTVSK | KDVFRALLYPSE | TGFAVPPGATAKGHT |
| TVGGVSWSAPLVQ | VFDAQPQ | QLSFPNADPKLENPT | KAGGTKEVGVYVVHKSGQ | TDLFYNP | KGGAGAYLLDQGL | GLDETLC(+57.02)TMLR | KPVPSM(+15.99)SDQLFVLAP | G(+42.01)VLSTSKNDHPSYHSLR | TLSSEDKPFNLR |
| KM(+15.99)MVMTVHKQ | STHLTQ | QFAGPWTVPGPS | QNQLDFELALQN | KGLLEFLTPEK | KGASSSYLP | KGPSGGPDYYAAPGSGKLQ | TNYC(+57.02)EVKPSY | APGHTVPVASGSVFRALPQ | EQLVTVVE |
| K(+42.01)GVSSLDNVAEK | LPGVGSSGF | KSLKNNVA | TSLFPGC(+57.02)PSTY | SAFANGGEK | S(+42.01)GPHHKLVLTTVMLHAP | QPLPAPFSNKVF | GPASNQLL | ESAGELDQGEPDC(+57.02)VTYYDDKAND | TGFPREPSLAA |
| HAVSFD | ALLSLAPSGNYDPHNVVPGA | NLALSM(+15.99)PKLPPS | FTNAHM(+15.99)RDLK | KDPLPSQ | TPRSKFSDGYGR | KHSNLKN | KAELLADENGYLSY | W(+42.01)LVPLLVVLNLPTVNDGSTGLHWAN | KFC(+57.02)NTTM(+15.99)GAKQ |
| NSGPVLQVLGAGSLGAH | VSGGYNWGVVFDDALQEGR | THASLTQT | LLAKNAHSSSPT | PFFKGMKALTLNPDPRT | FGTGVHAGEEAMYPVNVQ | S(+42.01)SEGNALTEGVQKKKME | KDDYLKEGFKGGA | VTGEFLNAGDSLDGATALPAF | SVGALRAK |
| LPGPTLLT | LTVVQSEGPGLDGKLGL | AAAPESQQGSPRVK | TLLHKVT | SLLTPLQ | PAGNFVNEAVVLYTPLLK | P(+42.01)SEDFYQKNEQPE | AGPNFAVLLVVAA | QPLRDFDNKLL | EC(+57.02)FDLC(+57.02)RPC(+57.02) |
| N(+42.01)SRVVAGGAADGC(+57.02)QLQKLN | KDATAKDGNSYEPEQ | H(+42.01)LVGFK | DPDAGSSQLFPMTVHAAHAA | S(+42.01)PGAPVEELKTVTPDAMDGWFR | HGGLATDDDYKALM(+15.99)VGEKGTSQ | TDDNRQLFLE | VGPPLVPY | GPPVLVLM(+15.99) | GAVAWTDKSGGM(+15.99)AKPMLLE |
| GSVQRKYSTSPDLSDGHQ | VWGTLEDKDEHGKVFM(+15.99)T | KEVFVR | TLLC(+57.02)DSPPQNT | S(+42.01)SPVALGC(+57.02)LFNSLGQSQVRQL | PEKNGKTGSLPGGALYTLNGS | VYMGNAVRWLLVANGQSC(+57.02) | QPAAHLVLATAGS | ENQFMMTFE | TM(+15.99)EWVR |
| S(+42.01)PVEDPHGVHANQC(+57.02)VATH | ESRPTGPSAKLE | KAVLDNFGVVAVEGL | DHAASGLVEVEAPLFVTHLKNC(+57.02)KG | SAKVC(+57.02)NLA | GAPAGPAHNYLETSSC(+57.02)GLGAR | KSSTAFKVKLL | LTDKTSRPKER | FLVRPQAAAE | KLPDDEKDFNQLEL |
| QDENPYLFSGPQM(+15.99)KLDM(+15.99)GSVEMLNQ | NNEDTFAVAVSL | KELKLPHGGDSK | FDTFRNS | LVGGHMNKTPQ | SNKSTPC(+57.02) | HAPDLHKVLSTLPFHPA | DYLAHWEMDL | S(+42.01)GGPVDAAGAEAASKDLSPGPAA | TEDKKM(+15.99)M(+15.99)Q |
| TVKLMPC(+57.02) | KNPVLLRVPAEF | KM(+15.99)KGHPVS | VEGPKMSEKPERYVDDLNPGFGHL | PQSKQYSNKL | LVLSC(+57.02)RDC(+57.02)HKGLEPSGY | KAC(+57.02)DENFVK | SELSNTAC(+57.02)GDLDQNPRVF | TPDPEDKSGPR | APGSPGLAGSGAARVPPRT |
| ENPKAHHSNKLE | SGGGEQQGQHEEEGVK | KTNDHKGC(+57.02)PVAG | G(+42.01)VGVC(+57.02)FGLALVPTGVA | HQLLDTLVSGAVLDPVTMK | S(+42.01)SKMM(+15.99)PC(+57.02)C(+57.02)DLC(+57.02)AFSDQ | KPYHPTVAKLF | NFALYTTKYESTSP | KSGALVTVNGSPQ | DFGLQPGWFN |
| P(+42.01)PGVPTDQDPSGNDFP | G(+42.01)KARKSGSDPKDMVHAGPVLDPAP | TDQLALC(+57.02)TMKLHERARAKPD | VGGLLVMKFPHS | EVLLSTDFNQ | TVGAGLFDKNSALS | LHEASYLSLLGQDLQSK | LALC(+57.02)HPSL | KFNFPQ | DARHYLEGKTVGTVGAGR |
| ELKLFDWPTDNGGPT | ETDWSAADGNKSPLQ | TVLM(+15.99)ESPMVQ | VVNC(+57.02)DDARVFDGELQEGR | TPGAAAKSAVPTGF | ESRYFVDGAAPKKKEE | S(+42.01)GNKEGLMDGWFRLE | LNAC(+57.02)DSM(+15.99)YVSFKTN | ANLHRNGPAAERGHFLEF | TVSEATLQ |
| AFNYPSEMVDGVVHADGVP | TTDPNWRHGS | ELKGPDHVTSPSE | ALPEEVEYSLF | LSGATTVPKVEKKEPNVSHRTGVV | KFSVTDNQPTKGN | LSGGSGTLMALFPGASAAGVEK | PGAEM(+15.99)PGLAVTSGPL | KC(+57.02)FFYPLLE | KPGNHGH |
| LVTMVESTGQQQGPPLSPY | TVPGGSAKGGQPLDRVELF | TNVGSSNFEYV | AAPETTAGEERVVPGGQAPA | LLKMETT | PAQGLFGM | QVLPSSTGAAKAVGK | RVVLQ | TVFVPHYVVNLR | ELAM(+15.99)FPLSGAPQ |
| SNLNFF | KQAAQAQQDC(+57.02)DGSYQK | TNTPVLQ | KADNTPHVVPL | GPVTC(+57.02)LDPGFPRTWS | TVNRQNQHLVS | ASSPYQPQAPERAAAR | PDDPQTM(+15.99)KDSR | VLSEQLVELSKAKLTEAP | P(+42.01)FHKNDASR |
| SYNLAEQKAQ | TVPQVGYKDFGP | TRPQNSM(+15.99)TGSN | TFRAVPNSLSL | KAENLPDTTTH | SLHMTLLVM(+15.99)LA | DSPLKDPVHGLDPGKVSDD | SQKGSNPL | QDRSVAWLHS | QPTTYLYLVWPVELSKGHYLKL |
| E(+42.01)KSVQVGPAAMYTKKLRGMDLQKS | HFVLL | V(+42.01)SEM(+15.99)NSQVTLD | HLGLMNLEPSNVLALHVAQ | FLQGFSQPKQQ | SDALHKWANS | VTRYPTAKKKGGF | GPVKLEAATLLFLLPM(+15.99)VQ | C(+57.02)NKGNAVKPVGFGPPSLTGF | AARDKNEGLVE |
| TPKM(+15.99)NAA | TDPVSYS | QGVLNTVSKQDLQRTAH | KAANHKTFNLR | LDTSGGFNDQLGGETPRVF | TKGGNTSFQTL | YSLQGF | EGGAKVQNEGEDKGALVTVK | QEAGNQEFLKYQ | G(+42.01)FVKDPPVVRRGDSVAGKN |
| NSLNFL | GPTHKLFC(+57.02)MVVGGRFAK | GVALSRGGPYNRN | KM(+15.99)STAKKKKVRPPC(+57.02) | TFAHLNRMF | NGPDLC(+57.02)HQDTM | SHNLKNTSFHSE | QSYLQGF | PVVLNRGFFQF | ENQLDLLVSTMSLDQ |
| EFSTYAKVKVTNNVE | SSSKNM(+15.99)TL | TDDDYKVRMT | TEC(+57.02)QPLLRLT | TVANLVVKAPGP | P(+42.01)LLYMP | GVATWNPNNKDSGKDT | TDSSC(+57.02)QMSEL | F(+42.01)FLSVSTGH | KLNPSM(+15.99)AQFSE |
| LVELSKK | TVAMASHALSL | AYGQPQNFEVYSFKTN | QELPETKVFA | TAGAKTTLSM(+15.99)EP | SSNADKNTYLN | LDAGHRGVVMQLN | KLLQSVK | TLGGGNKFPPVLTAAKKDD | VNLLNPPVAPLVTSMQPLY |
| TDVNGHC(+57.02)GGSW | S(+42.01)GGKKNDGWFNLER | KEVGLQQEKPW | KGFNFF | TFRLYQ | TVGVQKKKEVKPQPQMPVSTPL | K(+42.01)WMTPQDLFVLWWLVFLDETQRAPL | PVLVGSSWVC(+57.02)PFKVN | PVANSTVAG | TVGNQPFVTPC(+57.02)AKVVMKPFNLR |
| PAGNQVSFLAPVT | PSEVLAHSSQPAALGGGT | SFLVPPQESRQR | PDLEYGNYAGAMGATH | WSLSLR | LFEDVFT | ANGPELGGQLDELVLVGSSAR | KASPVLFGEEEEAPAGLME | G(+42.01)NTKVLLA | ELHM(+15.99)SYGKSLQ |
| KSC(+57.02)GNDNPHGLESC(+57.02)R | KYKKPVESTSP | PVVADDELRVTAY | APGNFNVLPEFRVVPQEKNTR | TLKPQC(+57.02)P | KSPRVPC(+57.02) | ELFDKQ | K(+42.01)NTTPSSSTTVHQEETH | KVLGAQEASFDGN | APGVGKPHENYPGAVVAEH |
| FDGKNYVLSTL | KMSPKYQAGPF | EGSGKPSLEGAGTVGTVQGR | DSATLF | NTM(+15.99)M(+15.99)SSAF | WATSPYGAGQQGEAARVGAE | TGKNNVVLVE | KGGASLELPVSHT | QVVFGVHK | KLADLDPRELN |
| GVEENLSDEPPD | TM(+15.99)ASVPKKKKFAM(+15.99)T | QLEAAHAL | QSQQLQDSHQK | GVFLATNE | PHHADADNHRLFFNKAY | G(+42.01)YVMKGLTPFS | TVGEAHKKFVVTF | SHGGLSSEEFDLKPDGM | KNQQGRPPTLQ |
| TAADGNKSYHP | DQWAAKMSVLPRQPPAEPFNLR | GHGLAESDDYPYRAK | TLFKFGNSGR | A(+42.01)KVQGYQALAAM(+15.99)TDDALQEVVAR | KFPKKLTM(+15.99)EP | TALVVKNQ | S(+42.01)VNKSGSPVPPVVDPEPQ | ALDC(+57.02)C(+57.02)HSKP | DPNVVNNENLR |
| SGNLLQYAPE | EGLTHEPVHAAKLGRGY | KVQASPLY | TAAAKLYNPC(+57.02) | NSQHWPELKC(+57.02)AG | GSRTNEPVTAEHSNK | TNKYVLLFDT | TDGM(+15.99)NSVLYL | LTGLM(+15.99)VSSSYKPGSML | TDSNRFH |
| EGRDFLLAVPT | TAM(+15.99)QYAVLKAA | KLLHELGGARSP | TDPSYKMVM | RAFYPQ | KNDALDAMDWGFR | PQNFVVVA | PQNFVVVA | K(+42.01)AKFLSSPDLYNYGNQ | K(+42.01)DETLKDNM(+15.99)S |
| TVGALPQSQ | G(+42.01)KRMLTL | DTDDHHSQWKLYSNK | SEVTNFN | NPFVVKSC(+57.02)ALTVPL | GC(+57.02)FDGWR | KDLVFLENNQVTPAG | KAANGAVPFGTLENLR | TVLYALNAVAR | EVERQPLFGPL |
| THLDEPGFGHKTL | LTEVGPDDDEKSWLQLR | PGSAFLAQDVER | KKTC(+57.02)EQPNLL | K(+42.01)VYYDLPGDPPQASVTNR | TSPDEVRPLSGT | TLM(+15.99)SGPVAQ | KAYTFLNMPT | TVGLLVK | TVEFGGHVRVLQ |
| K(+42.01)GVTFFPEVSSSKNT | NALNLEPDHQGAK | VPTVDVYKAHTLR | ALRPVLGGS | GAPAADGNYSNLR | QGGYLVKEANLA | HVVTSQYFLK | SLSC(+57.02)PC(+57.02) | RSPTFKLLYPSEVLAH | S(+42.01)PVNC(+57.02)GEPLEYDPVTH |
| SSSPYDDKSDNHPLGVTN | APLVLLYMTPP | TLLPTAQ | PPAANEPLVQAH | QDDVKNGDAVLL | SFGLDDEMTR | QMNFLLL | KFALVE | KC(+57.02)FGVHFLE | S(+42.01)DTLLNEEVDEMNRK |
| KAVGKKTGTAGSPGLTQKVQ | TQHAKKDNNFP | SEM(+15.99)TYKLAV | EPHM(+15.99)VSEGPLK | TLLGFDLKFGM | TADRC(+57.02)HS | QPFKVKGGHKEQ | TLEFM(+15.99)LRC(+57.02)Q | KDPLDLLSELNATYT | KFATSSAHVPLAN |
| PGLEHTSGN | LTGFAVRL | LPVNKNAGM(+15.99)LTPL | S(+42.01)NGASHGVAADMNMVE | LPGPATKADWHLE | S(+42.01)QPFFGQQKYALKSDFTTLVELSKE | KNPPSFPDYTLQ | KDPDHTAQ | VVLHM(+15.99)PMNNEPSP | SRYPC(+57.02)P |
| S(+42.01)NGDC(+57.02)YM(+15.99)LKYQ | KDKGDLALDSGVVVTY | AFNYPSEMVNREFAQLT | KAADARALLYKATNSH | TNPLESGPV | S(+42.01)AGDPSPPAHKAQ | QGAEKLAAVQTF | VLLDNTKVVVA | E(+42.01)SVEWWKQQRWTDE | SKVKSPC(+57.02) |
| KAANDNARDAVWATGPTGAK | KDSQWNFEYVSFK | TVGLVSPK | EHLNKNSNLEAH | TPKTEPLDLTF | TVVWKPVEMAP | HLYQPQ | G(+42.01)LDETLC(+57.02)TMR | G(+42.01)LDETPDSSFK | VSGKDTGGLEKADQNRVPF |
| KENTPSTAAVSASK | DHVSGNNAPASEGGPPEVNH | KDADTNNFEYV | N(+42.01)PLQTSLVKPQ | E(+42.01)SGAHM(+15.99)NSNQEVGLVELSKERAKSPV | QLATTM(+15.99)TALKVMHEAH | KDEVQLTDTLR | KDDELHAKLYYF | TGFAAGLKE | TKSDM(+15.99)DLVLH |
| AGPGLLHEKYLP | TGGPVLSKQ | QPNFGLVA | TAKSSLAGAA | TPGPDQLLQNL | SPGVTKGLN | LKGLMNRLFEKLKNALEDYSNPV | VQGTNYSQ | TGFLGDSNA | TAGLKSGAANAVAV |
| TVSSDPVF | TGFVLQGK | WSLVVEVGMLDDSVSVHLENVKER | KGPFEFFLVPNLHPE | TGFRC(+57.02)PQ | TVGPKYESSPQ | SVGLQLFGVFGFGYAKNL | SVFGFPQ | STSNGPKYFPAAAHLK | GPVTC(+57.02)VEGTAGVPTC(+57.02)L |
| TNQLLHSTAPE | E(+42.01)SSNRFLALVKLEQNSE | KDFENLVPEL | QLLDFNTDLLEQVFF | PEEVVAVAGKAVYVNETL | HAFKVKR | DTDDEDDGHVNLTRLPF | SYPTLVE | SEPYVS | TGPAM(+15.99)AVLH |
| TQVFAQQ | QSPAHLLTVGAPAK | KDSSFPT | TRDVWKETPAAH | LLGVDLQTVR | TNLRAMGS | DLFAKKHEVT | TFLLVLYPH | GLTVDEALQNKRLFL | STPAAHQKYLKPDTNTVP |
| AGENSVLLA | KNLWT | TFKKKLVWK | QTDM(+15.99)SSDSAVPVVSL | SPVVVLTPGPLVT | KMVGEPLHFGGLQ | SDNFVHLSPQ | TSLVNRPFVE | TSLVTFGWQAST | PVVRGPPKNEDPPPKAH |
| DHALTPWLKAY | K(+42.01)DTRTVGSGM(+15.99)LDQARPAAF | TNEHC(+57.02)SQTKP | SPTM(+15.99)KVN | TPRSKLSNYGLGR | A(+42.01)KDTTNQDVFV | KSVGKLGYQSATEVPT | EPEVALNLLQ | SFGVVQYPLPF | SAFHKKVMDAT |
| KAAAYESPQLKNP | TNEC(+57.02)PVR | QDQLAANSYGF | AFNYPSEMVDGVPPTVSAH | R(+42.01)NQARNLASLMSFLVPVGEKLM(+15.99)AH | S(+42.01)VTATKGANTATDQMHVFR | KNGGLSEQDLFV | ESRPVLNHAAPPKLSHYAG | KFGSYLT | AAPQSVLELQVTEEVQRY |
| KVANLQEP | SHNLKNTS | HAFKKPHGYV | S(+42.01)SSSSKVTNNDQ | TTELHKHPH | GVSSKSVTAHLLGL | TDGLAFLVQMS | GNSVVRLKM(+15.99)GMPHFDSK | S(+42.01)TVQPM(+15.99)DGDPSWLQR | TPM(+15.99)VGSF |
| QATMFNKQST | S(+42.01)SYTHAKKMSGA | QAEDHLM(+15.99)KGF | SPGALNSKSSSGVP | SPVVAMTKPVVQ | TAEPLRDMGPL | TVLANLPE | KGGGPPDLPYKNSLGK | SAPQKLGQ | DTSNFNQNLDGATATPVFF |
| SDGPRNFLAL | S(+42.01)VGTNDAVYGYDQTPR | G(+42.01)KALHELQEAF | GYLWFRLWNPPQPPVQ | KDPMEPPEAVAP | KNEDYGF | KSRLTPSN | TMAAPWPL | A(+42.01)ARDLTPQ | AAEYVANK |
| A(+42.01)SSDVEYRFC(+57.02)VGGLA | KVM(+15.99)MNPALYE | DPLAC(+57.02)M(+15.99)GC(+57.02)QDENGVK | TVKTPPLN | PQNM(+15.99)VVC(+57.02)C(+57.02)C(+57.02)PVYLSGF | V(+42.01)GGFLAPAVDHSTVP | KEHLNFSAAH | TTSKGVLL | LGVSAC(+57.02)LLVLLVDTPGAK | SSLSHSVGGC(+57.02)PSYHRTV |
| LC(+57.02)GKPVTLFYASPQ | LAFALAGNSEDGF | PLVKEDMHVPLF | N(+42.01)TRSSFQPSHM(+15.99)QC(+57.02)LQK | PPSMQVAM(+15.99)HVTL | LSDM(+15.99)SDNNSNLQSDGALR | AFGPDLMV | G(+42.01)KLEVNQDDHT | SFQELLDAGQRTARTPSH | KELYPC(+57.02) |
| VPEELGKPTAPANLGAH | KNVEEAHMC(+57.02)FAKKL | KLKLLL | TVPDPRAQ | GC(+57.02)FDGWR | N(+42.01)SLDKAVARRHPVPVSPF | TM(+15.99)PSPKGS | KGQAQPNGEVGALP | EPNPTRASALPQ | GHEMEAGFKKKLEGHMQ |
| KASDGHNTYMWSF | TYHQPFLAF | TDDDYLM(+15.99)QVK | KC(+57.02)EDLGLQLDDDLGRLR | SYGC(+57.02)TNKPVPH | QAHASSGGM | VVGNGSKSC(+57.02)PRTLGGY | PTLGSAALSVQGESAFNLH | KPMSVTKKLSE | EVDVPADGGSFPGSC(+57.02)YGY |
| S(+42.01)GHANLSYKVQ | AGRGVENEGARTT | TDESSTLGM(+15.99)C(+57.02)GAK | PVLNKFHSSLK | ESLNSTGAQM(+15.99)LDQTPRVF | TLNAEQR | KLLASDNQPGTLENLR | L(+42.01)GKELPVVVLS | ASGMGLRVTTEESNFSPQ | LYASHRKTVLGNLN |
| KSAHVGPDFKLLVQWPQQRTEKQGPLF | AFNYGPSM(+15.99)VADRTPVTNY | TAVEM(+15.99)M(+15.99)PPLLL | KALNSVLL | PVVNLSFTTAQ | RLMSPVV | KVDAVESPPGSAGPA | EGAPKPTAGFAKSC(+57.02)VE | KGLATTVLL | TLVASHDTVVKLLHRPPQSM(+15.99)LALP |
| KNFHAPAPHLT | GSNETGAAPDEESQLVTVK | LPGPLAHLPVPA | VLLVAHGQPSTAA | S(+42.01)SAAKAASSSGWPFGGLR | T(+42.01)GGPLSVAKKPQAVPPHS | TSKMWAALSY | KFGAVM(+15.99)TLSVN | GHVTMVFPNLPGVVEKY | FSLVPAEPGSQR |
| QGDALTGKNSDT | ALPEEVLQVHWLVTPNGAP | KDTPAH | FRTQLF | TGAQANSLKPTVP | TTQVPSAR | KLLGGSPETVLSK | SFFNKMPHL | NVLVLPQ | LSAC(+57.02)TLVVPC(+57.02)NLDGAMPR |
| KDSLKLKAVGQLPVGSSL | RANGM(+15.99)SLNC(+57.02)FDEGLQEGR | VSLPMYSVPDPQLDQMPR | PFKAC(+57.02)SFRAAFFPKLF | KGM(+15.99)DETLAAPC(+57.02)T | TVENKC(+57.02)MTR | TGLC(+57.02)SPPK | TFPNQGSN | R(+42.01)EQDKQPSTLVT | ALGLNADMAPAVG |
| TPPSVM(+15.99)T | KPANKDP | HGGLADLSRTSSNGGHKAGQ | LHGLVPGAEHSTGFNVNLSL | ESPRHTDQPLKE | TVAHSFLKC(+57.02)VM | LPLVLQSA | KAYESHTPLP | ERSYFTGMNTLLGKPSVSKAQGR | SFLLKDE |
| KDTGGKGGKLM(+15.99)AGR | KGMNSVK | PGASPC(+57.02)SEKGTANP | PVNALKPTYPQF | KARVFF | LTANHLTAAPPHLELKNQ | VLGPTC(+57.02)AGVEC(+57.02)DLLWTV | S(+42.01)LGVAMYDGHADLLVTW | KLDLGPKN | PVVASLDS |
| QELSHAKGPLALADAPQ | KGSRVENTWVNHSTK | TGGEAAGPYM(+15.99)LAK | TSSSGHFELKE | TPDPPTNNLGVA | KTGTRFLAGGGPE | GYLAPPSVGGVVMLAASFVY | S(+42.01)LAGFAAPGLSVENTPHGSQ | PEEVRNLSPPHTAAKSQ | VTGFLNDPMGDDQPHLGVP |
| PTDVVLLLEGKFAVDFPQ | TLKLTE | TSLRLNQ | TVM(+15.99)M(+15.99)MPPLPL | KDGHKKWM(+15.99)QVEAR | TGAPKKPLP | SANYNFFLS | TGGLLSFKPPQGALPL | ANGLRVPQ | TVKDLTEFY |
| VRSDDEFNNYK | PM(+15.99)DDKGVH | TFGANLQF | AGGLLHALSPTM(+15.99)T | TKGM(+15.99)STQLNK | TEAPLNPK | TNLGKEFAPEA | R(+42.01)KGAAPTGNERC(+57.02)P | TVPTFPRKLK | KGKKSVQDNDPNVFP |
| TGKYFQR | AFYKLAT | KPLSGSVC(+57.02)LGPAADGAKNL | WSSPLLAVDATEAP | KRDTPVF | PAGGRFSLAEHE | TGAKNAAHA | TVFRFNAVW | LLAKGHDTYLP | QDAAKLLANATFNDVRGF |
| SPGGGVLTSK | KARVSLP | TRASVQQ | TVGTVGAGDLQSKAQ | A(+42.01)KDHGVFHHELVSL | SLNTVESL | A(+42.01)ANPSKVDFLPPGHS | PGPLLSVVLLS | TFNLKSQ | EC(+57.02)C(+57.02)RPC(+57.02) |
| FNLDKPVVLPL | TLLDDEGHAKGPL | KHLMVNKSQ | PEEVLAGLSTLVSGSALPDPV | TMLKPVGE | EPTAHRDTSPNGVTM | VGGLGVTLLQFPT | KALNC(+57.02)SYM(+15.99)LWDKQ | SFNLWST | AAPKSTLVQ |
| THVPGRC(+57.02)ALLVA | TSAPSLE | TNHVC(+57.02)SF | S(+42.01)PEVALLQHTFDLK | AGAGPTEGMGFFVFKTH | QPTFKLALLGVPDPTQK | TDDSM(+15.99)KPFNLR | EGFNPLGDASSSLER | QSLTREDSPPFHNP | KADGADELRKTDYLLQ |
| ETM(+15.99)HHAKKKSFTM(+15.99)VE | KLSNVPPP | TLGAMAGC(+57.02)C(+57.02)AALKP | SFMVNTT | TSSSPDDEAVNM(+15.99)R | KSWQHQ | TLAKGAVTAM(+15.99)TST | TYEGADYASAGK | KLSEYAPLEFH | KEFVTH |
| TGLVNRGDALH | TEGQSSTVDYVPF | TGKM(+15.99)NTMNVLA | VLLVSSQR | H(+42.01)KHEAFGMLPL | KAPWGDPLLAPHT | TVALEQLWKVS | TAAAPVSSQHARAQ | KANMSNPQ | TLNVAKAL |
| SVLYVTR | VSNLAPGKGGFVKLLTVGGE | HSLSHGVKC(+57.02)ADLFNFANA | SVTKNGPM | GPLEPTQR | TALRLNQ | TVAAGSEPTAKSL | VGGLALLHGVLVPQ | TLEALQR | QVVTLGSVPRMAL |
| KAAFASPPKTGK | K(+42.01)GGFGKVLDKGGF | PLHFSNKALVLL | A(+42.01)AAMFVTAKSPK | KNWHPGTFYPSLFVPN | KEDAPTVELTPEK | SNLTLPAL | TGRSHANT | KM(+15.99)GTRHVSGPVVR | GPGHADADPAGGLTEHDAHHG |
| TFSKNPLTEP | KDPVHHYFTAT | FLLVPA | HLEKKSPHSQVTLGHVEKHR | KM(+15.99)AKNPSKWPTE | KESSTPQ | GPAGPGPVMPVPPQEQSKR | AAVKAKALATSTSSF | TYALLTGPNFGSR | KAPHTELTRTPS |
| KEKKHLPYTL | EVDHFAALKTLPYHLQ | TVLYAQVRLE | KDVSQLAHLPNL | QPSSSLM(+15.99)DGAYGNLGGSLR | VVPDHHNM(+15.99)LTKALP | ETGRKYHDC(+57.02)PLQ | KPSGPTTTVLPET | NGFPGAC(+57.02)KLLNVQ | TAAYTGVKLSGM |
| TVGSSSKQGFLK | FLLVLVC(+57.02)C(+57.02)YGNALVLNR | N(+42.01)LAGGLSAAGEPDLLAGH | TVHSSKAF | TLAAPSH | SFGLAPD | FSGPAAVMEVKDK | KLTLTQ | SRYPMK | SKYGFNR |
| KAGLTTKVGPASGAK | SGGKYLTGS | QLLSDC(+57.02)GTKNQ | KNVLTAPMLW | REASALFVETVTPPSVK | TPVSKNGAVSLPL | A(+42.01)PGASLTTGQAKLEGGLRDY | KAFLFNVDK | QDELVVTQLDVPDVQ | SVLKPDPT |
| GHLPVTK | LPSEMTGNVVDA | NLTAPC(+57.02)DM(+15.99)HGT | DSVKVPGVTQC(+57.02)DYDD | TVRASQQL | TLVEHKFAAAE | SYAKNTPS | WVSYFVDAGAPKKC(+57.02)GGAGR | VPVEKTLP | TTPHNPGVQVAPAA |
| K(+42.01)M(+15.99)SSSPKMTMDADLQEGR | KGNGSPQSLTPEK | C(+57.02)STEPC(+57.02)VC(+57.02)QR | KPEDLT | PQNLFGKM(+15.99)VMVP | SADTHQGM | KAAC(+57.02)KPNRSAGAYEKGE | TVKDHVE | TPGPPVVLM(+15.99)KLQ | NDGFPGKF |
| TGPKSLT | GVMGPTGM(+15.99)V | KLLTM(+15.99)NSDGYGR | T(+42.01)PAGGNLLKLVSP | QAEQTANNLKPF | KANNTALVKFG | HSNLSTKDFH | TGFAFPHT | QKRGLLLQTHSNKE | LTQSSVLKSLEVASNSEPA |
| EGGHFTLWGFK | DTEC(+57.02)AKTMELEEKKQKKLTGDPV | KKAEPKLT | DAVSEAKMGPANNK | SHNKVKN | TSATM(+15.99)ESDDWN | GPAHSVTLP | TLLAKMM(+15.99)HDT | TDVQLLVELSKK | KAESVDDEFYVSF |
| KSRTPLSDGYLGR | TFKGPLVPRAE | KDLVTRLRSNK | AREYFLS | G(+42.01)KM(+15.99)C(+57.02)LLMVVYGVLTETY | ETSYAHTTKLPQ | KSDNQEDLM(+15.99)Y | KGAEWPL | EGAFKLLTGGQ | KEFAAPVRNQHSDKQ |
| TPGASPLRFMLP | KSTRGLC(+57.02)M(+15.99)TLA | ESFNLLTVGQPS | TEHHKRKAS | KFVYNPNPGTP | EVALNKGPVEAH | TDGHSQLT | A(+42.01)LAC(+57.02)MMAH | QVARTVEEPPTTLGAH | S(+42.01)SC(+57.02)PNDMHHGKE |
| G(+42.01)SALDLDNPGVPPAAVLSC(+57.02)VG | KAAPYVGVSSC(+57.02)GHL | N(+42.01)VTEHGAVLLPSAW | TPPKMRLEVRQ | N(+42.01)SAADQVEETDPAPE | LVGVELSKELQVAAR | LSAYLL | PSGTTRVPSGTGLHYTTGSPVRPMPT | S(+42.01)GAAHEAMPRAGQ | VVTVHQDVLPTP |
| TAPLVSLAP | TLGKDC(+57.02)DGEY | TVGPDNLLLLL | TVLLVVM(+15.99)PQF | SPDGWLDKFKKN | S(+42.01)VGGNPYGARVQ | LSVLDVDTAASQNLDQMPR | C(+57.02)GFDWGR | TVESTLNQQAAK | EGLLKDSAADAAKR |
| KPAVEGTTYVHR | TVGAMDMQSAEKL | FGPLPPGT | TGVKSEATLVPV | DPLEHNGSKQ | T(+42.01)VTGDDFFAVLQGF | SPVYGNN | TAPSVVTTKAE | KAVM(+15.99)LTNLLNVE | TSEM(+15.99)TVR |
| SLPTESVQ | ESKNM(+15.99)TVNLLLT | TLGASPNTL | KPGSGFDYKSHT | KAPFVLF | QPQLFGM(+15.99)M(+15.99)GFLAYT | AHDADTLLALLKAHNSLN | TAFLKSVP | N(+42.01)LSEEKKLLLDPH | TGVLVK |
| DPLNQSTDM(+15.99)LEEKQKKKMETGV | TAAAKC(+57.02)GGDPLN | A(+42.01)APVVVTLP | ERLYNF | S(+42.01)GSGLKYVLLPPN | FHGGLVLL | KLMVPKEDDSL | TTVKAALTT | TPLGAEKTNAF | TPSYLLQ |
| RDPLPTRYKL | TGKMKLE | K(+42.01)GKGDTYNKAL | GHHQQLNSDSPQC(+57.02)DKGAGK | TKGPEHYPGLP | K(+42.01)AC(+57.02)AQHHKALTT | LVAAKSLLT | KFPVAWAEGVLF | TVLQLLC(+57.02)RPC(+57.02) | SFPC(+57.02)HQLSH |
| QPVNPLHSDKPQ | TAFFDMKNGPA | TLSKAAPGC(+57.02)C(+57.02)ET | KDQQGQLLALM(+15.99)T | KNSSSSHTHPV | SPVQPLLA | SQAGLNTVPF | KAETLPRYLGKL | QEC(+57.02)HNFLKY | ERGDADHPKAGR |
| TDELAVC(+57.02)T | SGMNRVPC(+57.02) | TPAQLQVTM(+15.99)TAV | GTLAGVQLKAHNNPPSVKAR | TC(+57.02)KKYGH | SLTDQPC(+57.02) | VTNVERWGLLAFSVLSPK | ALDAYKRTELSH | TVGARC(+57.02)MFM(+15.99)PAK | SLEKDFP |
| SLAQGPC(+57.02)L | TLGSSHKE | QVSLTPATVWGF | QPGAAYFDKHSE | RTAAGHMLKEC(+57.02)MVYLPR | TTRAAPPHLSM(+15.99) | KAPQM(+15.99)LNEDQDMNQ | DAGKTHVNYADLN | KLAHELT | KSLKAAVR |
| KGGGSHGGPSFMDPVLT | NFEYVSF | TLLSANLC(+57.02)MEE | TLKESSSSPAVQ | TSPPVVLST | TVSGGLEVQLKSAG | TDSKSSSC(+57.02)PSR | G(+42.01)DPGAKFGS | KYLFL | SKKVTLC(+57.02)TMR |
| APDVLVFE | E(+42.01)GPLDVDGDPLRGNF | KGAPDDPVGWET | TVLVDANDEAH | TVVKNGDAAALE | SKMDM(+15.99)SF | KETNARMHEH | TVSSTTHKRVL | FGDVHVDL | LNKVDM(+15.99)C(+57.02)YN |
| LPGVAPPDDDEC(+57.02)LHKPH | KGGGAVPAAEM(+15.99)FK | AARDFPHKC(+57.02)RKGKEAR | TM(+15.99)LQVDE | SEAVELN | SQFFNPL | TAGKLSNSE | KVM(+15.99)STYP | FPTSPGAVFFAPPGGESKAQ | NAKPKPGC(+57.02)NTVQT |
| LPVKVNE | LAKDAVGTK | KAHALKKTTT | NKRSQPLQTKYGVVVFH | LRLTLL | KNPSLC(+57.02)EVSAH | KPGTYFPNAVVLQ | TQSLDPC(+57.02) | SMGVVTNN | ELAAAEEKTLEAK |
| L(+42.01)THLSM(+15.99) | TALQDVVM(+15.99)AMLPH | TGLLLLKM(+15.99)M(+15.99)N | KFNEYGH | TLELFE | QGLPRQPTVEPELGAH | PEEVVPGLPTVFN | APAFNEVMSPGGSLSYHSE | KQATTTVE | APGTTC(+57.02)KFLVPPQPSPVTF |
| LVFALTELPEKSSPELGAK | KSGAVNGTLWSLE | KAGPVVHVARDFP | LALEAGLLTSQP | QDERLC(+57.02)TMKLH | VHGLYGSGLLKPTDLLSK | QGSGLFARHNTLYM | KLM(+15.99)STPC(+57.02) | TVVTLPAASAYQ | SFAKC(+57.02)LN |
| TLKDALSVSNTPQ | RVGPSTTFGHTY | AAHALTAGD | G(+42.01)TAVYAALENKGSTLVAGGK | DPLEDSLKNYLLQQ | KDVSPKK | LVNFKGH | QLVM(+15.99)EHKVRSSSSFY | TGKSPFLE | NPSLAFHC(+57.02)KKVLGK |
| Q(+42.01)AAKPAASGKWAC(+57.02)DPGSPAGA | P(+42.01)SFHKTVLE | KGNGADPC(+57.02)GMM(+15.99)KYQ | TVNSSKLPASNL | TTVAC(+57.02)AER | KLRRTDDGY | HELFAFVPSRLNLVTVK | TPSNKGVAVSVVP | QDLFVLPA | S(+42.01)PLRHGAE |
| TSAAM(+15.99)LEPLELT | TLVVEM(+15.99)YAVLP | TELVVVS | TYVEFPRANVP | KQSSGPVK | DTSKC(+57.02)LVPADGPFGMLGK | TDM(+15.99)SEVR | STVQSSVSL | KNPVALLKYSAH | TALLNC(+57.02)TSKF |
| QLVLDFTVPPQ | E(+42.01)KLKP | KAALNGTDLFAK | SLEVPPFDAPDKNALNMAF | KTEVNADLQTR | LVAFTTPVKSAL | SNLTLGVF | TDGM(+15.99)SYTK | TVDFLAPS | TPVRSVAL |
| SGGSQSHLN | TVNGSHRFTVK | GNKKRPSQ | TPMLVKFKPAAVP | ELAGEM(+15.99)PTLKKSQPQ | LVSVELSKEQLR | STLHKTAP | LSKDGWGFGSKF | KSTSLDGEATKASAG | TFTYLM(+15.99)T |
| DDDYPPGVPAP | EVSVNYC(+57.02)ESLVAAH | T(+42.01)YLLYKC(+57.02) | TGDMSVK | GPAGKPSMM(+15.99)M(+15.99)GQAVPVVE | KDFRFFAR | G(+42.01)VGVAFGMHVLLAVPSAVAW | S(+42.01)GGYLVPQAAKPC(+57.02)AKLQ | APADTVYKLGGPPMVFFST | TVMNFPVPKL |
| KLQDKVTLDGYE | SLPNFTHAKVK | NLLVM(+15.99)HAAKGPAP | TLKQPPSH | TVGLSQGDAAWKADQETR | KGPQNEKPAFSS | TLRNPLSSPEQ | SEPM(+15.99)LTGL | PEC(+57.02)GPHDKAVH | TVGHKKTVFEK |
| KNQAYVTTHM(+15.99)TN | AVGGDLKEL | TTRVTATGVKHGGP | KPALSKALTKGAGPLKPL | DQLFPSVT | KPQLTKGELR | KVVRHEPAANK | KQPDLVVPPPQ | KLC(+57.02)C(+57.02)C(+57.02)C(+57.02)HGTL | KVDHSTKVYSNK |
| HNFAVVLL | PPQVLKFSFASQ | KAMAFPEAAEPFNLR | NPSAVLSL | SVAANKTKT | S(+42.01)SVLKTKKSLEVNPVPPF | TDKQPVRTSSY | S(+42.01)NLSRVPAAWLVMQ | QNSHLLT | TPSAVGNKVPLT |
| SDSLLPPFNVY | G(+42.01)SGEKRNT | KAALSVT | TTGTKLAGPKLQPQ | S(+42.01)SGFPLLSTKYQ | TTALKMESSPQ | K(+42.01)KLLLQ | GWELFK | TNKVPSSS | RESYFQAKFSVFNGGVGQ |
| KVMNLHSPVEPQ | QGVAKSSLDKATPQSAKQ | TYLQVEC(+57.02)ANN | KAALNNVV | KERGDTLVKEKAR | QPPTDKVL | TQAADFLLKPC(+57.02)P | TVDKTPLEFHSF |  |  |

**Supplemental peptide list 3** Short peptides of CK

| AAPPPMVKVHPEVPPR | AAPPPLWAHALEVPPR | AAPPPKLYMRDLPPR | AAPPPGKNHVREVPPR | AAPPPKNGHRVEVPPR | AAPPPSLSKYLEVPPR | LLADRGAAAWKLT | AANPPLVNSATAFVPPVG | AAPNPATFKNLDLPPR | TEAPLNPK |
| --- | --- | --- | --- | --- | --- | --- | --- | --- | --- |
| SSNKTALQC(+57.02)DLSSDD | QDSHSEDGVC(+57.02)C(+57.02) | TVEADKLVSPK | QC(+57.02)C(+57.02)C(+57.02)FT | DDC(+57.02)C(+57.02)C(+57.02)EDDP | KDRVVLP | QPLNPPKPAPKC(+57.02)NVVR | PPRC(+57.02)AASTSPPR | KFLVM(+15.99)F | PVNSHWL |
| AMFKKKLTPS | TTHKGPK | EDEELLLGLMSM(+15.99)SY | ANSKKKKSDK | APPFLLLLA | KKKAHW | KPC(+57.02)C(+57.02)MDMLLQR | V(+42.01)VFLLQSKS | EM(+15.99)YM(+15.99)SSKKKL | G(+42.01)C(+57.02)PLLLY |
| FTAKKM(+15.99)TE | DNLSKS | VVLLC(+57.02)NHW | V(+42.01)YSMYMPNYNYL | TGKMMVQL | ELLQAFQ | QKLVVPE | HEM(+15.99)M(+15.99)YT | KGKKYSST | A(+42.01)ARRRH |
| L(+42.01)GM(+15.99)SYH | TFKFTR | KALPPGSC(+57.02)GMQPDMDMPPFF | G(+42.01)KM(+15.99)NPPPL | KSDGPSDDMFM(+15.99) | YLVFPEPE | Y(+42.01)LVVTDPS | H(+42.01)VGPYLF | L(+42.01)LLFM | DC(+57.02)EKHLVVDR |
| K(+42.01)PVVDDDDED | M(+15.99)DDEKLPFKKAT | T(+42.01)TTLLVAQQ | HM(+15.99)YRVFKKK | EM(+15.99)DDDDDM(+15.99)MC(+57.02)E | D(+42.01)EKKKKT | QC(+57.02)C(+57.02)C(+57.02)FT | P(+42.01)PDLPRT | DNSDHDFKLLRC(+57.02)P | Y(+42.01)ERR |
| DDLLLPDDEADC(+57.02) | F(+42.01)LPPED | LVVALWAW | M(+42.01)GKKFKETQQN | L(+42.01)AHM(+15.99)PMMT | LLLLEA | G(+42.01)C(+57.02)DSDKQKP | HLPHTDFHTDLT | L(+42.01)AKAC(+57.02)HH | GMMNRR |
| HC(+57.02)C(+57.02)C(+57.02)C(+57.02)LLPRRVAA | H(+42.01)PPVTTEFVKPLSST | KVVVLM(+15.99)FS | KYDEETKMFPVLC(+57.02) | F(+42.01)DDFHNRM(+15.99)M(+15.99) | D(+42.01)EEKKKKSVM(+15.99)YS | K(+42.01)YYEAHHFG | K(+42.01)LLLLDDDDEN | V(+42.01)YATF | L(+42.01)AMDEKKKKKNP |
| YLLSLVM | T(+42.01)DMC(+57.02)PHNTRP | M(+42.01)GM(+15.99)KKKLPAQC(+57.02) | QALPYWE | WWFFAFLTM(+15.99)C(+57.02)TKSKFLLWV | GAHMMRVTTC(+57.02)M(+15.99) | C(+57.02)(+42.01)M(+15.99)DARRKKAMFC(+57.02)R | C(+57.02)(+42.01)C(+57.02)GSKKKKRGG | C(+57.02)MLLLV | LWKKKYPPS |
| FVVENWYFFM | A(+42.01)AKLPRYG | K(+42.01)LTWHKT | AFQQDY | D(+42.01)ALGPPWWC(+57.02)K | L(+42.01)LANHL | ADSC(+57.02)AMTM(+15.99)M(+15.99)C(+57.02)C(+57.02)C(+57.02)KKK | APMNDDTFFT | KTLPVS | A(+42.01)LSTAH |
| M(+42.01)FPPFH | LSTTADEDPPR | C(+57.02)DM(+15.99)DA | ELFTVYSH | L(+42.01)LEFQTLH | H(+42.01)LAC(+57.02)HLTHH | C(+57.02)SAM(+15.99)KKVVMV |  |  |  |

**References**

1. Suntornsuk W, Suntornsuk L, Feather degradation by *Bacillus* sp. FK 46 in submerged cultivation, Bioresour. Technol, 86 (2003) 239-243. https://doi.org/10.1016/S0960-8524(02)00177-3.

2. Sanghvi G, Patel H, Vaishnav D, Oza T, Dave G, Kunjadia P, Sheth N, A novel alkaline keratinase from *Bacillus subtilis* DP1 with potential utility in cosmetic formulation, Int. J. Biol. Macromol, 87 (2016) 256-262. https://doi.org/10.1016/j.ijbiomac.2016.02.067.

3. Abdel-Fattah A M, El-Gamal M S, Ismail S A, Emran M A, Hashem A M, Biodegradation of feather waste by keratinase produced from newly isolated *Bacillus licheniformis* ALW1, J. Genet. Eng. Biotechnol, 16 (2018) 311-318. https://doi.org/10.1016/j.jgeb.2018.05.005.

4. Jana A, Halder S K, Dasgupta D, Hazra S, Mondal P, Bhaskar T, Ghosh D, Keratinase biosynthesis from waste poultry feathers for proteinaceous stain removal, ACS Sustain. Chem. Eng, 8 (2020) 17651-17663. https://doi.org/10.1021/acssuschemeng.0c04378.

5. Sangali S, Brandelli A, Feather keratin hydrolysis by a *Vibrio* sp. strain kr2. J. Appl. Microbiol, 89 (2000) 735-743. https://doi.org/10.1046/j.1365-2672.2000.01173.x.

6. Falco F C, Espersen R, Svensson B, Gernaey K V, Eliasson Lantz A, An integrated strategy for the effective production of bristle protein hydrolysate by the keratinolytic filamentous bacterium *Amycolatopsis keratiniphila* D2, Waste Manag, 89 (2019) 94-102. https://doi.org/10.1016/j.wasman.2019.03.067.

7. Fang Z, Zhang J, Liu B, Du G, Chen J, Biodegradation of wool waste and keratinase production in scale-up fermenter with different strategies by *Stenotrophomonas maltophilia* BBE11-1, Bioresour. Technol, 140 (2013) 286-291. https://doi.org/10.1016/j.biortech.2013.04.091.
